# Pharmacological systems analysis defines EIF4A3 functions in cell-cycle and RNA stress granule formation

**DOI:** 10.1038/s42003-019-0391-9

**Published:** 2019-05-03

**Authors:** Alborz Mazloomian, Shinsuke Araki, Momoko Ohori, Amal M. El-Naggar, Damian Yap, Ali Bashashati, Shoichi Nakao, Poul H. Sorensen, Atsushi Nakanishi, Sohrab Shah, Samuel Aparicio

**Affiliations:** 1 Department of Molecular Oncology, BC Cancer, part of the Provincial Health Services Authority, 675 West 10th Avenue, Vancouver, BC V5Z 1L3 Canada; 2 Department of Pathology and Laboratory Medicine, G227-2211 Wesbrook Mall, University of British Columbia, Vancouver, BC V6T 2B5 Canada; 3 Research Department, Takeda Pharmaceutical Company Limited, 26-1, Muraoka-Higashi 2-chome, Fujisawa, Kanagawa 251-8555 Japan; 4 Department of Pathology, Faculty of Medicine, Menoufia University, Menoufia Governorate, Egypt; 5 Department of Epidemiology & Biostatistics, Memorial Sloan Kettering Cancer Center, 417 E68th St, New York, NY 10065 USA

**Keywords:** Systems analysis, Networks and systems biology, Checkpoints, Stress granules

## Abstract

The RNA helicase EIF4A3 regulates the exon junction complex and nonsense-mediated mRNA decay functions in RNA transcript processing. However, a transcriptome-wide network definition of these functions has been lacking, in part due to the lack of suitable pharmacological inhibitors. Here we employ short-duration graded EIF4A3 inhibition using small molecule allosteric inhibitors to define the transcriptome-wide dependencies of EIF4A3. We thus define conserved cellular functions, such as cell cycle control, that are EIF4A3 dependent. We show that EIF4A3-dependent splicing reactions have a distinct genome-wide pattern of associated RNA-binding protein motifs. We also uncover an unanticipated role of EIF4A3 in the biology of RNA stress granules, which sequester and silence the translation of most mRNAs under stress conditions and are implicated in cell survival and tumour progression. We show that stress granule induction and maintenance is suppressed on the inhibition of EIF4A3, in part through EIF4A3-associated regulation of G3BP1 and TIA1 scaffold protein expression.

## Abstract (web summary)

Alborz Mazloomian et al. use small molecule inhibitors to disrupt EIF4A3’s ATPase and helicase function which affects alternative splicing and nonsense mediated decay of transcripts. They define a genome-wide pattern of motifs of RNA-binding proteins associated with EIF4A3 and find that stress granules are downregulated upon EIF4A3 inhibition.

## Introduction

The eukaryotic initiation factor 4A (EIF4A) belongs to the Asp-Glu-Ala-Asp (DEAD) box superfamily of proteins that have ATP-dependent RNA helicase activity and are involved in various aspects of RNA biology, from transcription and translation to mRNA decay^1^. There are three paralogous genes of EIF4A, *EIF4A1,2,3*. Although EIF4A3 exhibits strong phylogenetic conservation between species and high homology at the amino-acid level to the translation initiation factors, EIF4A1 and EIF4A2, the confusingly-named EIF4A3 (also known as eIF4AIII, DDX48, NUK34, and hNMP 265) is actually a core component of the exon junction complex (EJC)^2,3^, the assembly of which is closely associated with splicing and does not play essential roles in translation initiation, in contrast to EIF4A1 and EIF4A2. Mutations in EIF4A3 have been found in Richieri-Costa-Pereira syndrome, an autosomal-recessive acrofacial dysostosis characterized by craniofacial anomalies and severe limb defects^4^. Moreover, zebrafish embryos with depleted levels of eif4a3 also exhibited a craniofacial phenotype.

The association of EJC core component MLN51 (for Metastatic Lymph Node 51, also known as hBarentsz (BTZ) or CASC3)^5^ stimulates the ATPase and RNA-helicase activity of EIF4A3, increasing its RNA-binding affinity^6^ and in conjunction with the splicesome, is deposited on mRNA in a non sequence-specific manner on the 5′ exon, 20–24 nucleotides upstream of the recently spliced out intron, hence marking the location of exon–exon boundaries on mRNA^7,8^. The binding of MAGOH and Y14, the other core components of the EJC, to EIF4A3 inhibit the ATP-dependent RNA-helicase function of EIF4A3 and conformationally locks the pre-EJC onto the mRNA^9,10^. Other non-core members of the multiprotein EJC, include SRm160/SRRM1, DEK, RNPS1 and ALY/REF^7^.

Nonsense-mediated mRNA decay (NMD) is triggered if the premature stop codon is at least 50–55 nucleotides upstream of the final exon–exon junction^11^. Therefore, memory of the exon junctions is crucial to the NMD process and the EJC is suitably placed to confer such a function. Indeed, EJC complex members, EIF4A3^12^, RNPS1^13^, UPF1-3^14^, Y14^15^, MLN51^12^ have been shown to play key roles in NMD and independent deletion of EIF4A3, Y14, MLN51, respectively, has been shown to affect an overlapping set of genes that are potentially regulated by NMD^12^.

Tumours are continually exposed to potentially lethal stressors such as oxidative stress, oxygen or nutrient depletion, endoplasmic reticulum or genotoxic stress, including cytotoxic therapy. To survive, tumour cells must adapt and such adaptation is postulated to underlie clonal selection and the acquisition of aggressive phenotypes which exhibit chemoresistance and metastatic capacity^16^. Key to this adaptation process is the shutdown of all non-essential protein synthesis, by temporarily storing their respective mRNAs in a silenced state in cytoplasmic stress granules, while only making essential proteins through selective translation of excluded mRNAs^17^. These cytoplasmic stress granules are aggregates comprising RBPs, the 40S ribosome, stalled translation initiation complexes, and silenced mRNAs which are stored until the stress is abated^18^.

While previous studies delineate the functions of EIF4A3 and the EJC by knocking out or down the genes encoding EIF4A3, there has been no studies where the specific helicase and ATPase activity of EIF4A3 has been targeted and the global effects studied. Here we show with novel specific and potent chemical inhibitors^19,20^ that graded-pharmacological inhibition of the ATPase and helicase activity of EIF4A3, a core component of the EJC, results in class-specific splicing defects and the monotonic increase of NMD prone transcripts of genes which are involved in a range of specific functions, including cell cycle (particularly G2/M checkpoint), chromosomal alignment and apoptosis. These data are consistent with previous EJC-knock out studies. Given the specificity of our compounds and the use of a control distomer, we were further able to demonstrate enrichment and/or depletion of RNA binding motifs on transcripts which were shown in our assay to exhibit a monotonic relationship on increasing doses of the EIF4A3 inhibitors. An unexpected finding is that pharmacological inhibition of EIF4A3 results in the abolishment of stress granules, uncovering a previously unknown role of EIF4A3 in this type of RNA metabolism and underscoring its importance in stress regulation and tumour biology.

## Results

### Global EIF4A3 dependent transcriptional processes

We set out to define the transcriptional, NMD and splicing dependencies of the EIF4A3 helicase, a core component of the EJC using graded pharmacological inhibition methods^21^ in conjunction with two recently discovered specific and active allosteric EIF4A3 inhibitors (T-595, T-202) that have similar scaffolds and a chemically identical but inactive stereoisomer compound (T-598) as a control^19,20^ and (Supplementary Note 1). While both allosteric eutomer inhibitors (T-595 and T-202) exhibited potent and specific inhibition of EIF4A3 activity in helicase unwinding assays and suppress nonsense mediated decay in reporter assays^22^ (Supplementary Data 1), the global transcriptional and splicing consequences of EIF4A3 pharmacological inhibition has not been previously described.

**Fig. 1:**
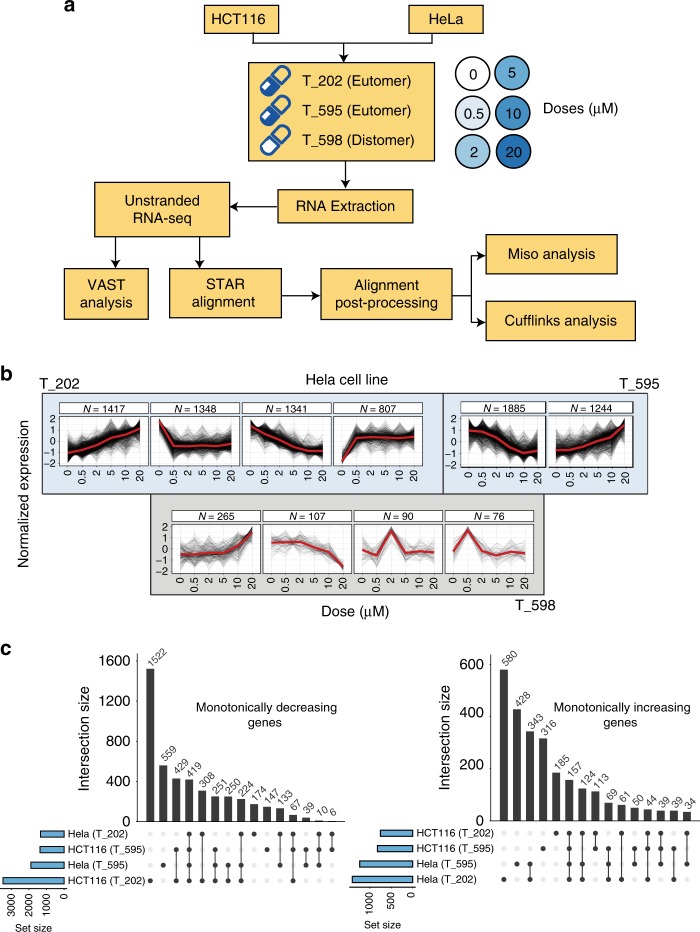
Global transcriptomic responses to graded EIF4A3 inhibition. **a** Workflow to study EIF4A3 functions in HeLa and HCT116 cell lines, treated with multiple concentrations of eutomers (T-595, T-202, blue shading), and an inactive distomer (T-598, white shading). Details in the Methods & Supplementary Note 1). **b** The top gene expression profiles when treated with eutomers (blue background) and the control distomer (grey background), plotted by normalized gene expression values (vertical axes) at respective inhibitor concentrations (horizontal axes). Each black line traces the response of a single gene, while the red lines represent the consensus response of all genes in the cluster. **c** Overlap between subsets of monotonically increasing (right) and decreasing (left) responses are shown for the eutomers. Each bar corresponds to the set of events only present in the cell line:inhibitor pairs determined by black circles

**Fig. 2:**
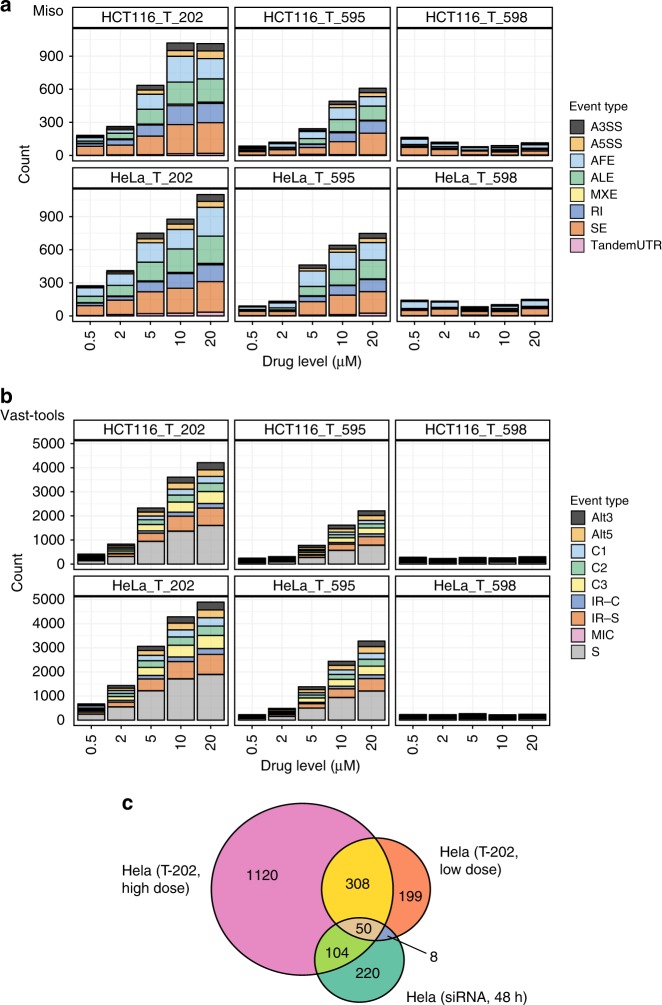
Characterizing alternative spliced events modulated by EIF4A3 inhibition. Counts of differentially spliced events (vertical axis) annotated by (**a**). MISO or (**b**). VAST-TOOLS at each inhibitor concentration (horizontal axis) for each cell line:inhibitor condition. Legend: Alternative 3′ and 5′ splice sites (A3SS, A5SS or Alt3,Alt5), alternative first and last exons (ALE, AFE), Mutually exclusive exons (MXE), Retained introns (RI), Skipped exons (SE), Tandem 3′ UTR (TandemUTR), Skipped exons stratified according to complexity of events (C1, C2, C3, S), micro exons (MIC) and intron retention events (IR-C,IR-S). **c** A Venn diagram displaying the overlap in HeLa cells of MISO identified alternative spliced events when *EIF4A3* in knocked down with siRNAs and treated with different doses of T-202 inhibitor

We analysed RNA-Seq reads from two mammalian cell lines HCT116 and HeLa, treated with increasing concentrations of drug at short duration exposure (6 h) for each compound (Fig. 1a and Supplementary Table 1). Key to subsequent analyses, we identified and quantified dose dependent monotonically responsive, transcripts (Fig. 1b, Supplementary Fig. 2, Supplementary Data 2 and 3) and alternative splicing events (Fig. 2, Supplementary Tables 2 and 3), NMD isoforms (Supplementary Fig. 1). Consistent with the chemical and pharmacological potency of the compounds, treatment of cells with both active eutomer allosteric inhibitors T-595 and T-202 resulted in a large number of monotonically increasing or decreasing transcripts in response to increasing inhibition, whereas treatment with the inactive distomer T-598 gave much smaller clusters of transcripts that showed no clear monotonic dose relationship (Fig. 1b and Supplementary Note 1). Across two cell lines a large proportion (~42 and ~47%) of monotonic responses were shared in common between two or more conditions across cell lines and compounds, indicating a high degree of conservation in EIF4A3 dependent transcriptional responses (Fig. 1 and Supplementary Data 3).

A large number of monotonically increasing NMD prone transcripts were found on treatments with both the active compounds, whereas no clear monotonically increasing cluster was observed on treatments with the chemically identical but inactive isomer T-598 (Supplementary Fig. 1a). We examined the ratio of NMD transcript expression to the corresponding gene expression and similarly observed the largest dominant clusters for both compounds represented monotonic increases of NMD prone isoforms over the genes (Supplementary Fig. 1b, Supplementary Note 2 and Supplementary Data 4). Moreover, the majority of monotonically responsive transcripts common in two or more conditions of absolute and isoform ratio comparisons also overlapped to a large degree (Supplementary Fig. 1 and Supplementary Data 4). These data are consistent with the dose-dependent inhibition of EIF4A3 by the eutomers resulting in a conserved (across different cell lines) monotonic increase in NMD prone transcripts.

### EIF4A3 dependent class-specific alternative splicing

Using this methodology of graded inhibition to investigate EIF4A3 dependent alternative splicing regulation, we interrogated the resultant RNA-seq dataset using two complementary computational methods (MISO^23^ and VAST-TOOLS^24^), in order to obtain wide coverage of alternative splicing events. We separately examined each inhibitor:cell line pair (details in Methods) and found that the two active compounds induce dose-dependent increase in the total number of MISO determined alternative splicing events (Fig. 2a). This trend is in contrast with the T-598 control-treated cells where only a small total number of events were observed (161 events predicted more than once, as compared to 1405 and 788 events in T-202 and T-595 treated cells, respectively), and no dose-dependent trend was apparent. The average increase rate in the number of events between any two consecutive inhibitions for the active compounds (averaged over the two cell lines and the two compounds) ranges between 0.17 and 1.43, with the maximum increase at 5 μM (compared to 2 μM).

Skipped exons are the most prevalent type of MISO observed alternative splicing event across multiple doses in both cell lines and the active inhibitors (*N* = 1279 unique events)(Fig. 2a). Alternative first exons (*n* = 807), alternative last exons (*n* = 649), and retained introns (*n* = 443) are the other abundant types of alternative splicing detected in our data sets, though only alternative splicing events previously annotated in the MISO knowledge base are visible and the relative abundance of alternative splicing types are therefore likely influenced by how comprehensively they are annotated in the MISO knowledge base, and more generally, in their overall prevalence (Supplementary Fig. 3). Accordingly, we normalized the number of events detected in at least one condition for each event type by the total number of events of the same type in MISO knowledge base, and found the highest ratio for retained intron events (7.4% of all retained intron events, where the ratio ranges between 1.4 and 5.9% for the other alternative splicing types, Supplementary Table 2). Similar to the MISO analysis, VAST-TOOLS alternative splicing event counts rise with increasing concentrations of the active compounds, and most of the detected events are skipped exons (7040 unique events in all of libraries, Fig. 2b, Supplementary Table 3, Supplementary Note 3 and Supplementary Data 5).

The much larger annotation database (~155,000 vs ~6000 introns, Supplementary Fig. 3) of VAST-TOOLS enabled us to identify many more retained introns (2680 unique events) compared with MISO (443 unique events). Also, more alternative 3′ and 5′ splice site events were reported by VAST-TOOLS compared to MISO (939 and 930 vs 250 and 185, respectively). A majority ~55% of MISO determined events overlapped with VAST-TOOLS determined events (Supplementary Fig. 4). Although pharmacological and RNAi mediated gene inhibition have different kinetics and efficiency, we compared the EIF4A3 inhibition alternative splicing event trends, grouped into high (2 μM) and low doses (0.5 μM), with EIF4A3 siRNA knockdown experiments (Supplementary Fig. 5) and observed that 42% (by MISO) and 38% (by VAST) of the events predicted in treatment:control siRNA comparison at 48 h were also detected by drug inhibition (Fig. 2c, Supplementary Fig. 6e, f and Supplementary Note 3).

### Analysis of EIF4A3 dependent alternative splicing and NMD

**Fig. 3:**
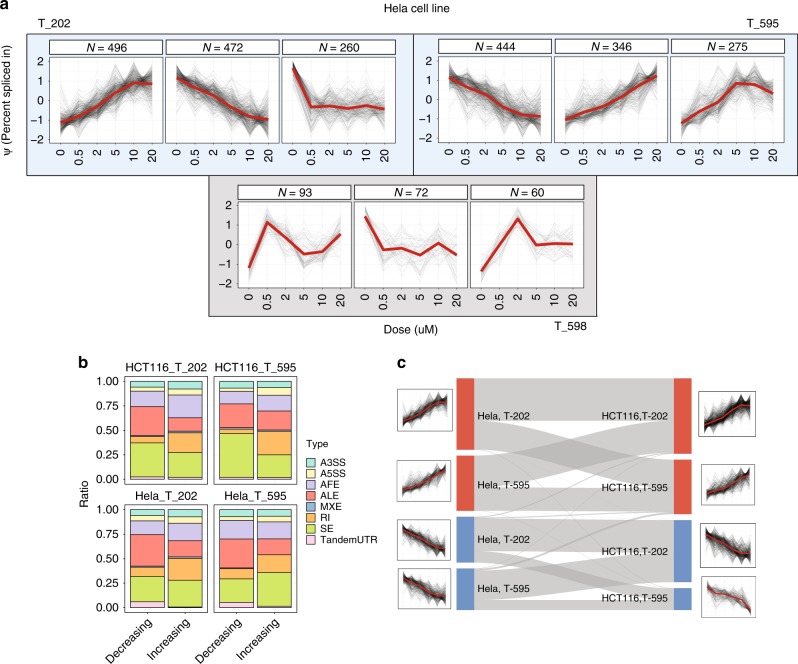
Clustering Ψ response profiles of alternative spliced isoforms using WGCNA. **a** The inclusion levels (Ψ values) of alternative spliced isoforms calculated by MISO are clustered using WGCNA. Background colours represent the clustering of Ψ profiles for the active compounds (blue) and the control (grey) compounds, respectively (the top three clusters are shown for each compound, sorted based on cluster sizes). Horizontal and vertical axes represent inhibitor concentrations, and MISO Ψ values. Each black line illustrates the Ψ response of one MISO isoform in a given condition, and the red lines represent the consensus response in each cluster computed by averaging Ψ values of all the isoforms. **b** Stacked bars, showing the cumulative ratios (vertical axes) of each alternative spliced type in the set of monotonically increasing and decreasing events (horizontal axes) for each cell line:drug pair. The composition of alternative splicing types is clearly different between the two sets. **c** A sankey diagram illustrating the proportion of shared events between monotonic sets identified in part **a**. Monotonically increasing and decreasing sets are shown in red and blue rectangles, respectively

To identify strongly EIF4A3 dependent splicing events in HeLa cells, we clustered Ψ (percent spliced-in ratio) profiles of all the identified MISO alternative splicing events to characterize alternative splicing events showing responses in agreement with EIF4A3 inhibition levels (Fig. 3a) and observed the two dominant clusters are sets of events with monotonically increasing (T-202 *n* = 496, T-595 *n* = 346) and monotonically decreasing (T-202 *n* = 472, T-595 *n* = 444) Ψ values. In comparison, the clusters set sizes where the control compound are used are much smaller and no monotonic trends could be observed. These trends were also observed in the libraries of the alternative cell line, HCT116 (Supplementary Fig. 7).

Moreover, we observed the majority of the overlapping events between the 4 drug:cell line (2 drug × 2 cell line) comparisons are concordant in response to EIF4A3 inhibition (Fig. 3c), consistent with the idea that these alternative splicing events are detected as a result of drug-induced EIF4A3 inhibition. On the other hand, the composition of alternative splicing types is clearly different between monotonically increasing and monotonically decreasing Ψ profiles (Fig. 3b). The clearest distinction is observed in retained intron type, where the proportion is on average 0.135 larger in the set of increasing profiles. Alternative last exon splicing also exhibits a clear variation between the set of monotonically increasing and decreasing events (average ratio of 0.165 and 0.289, respectively). There is a small yet statistically significant (Chi-square test; HCT116_T_202, *p*-value = 0.0009; HCT116_T_595, *p*-value = 0.02365; Hela_T_202, *p*-value = 0.0058; Hela_T_595, *p*-value = 0.02894, Supplementary Fig. 8) bias towards the proximal (5′) exons in the cluster of monotonically decreasing events upon EIF4A3 inhibition by both eutomers, suggesting a role for EIF4A3 in terminal exon transcript structure. Moreover, the structure of these transcripts are also distinct between monotonic increasing and decreasing clusters (see Supplementary Note 4).

To test the hypothesis that transcripts with retained introns would normally undergo NMD, however due to inhibition of EIF4A3-mediated NMD, they escape degradation and are detected, we identified alternative splicing events that may shift the reading frame (i.e. the skipped exon or retained intron lengths are not multiples of 3) and then checked whether monotonic events in a union set of MISO and VAST-TOOLS (Supplementary Fig. 9) were biased to be out of frame, compared with background events in MISO or VAST-TOOLS (Methods). We observed that the set of skipped exons with monotonic responses (both increasing and decreasing) and the set of retained introns only with monotonically increasing responses were significantly enriched by frameshift causing alternative splicing events (see Supplementary Table 4 (one-tailed Fisher’s exact test; monotonically increasing SE: *p*-value = 7.9 × 10^−13^, monotonically decreasing SE: *p*-value = 0.08; monotonically increasing RI: *p*-value = 0.04)). Moreover, only 15% of both MISO and VAST-TOOLS detected alternative splicing events overlapped with genes recognized to be NMD prone in our libraries (Supplementary Fig. 4), potentially denoting NMD independent functions of EIF4A3 in alternative splicing regulation. Taken together, the data are consistent with the notion that retained intron (and skipped exon) events are a function of modulation of the two EIF4A3-dependent processes—alternative splicing as well as a block of NMD.

Finally, genes with enriched alternative splicing events when treated with low or high concentrations of eutomers were classified by biological processes (BP) for network analysis. BP detected in low drug concentrations were almost a subset of the BP significantly affected in high concentrations of drugs (Supplementary Fig. 10, Supplementary Data 9). Differentially spliced genes are involved in similar BP in HeLa and HCT116 cell lines including RNA splicing, DNA repair, cell cycle regulation and 3′ end processing. When we did an intersect between the alternative splicing pathways, NMD pathways and gene expression pathways, we noticed that 81 and 78% of the pathways enriched by alternative splicing genes are also targeted through gene expression regulation and NMD regulation (Supplementary Fig. 11).

On the gene level, we observed little overlap between the genes which exhibit monotonic relationship with EIF4A3 drug doses that are involved in NMD, and those that are alternatively spliced (as determined either by MISO or VAST-TOOLS). These findings suggest that the vast majority of EIF4A3 generated alternative splicing (particularly skipped exons and retained introns) do not necessarily generate NMD-prone isoforms as would be expected, although a small subset of genes clearly exhibit this. This is consistent with the idea that EIF4A3 (and hence the EJC) may have independent roles in specific classes of alternative splicing events, namely skipped exons, retained introns and alternative last exons, in addition to its well-known role in NMD.

### RBP binding motifs on EIF4A3 dependent transcripts

The identification of distinct high-confidence EIF4A3 dependent splice reactions allowed us to investigate characteristic features (Supplementary Note 4) of and also to define the distribution of co-regulatory RNA binding protein (RBP) binding motifs on these transcripts. To examine the widest range of alternative splicing undergoing similar regulation (monotonically increasing *or* monotonically decreasing Ψ profiles), we looked at the union of such events in all 4 drug:cell line pairs of data. For each set of events, all annotated alternative splicing events in the VAST-TOOLS or MISO database not in the set were used as the control set.

**Fig. 4:**
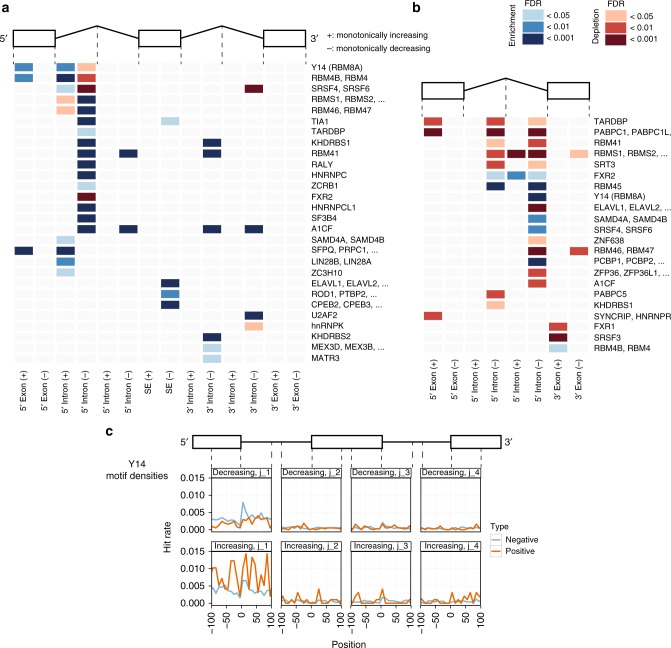
Motifs associated with regulation of alternative splicing by EIF4A3. Analysis of RNA binding motifs of known RBPs on EIF4A3 transcripts. Each row represents RBPs binding to a similar RNA motif, enriched (blue) or depleted (red) in the indicated region on the transcript (see Methods) in (**a**). skipped exon or (**b**). retained intron monotonically increasing (+) or decreasing (−) events as compared to background alternative spliced events. **c** Motif hit density of core EJC component Y14, on 200 nucleotide regions adjacent to skipped exon junctions. The vertical axes are the normalized hit rate the the motif can be found at the positions relative to the skipped exon junctions (horizontal axes). For the set of events with increasing and decreasing response profiles, normalized hits are compared against negative (background) samples

We investigated whether conserved regulatory features are encoded in EIF4A3 dependent alternatively spliced transcript sequences, adopting previously described motif enrichment approaches^21,25^. RNA motif density analysis of the most abundant classes, skipped exon and retained intron alternative splicing events, revealed the enrichment of RBP regulatory motifs in 5′ intronic regions of skipped exons for events with both increasing and decreasing Ψ profiles, and intronic regions of retained introns for events with monotonically decreasing responses (Fig. 4a, b, Supplementary Data 8).

For RBPs with known binding motifs, the position weight matrices (PWMs) from CIS-BP RNA database^25^ were used. RBPs from the same proteins were grouped together and the enrichment or depletion of them were assessed using Mann–Whitney *U* test. Motif hits of each set was compared to motif hits of the background set (see Methods for details)

For skipped exonic regions (Fig. 4a) both over and under representation of RBP motifs were observed in the 5′ intronic region of monotonically responding gene transcripts, consistent with the known function and location of EIF4A3 and the EJC at and upstream of splice junctions (or exon/intron boundaries). Interestingly, for retained introns (Fig. 4b) over or under representation of motifs was predominantly within introns exhibiting monotonically decreasing response profiles although the number of events in this category was much smaller.

Splicing-related RBPs, which are known to bind these enriched motifs, include RBM4, SFPQ, MBNL1-3^26–28^ (Fig. 4). A notable example is Y14/RBM8A, a core component of EJC which functionally cooperates with EIF4A3. This is one of the few RBPs with enriched binding motifs in both 5′ exons and 5′ introns of alternatively spliced exons of increasing responses (Fig. 4a). Consistently, 5′ intronic regions of events with monotonically decreasing responses are under represented with Y14 binding sites. The distribution of motifs around the splice junction shows a clear variation between these two classes (Fig. 4c) in close vicinity of the splice junction, particularly in 5′ most junction region, consistent with Y14’s known functional role in the EJC.

Taken together, the analysis of monotonic EIF4A3 dependent alternative splicing events demonstrates that they may be characterised by longer exons, shorter introns (Supplementary Note 4) and region-specific over/under representation of particular RBP motifs, features which may be used in future to classify the likelihood of EIF4A3 dependence.

### Effect of EIF4A3 inhibition on cell cycle and RNA-processing

We next sought to identify possible cellular functions dependent on EIF4A3 transcript isoforms, in two ways: first, by performing pathway analysis with gene ontology and second, by comparisons with previously published studies of EIF4A3 functions.

**Fig. 5:**
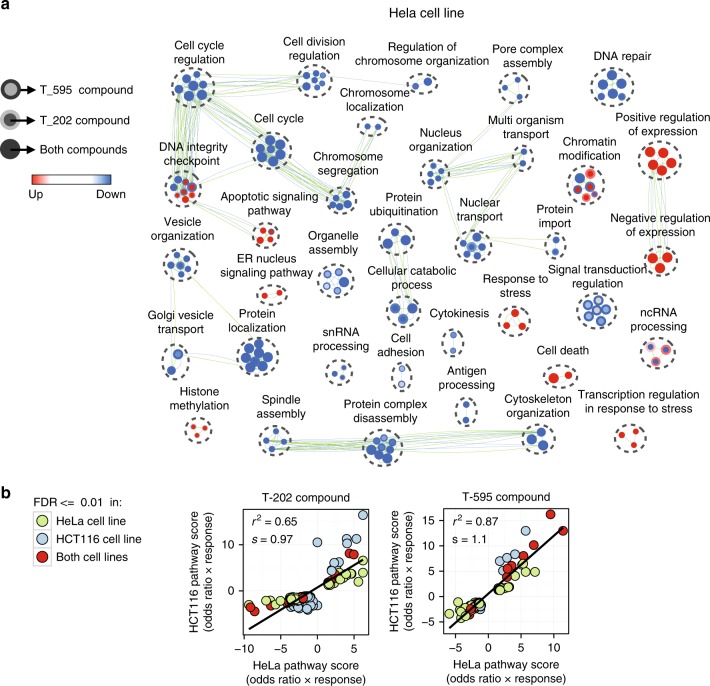
Biological processes affected most by EIF4A3 inhibition. **a** Enrichment map from gene ontology term enrichment analysis of biological processes. Each node represents a biological process with genes over-represented in the set of monotonically increasing (up, red)/decreasing (down, blue) clusters in the HeLa cell line. Node cores and node rings illustrate the results for T-202 and T-595 compounds, and green and blue edges indicate the overlap between the identified genes of gene sets for T-202 and T-595 compounds, respectively. Biological processes are clustered using EnrichmentMap^29^ based on the overlap of their monotonic genes. **b** A comparison between the enrichment scores (*x* and *y* axes) of GO terms identified in T-202 and T-595 compound libraries with FDR values < 0.01. Each point represents a BP set enriched in HeLa or HCT116 cell line data

We performed gene ontology term pathway enrichment statistical analysis (EnrichmentMap^29^, Methods) on three sets of data: (1) Gene transcripts which show monotonic dose relationships on EIF4A3 inhibition (Fig. 5a, Supplementary Data 11), (2) NMD transcripts that showed monotonic responses (Supplementary Fig. 12a, Supplementary Data 11), (3) NMD transcripts which show monotonic increasing trend of inclusion ratios (Supplementary Fig. 13, Supplementary Data 11).

For the monotonic dose transcript clusters, we observed a high degree of pathway/function concordance between the two active compounds (Fig. 5b) and also between the two cell lines, indicating that the overall correlation in EIF4A3 dependent cellular functions between cell types was relatively high, for both T-202 (*s* = 0.97, *r*^2^ = 0.65) and T-595 (*s* = 1.1, *r*^2^ = 0.87). The full list of genes can be found in the Supplementary Data 9.

Among the most prominent regulated processes were those connected with cell cycle regulation and checkpoints (e.g. *CDC27* (Supplementary Fig. 14)) (Supplementary Figs. 12a and 13a). Interestingly, both up and down regulated cell cycle checkpoint processes were observed, alongside down-regulated clusters enriched (used criteria: FDR < 0.01, hypergeometric test) for functions in cell cycle, cytokinesis, cell division, chromosome localization, spindle assembly/organization, DNA repair and protein localisation. These are all functions which can be associated with disordered regulation of cell division checkpoints. In contrast, up-regulated clusters were enriched (used criteria: FDR < 0.01, hypergeometric test) for stress responses, ER function, apoptosis components and chromatin modification functions (Supplementary Data 11).

The gene ontology pathway enrichment analyses for the genes with NMD-prone transcripts that showed monotonic responses and similar analyses on NMD transcripts with monotonic inclusion ratios also involved cell cycle and related pathways but also appeared to be enriched in RNA splicing and processing pathways (Supplementary Figs. 12a and 13a). There is again a notable concordance between the results of the two cell lines (Supplementary Figs. 12b and 13b).

The pathway analyses suggested strong conservation of several functions, with overlap to previous studies of EIF4A3 on specific transcripts, as well as global analysis using siRNA knockdown^12,30^. In our dataset, for example, *MAPK6* (ENSG00000069956) was found to be consistently downregulated in both HeLa and HCT-116 cells (Supplementary Fig. 15a), whereas *MAPK7* (ENSG00000166484) was respectively found to be upregulated by the active inhibitors of EIF4A3 and not by the inactive distomers (Supplementary Fig. 15b) consistent with previous studies (See Supplementary Note 5 for further examples).

We enumerated overlaps between our dataset and where available, global RNA-seq from EIF4A3 knockdown previously published (Supplementary Data 12 and Supplementary Note 6 for in depth comparisons). We also compared between the different methods (MISO, VAST-TOOLS and DiffSplice), annotated each event and summed events by genes and then compared only the human genes in Wang et al. and our studies using the three different methods (Supplementary Fig. 16). Significantly, 64 genes were common in at least 3 different experiments over the two separate studies and *AURKB* was among these 64 genes while 7 genes appeared common in all the four comparison (including *SRSF2*)(see Supplementary Note 6. Gene lists are can be found in Supplementary Data 12)

Among the overlapping cell cycle/apoptosis and RNA processing genes identified by gene ontology term analysis, we found several examples of previously described EIF4A3 dependent splicing events, *AURKB*^31^ and *BCL2L1*^31,32^ and *SRSF2*. Our dataset reveals dose-dependent effects for each of these transcripts as previously described in the literature further validating the specificity of the inhibition of the EIF4A3 and the drugs (See Supplementary Note 6 for more details).

### EIF4A3 is involved in cell cycle and stress granule pathways

**Fig. 6:**
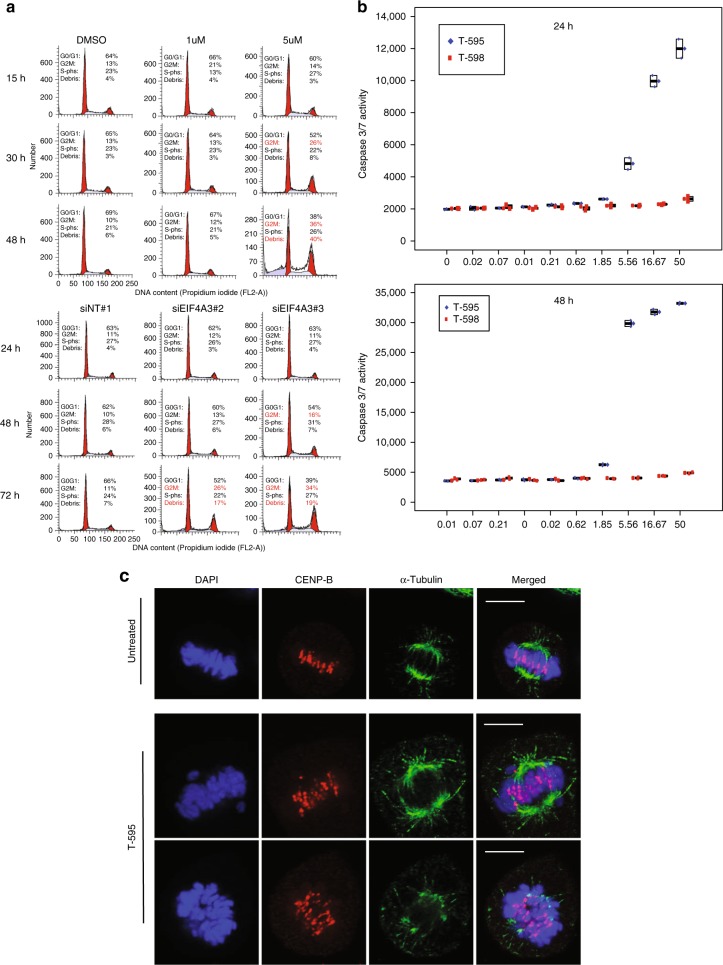
Biological effects of treatment with EIF4A3 inhibitor, T-595. **a** Treatment with T-595 at high doses or with siRNA to *EIF4A3* and after 48 h results in G2M arrest and sub-G1/G0 fraction indicative of apoptosis. Cell cycle profiles (counts (vertical axes) vs DNA content (horizontal axes)) were assessed by flow cytometry after the treatment DMSO, 1 or 5 μM T-595 or siRNA (siNT#1, siEIF4A3#2,#3) for the respective times shown. The percentage of cells assigned to be in the respective stages of the cell cycle is indicated on the respective charts. **b** Apoptosis induced on treatment with eutomer, T-595 but not distomer, T-598. Graphs showing apoptosis as assessed by Caspase 3/7 activity (vertical axes) when HeLa cells were treated for 24 h with various concentrations of T-595 (blue) or T-598 (red) (horizontal axes). **c** Microscopic visualization of spindle and chromosome proteins reveals chromosome mis-segregation. Representative images of untreated (top panel) and treated (5 μM T-595 for 30 h, bottom two panels) showing chromosomal DNA stained with DAPI (blue), centromere proteins stained with anti-CENP-B (red) or microtubules identified by anti-*α*-tubulin (green). Scale bar = 10 μm

The global systems analysis and specific genes validated above strongly implicated cell cycle, spindle assembly, chromosome segregation and checkpoint processes that might be affected by reduced EIF4A3 function. To validate in vitro, the biological relevance of the gene ontology term network analysis we measured the cell cycle responses and apoptotic fraction by flow cytometry, in response to increasing doses of the active and control compounds or siRNA knockdown of EIF4A3 (Fig. 6a). Consistent with the network analysis, this revealed that prolonged and increasing inhibition of EIF4A3 (doses > 5 μM and >15 h exposure) resulted in increased apoptosis (as assessed by caspase-3/7 activity, Fig. 6b, Supplementary Data 13), associated with a cell cycle arrest at the G2M boundary. Timed microscopy of cells (Fig. 6c) with immunofluorescence staining for spindle and chromosome centrosome proteins (*α*-tubulin, CENP-B) revealed frequent chromosome mis-segregation and abnormal spindle assembly. Taken together, these data suggest that disrupted cell cycle associated with chromosome segregation abnormalities and a G2/M checkpoint induction, in addition to apoptosis are consequences of EIF4A3 inhibition and do not appear to result from the direct inhibition of tubulin polymerization by any of the compounds (Supplementary Fig. 17).

In addition to the known roles of EIF4A3 in NMD, we identified stress pathways from network analysis and hypothesised that EIF4A3 inhibition might affect the cellular stress response pathway (see Supplementary Note 7). To explore this we examined a core set of 63 canonical stress granule associated genes assembled from the literature^33,34^ and experimentally validated in our laboratory (Supplementary Table 5), contrasting transcripts affected by the eutomers (T-202 and T-595) but not the distomer (T-598). Considering eutomeric responsive transcripts, either up/downregulated in overall abundance and/or exhibiting changes in NMD transcript isoforms and/or exhibiting altered NMD transcript ratios, we observed that 37 of the 63 stress granule associated genes satisfied at least one differential regulation condition (Supplementary Table 6). Interestingly, there is only one stress granule pathway associated gene transcript (*BICD1*) that is upregulated by both T-202 and T-595 in HeLa cells, whereas 10 genes that had their transcripts downregulated in HeLa or HCT-116 cells. Furthermore, when we examined the NMD prone transcript isoforms of these genes (shaded in Supplementary Table 6), 12 out of 18 of NMD prone transcripts were shown to exhibit a monotonic increase in both cell lines in response to both active eutomers. Seven of these genes had NMD isoforms and also demonstrated splicing changes when treated with the active EIF4A3 inhibitors (Supplementary Table 6).

**Fig. 7:**
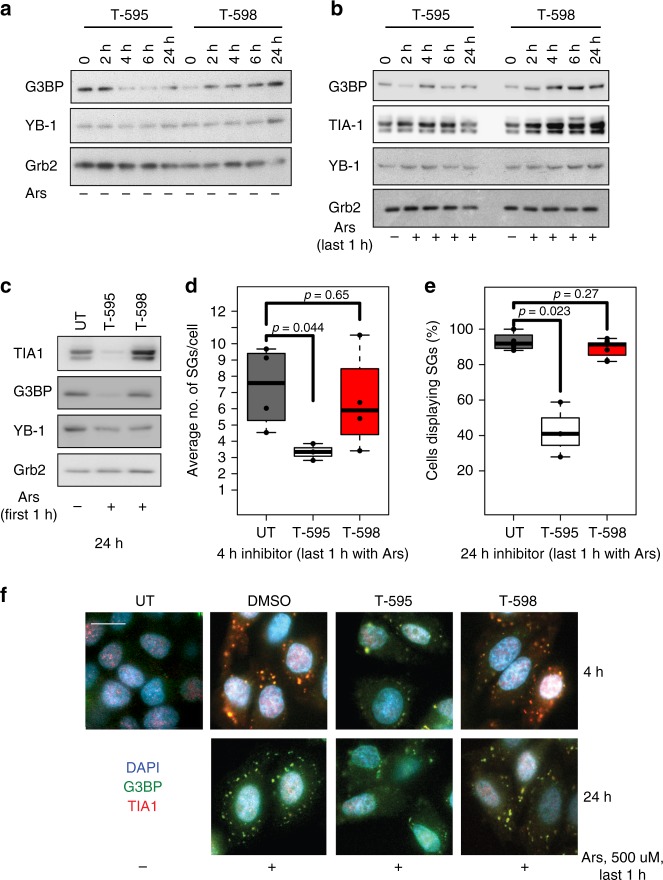
EIF4A3 and stress granule formation. **a** The effect of T-595 and T-598 on stress granule marker accumulation. HeLa cells were treated with 10 μM compounds (T-598, or T-595) for the indicated time **a** alone or **b** with sodium asernite (Ars, 0.1 mM) added either for the last 1 h or **c**. after the first hour and then incubated for the remainder of the experiment then lysed and probed with the respective antibodies as indicated, by Western Blotting. Stress granule quantification and visualization in HeLa cells with the indicated markers (DAPI for DNA, G3BP and TIA-1 for stress granules (SGs)) on treatment with 10 μM compounds (T-595 or T-595) or DMSO (UT) as control, with 0.5 mM sodium arsenite added **d** 1 h prior to harvest or **e** treated only for the first hour and then incubated without for 24 h. **d** Average number of SGs per cell at 4 h or **e** the total number of cells displaying ≥3 SGs at 24 h were reported or **f** representative images captured on a confocal microscope at the indicated times. Scale bar = 10 μm

We further validated this observation by assaying the protein expression of two key stress granule anchor proteins, the Ras-GTPase-activating protein SH3 domain-binding protein 1 (G3BP1)^35^ and T-cell intracellular antigen (TIA-1)^36,37^. Over a 24 h time course, we observed that T-595 but not T-598 caused a progressive decrease in protein expression of the steady-state expression of these two stress granule markers (Fig. 7a). This is not a global effect on all stress granule associated proteins as the levels of YB-1, a stress and hypoxia induced factor that has been reported in stress granules^38^, did not appear to be affected by the compounds. When stress granule formation was induced by addition of 100 μM of Arsenite in the last hour before cell harvest at each time point over 24 h, pre-treatment with the T-595 but not T-598 appears to block G3BP and TIA-1 protein expression as markers of stress granule (Fig. 7b). While we were not able to dissect the exact mechanism, as the splicing event was common between protein coding and NMD transcripts, we were able to observe that exon 5 of *TIA1* (ENSG00000116001) appears to be skipped in a dose-dependent fashion in response to T-595 but not T-598 in HCT-116 (Supplementary Fig. 18a–f) and alternative splicing of TIA-1 occurs in HeLa (Supplementary Table 6). We nonetheless observed the various NMD transcripts of both G3BP1 and TIA-1 increase with the two active eutomers in the cell lines tested (Supplementary Table 6), in correlation with their observed decrease in protein levels (Fig. 7a–c).

We next assayed individual stress granule formation using immunofluorescence with the stress granule markers in HeLa cells treated with a higher dose (500 μM) of arsenite in two scenarios—late induction (1 h before harvest) and early induction (induction for 1 h followed by EIF4A3 inhibitor treatment). In the late stress granule induction experiments, significant reduction (Fig. 7d–f) in the formation of punctate granules of G3BP and TIA1 occurs at 24 h with T-595 eutomer (<50% granules, *p* = 0.02, two-tailed Student’s *T*-test) but not T-598 distomer (*p* = ns, two-tailed Student’s *T*-test) consistent with the lack of induction of stress granule proteins by western blot under T-595 eutomer inhibition (Fig. 7b). This effect was also visible and significant at early drug exposure time points (4 h, Fig. 7d, *p* = 0.044, two-tailed Student’s *T*-test). As with late induction, we observed that early induction of stress granules followed by inhibition by T-595 but not T-598 (Fig. 7c) was able to effectively block stress granule protein marker expression, indicating that maintenance of stress granules also requires functional EIF4A3. Taken together these data support a role for EIF4A3 in the formation and maintenance of stress granules in the cell after stress (see Supplementary Note 8).

## Discussion

Here we identify the encoding of EIF4A3 splicing and NMD isoforms using graded pharmacological inhibition of EIF4A3 with novel allosteric small molecule inhibitors, whose chemical synthesis, efficacy and specificity we have recently described^19,22^ (see Supplementary Note 9). With graded inhibition in two different cell lines we show that the core transcriptional response, alternative splicing reactions and NMD prone isoforms exhibit a high degree of conservation between cell lines, and a significant degree of overlap with our own and previously obtained RNA-seq from siRNA inhibition of EIF4A3 with few exceptions (Supplementary Note 10). The underscores the drug specificity in vivo and methodology used in this study.

The identification of monotonically affected NMD isoforms is consistent with the known involvement of EIF4A3 in EJC formation and in NMD^9,12,22,39^. Most NMD-prone transcripts identified were up-regulated by eutomer treatment reflecting a stabilization of these transcripts by EIF4A3 (and EJC) inhibition; very few transcripts were downregulated as NMD transcripts were normally degraded without drug treatment^40^. This suggests that EIF4A3-dependent NMD may only affect a subset of NMD-annotated transcripts^12^ and the common set of genes corroborate this finding (Supplementary Fig. 4 and Supplementary Table 7a).

We observed enrichment of specific classes of alternative splicing (skipped exon, retained intron and alternative last exon events) in monotonically increasing clusters formed by eutomer treatment. The many genes involved in splicing (Supplementary Fig. 10, Supplementary Data 11) and the distinct pathways involved in both processes regulated by EIF4A3 (Supplementary Figs. 10–12) support distinct and overlapping roles for EIF4A3 and the EJC in the process of splicing and NMD (see Supplementary Note 11 for specific examples) and further add to the complexity involved (see Supplementary Note 12 and Supplementary Table 7 for gene lists).

A recurrent theme of RNA processing is the modulation of trans-acting factors, RBPs that may affect which cis-acting binding motifs they bind. Our study has for the first time, defined the structure (Supplementary Note 13) and the RBP features associated with EIF4A3 reactions transcriptome wide. It is notable that the motif patterns we uncover on EIF4A3 dependent transcripts are distinct from motifs enrichment patterns of other splice-factors such as CLK2 (Supplementary Note 14). Our systems-level approach has identified an experimentally-validated Y14 binding motif^25^, which is enriched in certain EIF4A3-depdendent transcripts and depleted in 5′ introns of others (Fig. 4a), within the vicinity of splice junctions (Fig. 4c) as previously shown^41^ and in agreement with the known role of Y14 within the EJC. Further validation is required to demonstrate whether Y14 interacts directly or indirectly with EIF4A3-dependent transcripts identified in this study as the Y14 binding motif is believed to be unavailable for RNA binding in its interaction with MAGOH in the EJC complex (Supplementary Note 15).

Using the EIF4A3 dependent features identified above and the network analysis of gene functions we identified, we experimentally validated the role of EIF4A3 in cell cycle checkpoint function. EIF4A3 inhibition, either with compounds or siRNA, results in G2/M arrest, in conjunction with increased apoptosis, with demonstrable centrosome-spindle disturbances such as mis-segregated chromosomes (Fig. 6). This is consistent with previous experiments showing that siRNA inhibition of EIF4A3 and CDC27 (whose NMD-prone transcript is monotonically stabilized when EIF4A3 is inhibited—Supplementary Fig. 14). Moreover AURKB inhibition (whose alternatively spliced, NMD-prone transcript shows dose dependent increases—Supplementary Fig. 19) also results in mitotic defects in HeLa cells^42^ (Supplementary Note 16 for several other examples). As previously shown^32^, inhibition of EJC results in the enrichment of pro-apoptotic *BCL-X*_*S*_ transcript (Supplementary Fig. 20), consistent with the apoptosis observed when EIF4A3 inhibitory eutomers are applied to cells at higher doses and longer time points (Fig. 6).

Among the RNA processing functions identified, we also discovered an unprecedented role for EIF4A3 in RNA stress granule formation (See Supplementary Note 8 for summary). We observed the NMD transcripts of *TIA1* and *G3BP1*, increase with the two active eutomers in the cell lines tested (Supplementary Table 6) and this correlates with depletion of their respective protein levels. TIA1 and G3BP1 are not only the most widely used markers but also key scaffold proteins in stress granule formation, which we show to be abolished on EIF4A3 inhibition (Fig. 7). Moreover skipped exon 5 of *TIA1* appears to respond monotonically only to the active eutomeric inhibitor of EIF4A3 and as would be expected with the increase of NMD transcripts and loss of TIA-1 protein levels, we find enrichment in the binding motif of TIA1 on monotonically increasing skipped exon transcripts on EIF4A3 inhibition (Fig. 4a). Therefore, these findings implicate EIF4A3 in stress granule formation and maintenance.

In tumour biology and metastasis^43^, stress granule formation may provide a selective advantage to the cancer or clones of cancer cells that can adapt to growing under conditions of stress through the temporary and selective sequestation of a subset of mRNAs^17^ in stress granules until the stress is abated^18^. Our findings that EIF4A3 (of the EJC) is important for stress granule biology and that the splicing of *TIA1*, a key factor involved in stress granule formation may add a new mechanistic link (Supplementary Note 17) and guide future work (Supplementary Note 18).

## Methods

### Transfection of siRNAs

Silencer™ Select siRNAs targeting EIF4A3 were purchased from Life Technologies (Thermo Fisher Scientific, Waltham, MA, USA) and the sequences are found in Supplementary Table 8. Twenty four hours after HeLa cells seeding, siRNAs were transfected using Dharmafect 1 (GE Healthcare, Chicago, IL) according to manufacturer’s instructions at a final concentration of 10 nM. Non silencing siRNA was used as the negative control. Knock down was experimentally validated (Supplementary Fig. 5). After 72 h of incubation, cells were harvested for RNA-seq and total RNAs were extracted using an RNeasy Miniprep Kit (Qiagen, Valencia, CA).

### Antibodies

The following antibodies were used in the current study: G3BP (stress granule marker, mouse monoclonal, BD Transductions, 1:1000, 611126), GRB2 (mouse monoclonal, BD Transductions, 1:1000, 610111), TIA-1 (stress granule marker, rabbit polyclonal, Abcam, 1:1000, ab40693), YB-1 (59-Q, mouse monoclonal, Santa Cruz Biotech., 1:500, sc-101198), CENP-B (rabbit polyclonal, Santa Cruz, Dallas, TX, 0.5 μg ml^−1^, sc-22788), *α*-tubulin (mouse monoclonal, Sigma-Aldrich, St. Louis, MO, 7.5 μM, T9026).

### Cell lines

Both cell lines were sourced commercially from ATCC; HeLa (ATCC CCL-2) and HCT1-116 (ATCC CCL-247). The cell lines were cultured in McCoy’s 5A Medium Modified (Gibco, ThermoFisher, MA, USA) or Dulbecco’s Modified of Eagle’s Medium (Corning, VA, USA), maintained at 37 °C at 5% CO_2_ and routinely tested negative for mycoplasma contamination. The HeLa cell line used found by STR testing (Genetica, NC, USA) to be 100% matching to ATCC CCL-2 (HeLa). The HCT-116 cell line was a 76% match to ATCC CCL-247 HCT-116 due to genomic instability as this line is known to be mismatch repair deficiency and hence microsatellite unstable. This does not affect the conclusions of the study since we used two unrelated (cervical and colorectal) mammalian cell lines to control for line specific effects.

### Treatment with compounds

Twenty-four hours after HeLa cell seeding, cells were treated with each concentration of compound for 6 h. Cells were harvested for RNA-seq and total RNAs were extracted using an RNeasy Miniprep Kit (Qiagen).

### Apoptosis assay

After 24 h of incubation with the various compounds, caspase-3/7 activity was determined using the Caspase-Glo 3/7 Assay (Promega, Madison, WI) according to the manufacturer’s instructions.

### Immunofluorescence

Cells were fixed in 4% of paraformaldehyde for 15 min at room temperature followed by permeabilization with 0.1% triton buffer. After blocking with 5% BSA, cells were incubated with primary antibody over night at 4 °C. Anti-CENP-B antibody (sc-22788, Santa Cruz, Dallas, TX) and anti-alpha tubulin (T9026, Sigma-Aldrich, St. Louis, MO) were used at concentrations of 0.5 μg ml^−1^ and 7.5 μM, respectively. After washing with PBS three times, the cells were incubated with Alexa Fluor 488 anti-mouse IgG or Alexa Fluor 594 anti-rabbit IgG (Life Technologies, Carlsbad, CA) at a concentration of 2 μg ml^−1^ for 1 h at 37 °C, and were mounted with Vectashield (Vector Laboratories, Burlingame, CA). Cells were imaged on LSM700 (Carl Zeiss, Oberkochen, Germany).

### Cell cycle analysis by flow cytometry

HeLa cells were treated with 1 or 5 μM of T-595 or were respectively transfected with single siRNA (#1, #2, or #3) against EIF4A3 or control siRNA (#1 or #2) for the indicated time. Cells were washed twice with ice-cold PBS, and treated with Cycle Test Plus DNA Reagents (BD Biosciences, Franklin Lakes, NJ) in accordance with the manufacturer’s instructions. The cellular DNA contents of single cells (gated as shown in the Supplementary Fig. 21) were determined by flow cytometry on a BD FACSVerse™ (BD Biosciences, Franklin Lakes, NJ). The percentage of cells in each phase of the cell cycle was analyzed by ModFit software (Verity, Topsham, ME). The abundance of cells in the post-sort fraction was typically over 90% of the population by analysis of ModFit software.

### Data acquisition and analysis software

The RNA-seq libraries were sequenced on a HiSeq 2000 (Illumina, CA, USA) using the manufacturer’s software and protocols. RNA-seq reads were aligned with STAR aligner (STAR_2.5.1b_modified). SAMtools (version 0.3.3) was used to remove duplicate reads. Transcript abundances were estimated by Cufflinks (v2.2.1). The expression output values of Cufflinks were normalized by edgeR package (3.8.6). MISO (0.4.9) and VAST-TOOLS (1.0.0-beta.2) were used to perform the splicing analysis. WGCNA (v.1.51) framework was applied to cluster genes exhibiting correlated response patterns. Gene set enrichment analysis (GSEA) was carried out on a set of BP from MSigDB (v.5.2). For analyzing motif enrichment PWMs were downloaded from CIS-BP RNA data base. R and Python were used to do the analysis and perform statistical tests also generating figures using ggplot2 and matplotlib accordingly. All the complementary codes written in python and R are uploaded to a github repository (https://github.com/BCCRCMO/EIF4A3public) and are available upon request.

### Alignment and calculating gene and isoform expressions

The RNA-seq libraries in this study consist of 100 nucleotides paired-end unstranded reads from a HiSeq 2000 (Illumina, CA, USA). By using the STAR aligner^44^, the short reads were aligned to the human reference genome (hg19) downloaded from the UCSC genome browser^45^. Following the alignment step, duplicate reads were removed by employing SAMtools^46,47^, and the remaining reads were served as the input to the expression and splicing analyses.

Transcript abundances were estimated by using Cufflinks^48^. The Cufflinks FPKM (fragments per kilobase of transcript per million reads mapped) values were calculated separately for each library with the annotation file (.gtf) downloaded from Ensembl (GRCh37)^49^. The Cufflinks parameters *frag-bias-correct* and *multi-read-correct* were enabled in this step. The expression values of libraries generated by treating the same cell line by different concentrations of the same compound were normalized using the upper quartile normalization method of the edgeR package^50,51^, prior to clustering gene and isoform response profiles.

### Alternative splicing analysis

All RNA-Seq samples had a minimum of 100 million reads (see Supplementary Table 1) reads except in the siRNA study where three biological replicates for each time point (TK0018-TK0020 - 24 h and TK0022-TK0024 - 48 h) which were respectively pooled and the siRNA controls (TK0017 and TK0021) pooled to ensure there were sufficient reads to perform MISO and VAST-TOOLS analyses.

We first used MISO (Mixture of Isoforms)^23^ framework to find AS events when samples treated with compounds were compared to untreated (control) samples. MISO classifies AS events into 8 AS types and for each candidate event, it assigns a Ψ (Percent Spliced In) value (between 0 and 1) representing the inclusion ratio of an isoform in a library. When comparing two conditions, a Bayes Factor (BF) value is reported as a measure of confidence of an event being differentially spliced between the two conditions.

When clustering MISO Ψ values, all reported events were considered. To find AS events induced by compound treatments, treated samples were paired with control samples, and events with |ΔΨ| < 0.1 or BF < 10 were filtered.

In addition to MISO analysis, VAST-TOOLS^24,52^ framework was also applied to further explore AS regulation in RNA-seq libraries. VAST-TOOLS classifies AS events into several types and similar to MISO, it calculates Ψ values for events and assesses the difference in these values in two conditions. VAST-TOOLS reports a value indicating the 95% confidence value for the |ΔΨ|, as a measure of significance (MV[abs(dPsi)]: The Minimum Value for |ΔΨ| at 0.95). Events having this value < 0.1 were removed in our analysis.

For individual splicing patterns of individual genes, the sashimi plots were plotted from bam files using the sashimi plot function within IGV version 2.3.97. Junction reads were obtained from the sashimi plot and plotted in Microsoft Excel.

### Clustering of expression responses

To cluster genes exhibiting correlated response patterns, we applied the WGCNA (Weighted correlation network analysis) framework^53^. The analysis was performed separately for gene expression values, NMD isoform expression values (transcripts annotated as candidates of NMD based on Ensembl annotations), NMD transcripts inclusion levels, and MISO/VAST-TOOLS Ψ values in each set of libraries treated with the same compound and the same cell line. Genes and isoforms with mean FPKM value of <1 or median value of 0 were removed. Similarly, only genes and isoforms for which the maximum FPKM value was at least 1.5 times larger than the minimum value across compound concentrations were considered; we required some minimum change in abundance values of a gene or an isoform to filter variations due to noise and random RNA-seq sampling. Inclusion levels of NMD isoforms were measured by dividing the isoform expression (FPKM value) by the corresponding gene expression, and was reported as Ψ values. Only isoforms with mean Ψ > 0.05 and median Ψ > 0.001 were kept for clustering. When clustering MISO (VAST-TOOLS) Ψ values, Ψ profiles of all event types were clustered together and no AS events were removed, apart from those filtered by MISO (VAST-TOOLS) itself. In the clustering step, the *networkType* parameter of *blockwiseModules* function was set to “signed”, the *power* parameter was set to 10 for FPKM values and 6 for Ψ values, and for all the other parameters, default values were used.

### Gene set enrichment analysis

Gene set enrichment analysis (GSEA) was carried out on a set of curated Gene Ontology (GO) terms of BP from MSigDB database^54^. The set of BPs version v.5.2 was downloaded which contains ~4700 Gene Ontology sets. We applied Hypergeometric test using R^55^, and *p*-values were corrected using BH (Benjamini and Hochberg) correction for multiple testing.

The EnrichmentMap plugin^29^ of Cytoscape^56,57^ was used to cluster similar BPs and provide a visual summary of the results. A *FDR* (false discovery rate) cutoff value of 0.01 and a *p*-value cutoff of 0.005 were applied to the identified enriched Gene Ontology sets, and Jaccard Coefficient was used as a measure of similarity of Gene Ontology sets with a cutoff value of 0.25. Names assigned to clusters were manually curated based on the set of BPs in clusters.

Odds ratios (in Fig. 5) were calculated by dividing the percentage of the genes in a given GO set that are identified by the percentage of all the genes that are identified. To compute the final scores, odd ratio scores are multiplied by −1 only if the enriched BP corresponds to the set of decreasing response patterns.

### Motif analysis

We searched for known motifs of RNA-binding proteins (RBPs) in different regions of genes that undergo alternative splicing regulation through inhibiting eIF4A3, based on the identified retained intron and skipped exon events with monotonic Ψ response profiles of MISO and VAST-TOOLS analysis. MISO and VAST-TOOLS data bases were merged and repetitive events were removed.

For retained intron and skipped exon events constituting two isoforms, the isoforms were grouped into several regions. For skipped exon events we consider seven regions: 5′ exon, skipped exon, 3′ exon, 300 nucleotides from the start/end of the 5′ intron, and 300 nucleotides from the start/end of the 3′ intron. Similarly, four regions are investigated for retained intron events: 5′ exon, 3′ exon, and 300 nucleotides from the start/end of the retained intron.

Positive samples in our analysis comprise all events with monotonic response profiles in MISO or VAST-TOOLS in any of the 4 active compound:cell line data sets. For the motif enrichment analysis, these evens were divided into two groups: monotonically increasing and monotonically decreasing ones, as the mechanism of regulation could be different.

The set of all events with monotonically increasing (decreasing) Ψ profiles were compared to the set of background MISO/VAST-TOOLS events not identified in any of the 4 active compound:cell line data sets to determine the enrichment or depletion of RNA motifs with known RBPs. Positive and negative samples were split into bins of similar lengths and for each positive sample, 10 negative samples from the same bin were selected.

PWMs for binding motifs of RBPs were downloaded from CIS-BP RNA data base^25^. First, the background frequencies of A/C/G/U nucleotides were determined. Next, for a given motif and a candidate sequence, the log odds score of that sequence being generated randomly based on background nucleotide frequencies was compared to it being generated based on PWM weights. Then, the maximum value for these log odds scores for each motif was computed and all the sequences with scores above 80% of the maximum score were counted as hits for the corresponding motif.

All motifs sharing a same RBP were clustered together and their hits were merged. Finally, hit counts were normalized by regions’ lengths, Mann–Whitney *U* test was applied to compare hit counts in regions of positive events compared to hit counts in background events; *p*-values were calculated and corrected using BH multiple test correction method. Only motifs for which the frequency of hits in positive regions were at least 25% higher or at least 25% lower than background regions were considered.

### Frameshift analysis

We investigated the potential of the identified retained intron and skipped exon events to induce frameshifts. For the VAST-TOOLS and MISO clustering results of active compounds, events with monotonic Ψ response profiles were grouped together (2 groups, increasing and decreasing). Considering that two isoforms are reported in MISO/VAST-TOOLS annotation files for each event, if the length of a retained intron or an skipped exon is not a multiple of 3, the event can induce a frameshift based on the local evidence. For the union of events with increasing (decreasing) response profiles in MISO and VAST-TOOLS, a control set was formed by all the events in MISO/VAST-TOOLS knowledge bases other than those in the set, and the ratios of events that can cause frameshifts were compared between the two sets using one-tailed Fisher’s exact test.

### Stress granule induction and detection

Cells were treated with eutomer or distomer (10 μM) alone or were further treated with vehicle alone or with sodium arsenite (NaAsO_2_, 0.1 mM) for 1 h. Stress treatments were applied either at the beginning or prior to the end of drug treatments. Western blotting was conducted using standard procedures, as previously described^58^. In brief, HeLa cells were first lysed using Nonidet P-40 lysis buffer. Then, nuclei and cell debris were cleared by centrifugation at 13,000 rpm × 20 min at 4 °C. Protein quantification was conducted with subsequent Western blotting (see Supplementary Data 15 for supporting blots).

For stress granule induction, Hela cells were treated with eutomer or distomer (10 μM, 24 h) alone or were further treated with vehicle alone or with sodium arsenite (NaAsO_2_, 0.5 mM) for 1 h prior to end of drug treatments. Stress granules detection and quantification were conducted as previously described^43^, with at least three stress granules per cell were required to consider positive^59^.

### Tubulin polymerization assay

The compounds (T-595, T-598 and cochicine control) were screened at varying concentrations with 2.0 mg ml^−1^ tubulin in Polymerization Buffer in 80 mM PIPES buffer pH 6.9, 2 mM MgCl_2_, 0.5 mM EGTA, 1 mM GTP, 10 μM DAPI, 15% v/v glycerol, and 2% v/v DMSO from the compound solvent by the manufacturer (Cytoskeleton Inc, CO, USA). Polymerization was followed by fluorescence enhancement due to the incorporation of a fluorescent reporter into microtubules as polymerization occurs. The *V*_max_ (maximum slope) values for the tubulin polymerization curves were compared against the control to create a percent of control value, which were plotted against Log_10_ each concentration. Curves were fit by non-linear regression to a 4-parameter sigmoidal dose-response equation to determine the IC_50_ for each compound.

### Illustrations

The illustrations in Fig. 1 was done by A.M. Each element was created using the software (Adobe Illustrator) and no previously-created elements were used.

### Reporting Summary

Further information on experimental design is available in the Nature Research Reporting Summary linked to this article.

## Supplementary information

Supplementary Information

Description of Additional Supplementary Items

Reporting Summary

Supplementary Data 1

Supplementary Data 2

Supplementary Data 3

Supplementary Data 4

Supplementary Data 5

Supplementary Data 6

Supplementary Data 7

Supplementary Data 8

Supplementary Data 9

Supplementary Data 10

Supplementary Data 11

Supplementary Data 12

Supplementary Data 13

Supplementary Data 14

Supplementary Data 15

Supplementary Data 16

## Data Availability

Raw sequence reads used in this study are available at the Short Read Archive (SRA) under the identifier SRP117312 and the BioProject identifier PRJNA401938. The supporting data for the main Figures can be found in the Supplementary Data. Figure 1 (Supplementary Data 2 and 3). Figure 2 (Supplementary Data 5). Figure 3 (Supplementary Data 6 and 7). Figure 4 (Supplementary Data 8). Figure 5 (Supplementary Data 9–11). Figure 6 (Supplementary Data 13 and 14). Figure 7 (Supplementary Data 15 and 16). All other data are available from the authors upon reasonable request.

## Code availability

All the complementary codes written in python and R are uploaded to a github repository at https://github.com/BCCRCMO/EIF4A3public and are available upon reasonable request.
