# Supplementary material for: Pharmacological systems analysis defines EIF4A3 functions in cell-cycle and RNA stress granule formation
Source: Commun Biol. 2019 May 3;2:165. doi: 10.1038/s42003-019-0391-9 (PMC6499833; doi:10.1038/s42003-019-0391-9)
Supplement: Supplementary file 1 — Supplementary Information [file 42003_2019_391_MOESM1_ESM.pdf]

Supplementary Information

Mazloomian et al

Supplementary Notes

1 EIF4A3 dependent transcripts identified by graded inhibition

We[1] and others have used graded short duration pharmacological modulation of gene function with novel small molecule inhibitors to identify transcripts and alternative isoforms influenced by spliceosome components. For this purpose we contrasted two specific and active allosteric EIF4A3 inhibitors (T-595, T-202) that have similar scaffolds, with an inactive but chemically identical stereoisomer compound (T-598) as a control. We have recently described the structures and synthesis of these compounds: T-595 ((3S)-4-(4-Bromobenzoyl)-3-(4-chlorophenyl)piperazin-1-yl)(6-bromopyrazolo[1,5-a]pyridin-3-yl)methanone], compound 52a [2] T-595, compound 52b T-598) and T-202 a related scaffold (3-(4-(((3S)-4-(4-Bromobenzoyl)-3-(4-chlorophenyl)piperazin-1-yl)carbonyl)-5-methyl-1H-pyrazol-1-yl)benzonitrile, compound 53a [2]). It is noteworthy that the allosteric inhibitors used in this study bind locations away from the ATPase site and is consistent with that finding that deletion/mutation of RNA-dependent ATPase function of EIF4A3 did not effect NMD or EJC formation [3] but studies involving deletion clearly do [4, 5], in support that the allosteric effect of these drugs go beyond mere inhibition of the ATPase function of EIF4A3 [6]. Both T-595 and T-202 are potent and specific (selectivity over EIF4A1,2 [2, 7]; Supplementary Data 1) allosteric eutomer inhibitors of EIF4A3 activity in helicase unwinding assays and have been demonstrated to suppress nonsense mediated decay (NMD) in reporter assays[6].

We collected RNA-seq reads from cells treated with (Methods, Supplementary Table 1) increasing concentrations of short duration exposure (6 hours, as in [1]) to each of the three compounds, from two mammalian cell lines HCT116 and HeLa, generating 32 sequencing libraries. RNA-seq reads were aligned and quantified as previously described (Methods & Figure 1a). We first examined EIF4A3 trancriptional responses, employing weighted correlation network analysis (WGCNA)[8] across control and increasing doses of active and control compounds, for normalized RNA expression (Methods) as we have demonstrated for other splicing factors[1]. Consistent with the chemical and pharmacological potency of the compounds, treatment of cells with both active eutomer allosteric inhibitors T-595 and T-202 resulted in a large number of monotonically increasing (T-595 n=1244, T-202 n=1417) or decreasing (T-595 n=1885, T-202 n=1341) transcripts (Figure 1a) in response to increasing inhibition, whereas treatment with the inactive distomer T-598 gave much smaller clusters of transcripts that showed no clear monotonic dose relationship. A high dynamic range of FPKM values was observed for monotonically increasing gene transcripts (0-5907), and monotonically decreasing genes (0-7958), across all compound:cell line pairs (Supplementary Figure 2).

Across two cell lines and the two active eutomers, monotonically increasing transcripts (n=4538) dominate over monotonically decreasing (n=2582) (Figure 1b, Supplementary Data 3) and in both cases a large proportion (∼42% and ∼47%) of monotonic responses were shared in common between two or more conditions across cell lines and compounds, indicating a high degree of conservation in EIF4A3 dependent transcriptional responses.

2 Identification of EIF4A3 dependent NMD isoforms

The role of EIF4A3 in exon-junction complex activity and its involvement in NMD prompted us to ask whether we could measure a global increase in NMD prone transcripts when EIF4A3 is chemically inhibited.

To address this, we utilised Ensembl annotations[9] for NMD prone transcript isoforms and re-examined the inhibition-response relationship using WGCNA. A large number of monotonically increasing NMD prone transcripts were found on treatments with both the active compounds (T-595 n=1168, T-202 n=1347), whereas no clear monotonically increasing cluster was observed with treatments with the chemically identical T-598 isomer (Supplementary Figure 1a, Supplementary Data 4). Moreover, no large monotonically decreasingly clusters were observed in any of the conditions, when only NMD prone isoforms were considered, entirely consistent with the fact that under normal treatment without EIF4A3 inhibiting drugs, NMD-prone transcripts are degraded. Although isoform ratios are harder to measure accurately, we also examined the ratio of NMD expression to corresponding genes (Supplementary Figure 1b) and similarly observed the largest dominant clusters for both compounds represented monotonically increases of NMD prone isoforms over the genes, although clusters were overall smaller (T-595 n=620, T-202 n=535; Supplementary Data 4). This suggested that at least for these specific NMD isoforms the increase due to the block of NMD rather than the overall upregulation all exons of the gene. Furthermore, the majority of monotonically responsive transcripts overlapped in 2 or more conditions of absolute (Supplementary Figure 1c, n=1437/2240, 64%) and isoform ratio comparisons also overlapped to a large degree (Supplementary Figure 1d, n=609/1368, 45%). These data are consistent with the dose-dependent inhibition of EIF4A3 by the eutomers resulting in a conserved (across different cell lines) monotonic increase in NMD prone transcripts.

3 Validation of EIF4A3 alternative splicing

To extend our analysis, we also determined alternative splicing events using VAST-TOOLS[10], which has additional feature types and a different event database underlying the method. VAST-TOOLS recognizes skipped exons, micro-exons, alternative 3'/5' splice sites and is more sensitive to retained intron detection, in part due to the much larger annotation database (∼155,000 vs ∼6,000) of introns. Skipped exons are stratified into multiple groups based on the complexity of events (C1, C2, C3, S), and an alternative pipeline decides whether they should be classified as micro exons (MIC).

We independently validated the EIF4A3 inhibition alternative splicing event trends by comparison with EIF4A3 siRNA knockdown experiments. We treated HeLa cells with three siRNAs (siRNA-1, siRNA-2, and siRNA-3) directed to EIF4A3 transcripts (Supplementary Figure 5, and control siRNA conditions (Supplementary Table 1). Treatment with siRNAs reduced EIF4A3 transcripts abundance by ∼92% on average (Supplementary Figure 5) and more events were detected at 48 hr vs 24 hr after siRNA knock down with 18% (MISO) and 40% (VAST) overlap between the respective time points (Supplementary Figure 6a,b). To compare these events with inhibitor associated events, we first grouped alternative splicing events in T-202 drug inhibition data into "low dose" (0.5 μM) and "high dose" (2 μM), based on the drug dose at which events were predicted and pooled siRNA reads as indicated (Supplementary Table 1 and Methods). A large proportion of alternative splicing events in the low dose group were also found in the high dose group (64% by MISO and 71% by VAST), and 42% (by MISO) and 38% (by VAST) of the events predicted in treatment:control siRNA comparison at 48 hr were also detected by drug inhibition (Supplementary Figure 6e,f). Moreover, the overlap increases with time post siRNA knock down for events detected by both MISO and VAST (Supplementary Figure 6e,f). Furthermore, the distribution of events were similar to those obtained when treated with pharmacological EIF4A3 inhibitors, T-202 and T-595 (Supplementary Figure 23b). Skipped exons, alternative last and first exons were the most abundant classes of events identified by MISO (Supplementary Figure 6c) and the most prevalent events identified by VAST in the siRNA dataset were S and RI (Supplementary Figure 6d). Moreover, as previously observed when EIF4A3 inhibitors were used, we were also able to demonstrate more alternative 3' splice site (A3SS/Alt3) and alternative 5' splice site (A5SS/Alt5) events were reported by VAST when compared to MISO.

4 Features of EIF4A3 dependent transcripts

The identification of distinct EIF4A3 dependent splice reactions allowed us to identify structural characteristic features of these transcripts. We compared intron and exon lengths of the set of monotonically increasing, monotonically decreasing, and background alternative splicing events (Supplementary Figure 24, 25). Exons of the identified monotonically increasing skipped exon events and the identified monotonically decreasing retained intron events are significantly longer that the background exons (Mann-Whitney U test; p-value <0.01, p-values in Supplementary Figure 24). Also, introns of both groups of monotonic responses are significantly shorter than background events in retained intron events (Mann-Whitney U test; p-value <0.01, p-values in Supplementary Figure 25).

5 Validation of EIF4A3 inhibition by studying specific cases

The pathway analysis (see Results) suggested strong conservation of several functions, with overlap to previous studies of EIF4A3 on specific transcripts, as well as global analysis using siRNA knockdown[4, 5]. First considering transcription, it has been noted that EJC components (mago, Y14 and EIF4A3) are required for sustained levels of MAPK[11] and an siRNA screen revealed that EJC components also reduced transcript and protein levels of MAPK in flies[12]. While there is only one MAP kinase in flies, there are a number of MAPK isoforms in mammals. In our dataset, *MAPK6* (ENSG00000069956) was found to be consistently downregulated in both HeLa and HCT-116 cells (Supplementary Figure 15a), whereas *MAPK7* (ENSG00000166484) was respectively found to be upregulated by the active inhibitors of EIF4A3 and not by the inactive distomers (Supplementary Figure 15b). We could find no other dose dependent response of any other *MAPK* transcript in our studies consistent between both eutomeric drugs in both HCT-116 (Supplementary Figure 26) and HeLa (Supplementary Figure 27) cell lines.

Among the overlapping cell cycle/apoptosis and RNA processing genes (Supplementary Data 12) identified by gene ontology term analysis above we found several examples of previously described EIF4A3 dependent splicing events, *AURKB*[13] and *BCL2L1*[13, 14] and *SRSF2*. For example, *AURKB* (ENSG00000178999) was reported to undergo specific splicing patterns on knock down of *Y14* and also *EIF4A3* in mammalian cells[14] and appeared in overlaps of our studies with external datasets[5] (see previous section). We found that 3 out of 4 of these previously described splicing events on EJC knockdown were also found when HCT-116 cells were exposed to increasing doses of T-595 but not T-598 (Supplementary Figure 19). Here a dose dependent effect was observed on alternative splice selection (Supplementary Figure 19b), skipped exon (Supplementary Figure 19c), and on the retained intron events (Supplementary Figure 19d,e) by the active eutomer, T-595, but not the inactive distomer, T-598. Significantly, the dose-dependent increase of skipped exon transcripts (one of them is annotated as an NMD transcript), illustrates that case where alternative splicing may produce a previously annotated NMD transcript; this is similar to the situation with *SRSF2*, which is involved in splicing (Supplementary Figure 28).

However, alternatively splicing due to EIF4A3 inhibition may also select for particular biologically relevant splice form as in the case of the *BCL2L1* locus (Supplementary Figure 20a) where the longer alternatively spliced transcript encodes for the anti-apoptoic $BCL - X_L$ and the shorter transcript encodes $BCL - X_S$ which is involved in promoting apoptosis. In our dataset, we observed the dose dependent alternative splicing favouring the generation of the $BCL - X_S$ transcript (Supplementary Figure 20b,c) when EIF4A3 in inhibited by the eutomer, T-595 but not the control distomer, T-598.

6 Validation of EIF4A3 inhibition on a global level

There have been several studies using whole genome approaches to study the effect of knocking down components of the EJC [4, 5]. While the actual genes that are altered in response to the knock down of Eif4a3/EIF4A3 are likely to be different due to the different expression profiles of mouse embryos and human cell lines, we could see similar proportions of events when the mouse and human datasets were similarly

analysed (ie by MISO) and selected event proportions compared with published data (Supplementary Figure 23a). We were able to compare in more depth the dataset from [5] where they used siRNA to knock down *EIF4A3* in mammalian cells. As shown in Supplementary Figure 16a-d, we compared the genes that exhibited monotonic relationship (a,c) and were detected (b,d) when HeLa and HCT-116 were treated with T-202 and T-595 but not T-598 with published data analysed by MISO (a,b) or DiffSplice (c,d) of *EIF4A3* knockdown by siRNA in HeLa [5]. In particular, we note that *SRSF2* was common in all four comparisons while *AURKB* showed up in 3 out of the four above comparisons. To compare between the different methods (MISO, VAST-TOOLS and DiffSplice) we annotated each event and summed events by genes and compared only the human genes in Wang et al and our studies using the three different methods (Supplementary Figure 16e). As expected, we see a large overlap between the two methods used within each of the two of the studies (57% (46/80 genes overlap within Wang et al and 53% (149/281) of the smaller dataset were common within our study). Significantly, 64 genes were common in at least 3 different experiments over the two separate studies and *AURKB* was among these 64 genes while 7 genes appeared common in all the four comparison (including *SRSF2*). These comparisons validate our studies that the EIF4A3 inhibitors modulate genes in common with previously published works as well as our own siRNA experiments. The genes *SRSF2* and *AURKB* found in most of the datasets compared are also involved in the processes we observe to be perturbed by EIF4A3 inhibiting drugs, namely splicing and chromosome segregation (Supplementary Data 12), appear to be consistent with the phenotypes observed on treatment with the active eutomeric drugs.

7 Rational for studying EIF4A3 in stress granule formation

Among the pathways and functions identified as statistically significant, besides those involved in RNA splicing and cell cycle regulation which we had addressed in the previous sections, we noticed several biological processes such as protein and RNA localization, as well as stress response pathways, where EIF4A3 has not been previously described to be involved in. Coupled with the known and demonstrated role of EIF4A3 in NMD, we hypothesised that EIF4A3 might have a previously unexplored role in the cellular stress response pathway. Moreover, the inhibition of the enzymatic activity of EIF4A3 by the inhibitors leads to an increase of NMD transcripts, we wanted to observe net effect of this on stress granules in cells as it is known that mRNA that is not undergoing active translation aggregate (such as accumulated NMD transcripts) into transient stress granules.

8 Summary of the findings on stress granules

When we applied the eutomeric inhibitors of EIF4A3 to cells, we found that this actually inhibited stress granule formation as assayed by two key SG anchor proteins, G3BP1 and TIA1 (Figure 7). Further analyses revealed that a large proportion of the known genes involved in stress granule formation are themselves regulated by EIF4A3 inhibition on one or more of the following mechanisms assayed (gene expression, NMD transcript stabilization, differential splicing) (Supplementary Table 6). We demonstrated that inhibition of EIF4A3 not only reduced the steady-state levels of stress granule modulators (G3BP1 and TIA1), but that it also prevented stress granule formation in the presence of an arsenite inducer (Figure 7b). Finally, we showed that inhibition of EIF4A3 could significantly reduce the protein levels of key stress granule modulators (G3BP1 and TIA1) in cells subjected to early treatment with stress granule inducer, arsenite, potentially suggesting a role of EIF4A3 in the maintenance of stress granules.

9 Rational for study design and EIF4A3 inhibitors

We have previously shown[1] that specific small molecule inhibitors can be used in graded inhibition experiments to establish which splicing and transcription states exhibit a strict monotonic dose relationship, thus defining the core set of dependent features. Using this approach with EIF4A3 has not proven simple to date,

as previously available inhibitors such as hippuristanol[15] also inhibit EIF4A1 and EIF4A2, which have different roles in trancription-translation control. Eutomers used in this study bind EIF4A3 and allosterically inhibit its helicase activity[7] leading to a reduction in NMD activity measured by reporter plasmids[6], as would be anticipated given the known functions of EIF4A3.

10 Discussion of contrasts between our and published examples

Our data supports the conservation of core EIF4A3 transcriptional responses across species, but with some differences in detail. For example, MAPK was found to be regulated by EIF4A3 and EJC components in Drosophila[11, 12], *Mapk13* in mouse and *MAPK6,7* (Supplementary Figure 15) and *MAPK13* (Supplementary Figure 29) in our studies. It should be noted that while there appears to be conservation of these processes involving similar genes regulated by EJC and EIF4A3, the mechanism of EJC involvement in NMD[16]), from Drosophila[17] to human, is distinct.

Furthermore, while EIF4A3 and EJC disruption in Drosophila affects genes with long introns[18], our data shows that exons of EIF4A3-dependent splicing are significantly longer than background exons in monotonic both RI and SE events (Supplementary Figures 24, 25), only the introns of montonically decreasing SE events are longer (Supplementary Figure 24) while RI events with a monotonic relationship with EIF4A3 inhibition appear to be shorter (Supplementary Figure 25). Nonetheless, global splice class proportions (Supplementary Figure 23) and overlap of genes between our study and previous studies suggest that inhibition of EIF4A3 by the eutomeric drugs in this study regulate a subset of genes shown to be deregulated by knockdown and depletion[4, 5] (Supplementary Figure 16).

11 Specific examples of splicing and NMD

Only a subset of the transcripts in the monotonically active-inhibitor responsive clusters could be predicted to produce pre-mature stop codons by generation of transcripts that are not multiples of three and hence cause frameshifts, assuming the same start frame (Supplementary Table 4). While the data does not rule out the possibility of NMD isoforms complicating the alternative splicing analysis, it clearly demonstrated that while some alternative splicing events may produce NMD transcripts (which are stabilized by inhibition of EIF4A3 and hence the EJC function) such as that of *SRSF2* (Supplementary Figure 28) and *AURKB* (Supplementary Figure 19), not all alternative splicing events (e.g. *BCL-X*, Supplementary Figure 20) lead to NMD transcripts that are stabilized by EIF4A3 (and EJC) inhibition[20].

12 Discussion on complexities of EIF4A3 regulation

The complexity of EIF4A3-RBP regulation extends to RBPs that have both their NMD transcripts and NMD isoforms regulated monotonically by EIF4A3 (e.g. SRSF2 (Supplementary Figure 28),3,6 (Supplementary Figure 22), RBMX, PSPC1, RBM4B). Many of these RBPs which show differences in binding patterns in the monotonically increasing (e.g. SRSF2,3,6, PPRC1, RBMX, PTBP2) or decreasing (e.g. IGF2BP3, TARDBP, ZFP36, CPEB4, SRSF4, MBNL3) transcripts are themselves modulated by EIF4A3. However, using the method of graded inhibition and focusing on monotonically regulated transcripts was able to define the co-regulatory RBP features embedded in EIF4A3 regulated transcripts.

13 Significance of features of EIF4A3 dependent splicing events

Investigation of hard-coded sequence features of EIF4A3-dependent alternatively spliced events demonstrated that exons of transcripts that undergo EIF4A3-dependent alternative splicing, particularly retained intron events and skipped exon events, are longer than the average background (non-EIF4A3 dependent) exons and introns (particularly those in monotonic retained intron events) appear to be shorter the background introns

(Supplementary Figures 24, 25). We also observed notable variation between alternative last exon selection in the set of monotonically increasing and decreasing events (Supplementary Figure 8). This finding suggests the involvement of EIF4A3 in the selection of alternative terminal exons of a subset of genes and inhibition of EIF4A3 shows a skewing towards the proximal terminal exons of the genes in the monotonically decreasing clusters, reminiscent of CDK12 action on terminal exons [19].

14 Comparisons of the distributions of RBPs on EIF4A3 transcripts

Considering the finding of specific disctribution of RMP motifs on EIF4A3 transcripts, it appears that the motifs of RBPs (e.g. RBM8A/Y14, RBM4B, SRSF4,6, RBM1,2, RBM46,47) are depleted in the 5' intronic region of monotonically increasing skipped exon transcripts are inversely correlated in monotonically decreasing skipped exon transcripts. This mirroring of RBP motifs in two opposing effects are consistent with known mechanisms of splicing and have been previously observed for CLK2[1]. However, unlike CLK2 in which motif pattern changes are observed in both upstream and downstream introns and exons[1], EIF4A3 inhibition results in motif pattern changes predominately in the 5' intronic regions. Consistent with this, Y14/RBM8A, a core component of EJC which functionally cooperates with EIF4A3 has enriched binding motifs in both 5' exons and 5' introns of monotonically increasing transcripts (Figure 4), in close vicinity of the splice junction[21], in agreement with Y14's known functional roles[22].

15 Discussion on Y14 RBP motifs on EIF4A3 transcripts

Although previous structural studies concluded that the Y14 RBP motif is involved in MAGOH binding and unavailble for binding RNA as part of the EJC core complex [23], nevertheless from Y14 binding experiments [24, 25], and direct RNA *in vitro* binding experiments[26], associated motifs have been identified 5âĂŹ of splice junctions. According to Ray et al (2013) [26], using RNAcompete technology, the affinity purification of the RRM_1 (residues $54-124$) of human Y14/RBM8A yielded bound RNA oligos with a consensus motif of "RYGCGCB". This was determined experimentally. (see $http : //hugheslab.ccbr.utoronto.ca/supplementary - data/RNAcompete\_eukarya/Experiment\_reports/RNCMPT00056/RNCMPT00056_report.html$ accessed 15 Jan 2019). We found that the consensus sequence is enriched only in monotonically increasing spliced transcripts at the splice-junctions and depleted in monotonically decreasing 5' intron regions (Figure 4a). This observation would be entirely consistent with the known functions of Y14/EJC in EIF4A3-dependent alternative splicing at the 5' exon and 5' intron regions adjacent to the slice site. While Ray et al (2013) [26] found an enrichment of the motif in 5' UTR perhaps reflecting the role in mRNA cap binding process (Chuang et al 2013), here we show that the experimentally determined motif is enriched in regions adjacent to the splice junctions specifically of EIF4A3-dependent spliced transcripts (Figure 4c).

While the structural studies have concluded that the sole RRM of human Y14 (residues 95-174) binds MAGOH in the EJC and not contact RNA directly [27], our systems approach finds that Y14 *in vitro* binding motif is nevertheless enriched in certain EIF4A3 dependent transcripts. This suggests that Y14 could potentially interact with RNA in at least the subset of transcripts which are regulated by EIF4A3. If so, this would be consistent with earlier work mapping Y14 binding to mRNA [24] as well as more recent experiments demonstrating that Y14 mutant L118R binds mRNA without MAGOH [25], which shows that Y14 can indeed associate with mRNA independently of MAGOH as demonstrated by Ray et al [26]. Such a result may cause us to rethink the current mechanism of EJC formation at the slice junctions; further experimental work is needed to address these aspects.

16 Genes involved in the cell cycle phenotype observed when EIF4A3 is inhibited

When we surveyed the global cellular processes affected by transcript levels and NMD-prone isoforms on treatment with the eutomeric inhibitors of EIF4A3 (Figure 5), we observed NMD-prone transcripts of the genes involved in cell cycle processes such as spindle formation, chromosomal alignment and segregation (*CDC27* (Supplementary Figure 14), *AURKB* (Supplementary Figure 19), and the induction of apoptosis (e.g. $BCL-X_S$, Supplementary Figure 20) were affected. It is also significant that a number of genes were involved in the chromosomal passenger complex (CPC) including *AURKB* and *BIRC5* (survivin) as well as downstream components of its signalling pathway such as KIF20A and NSUN2. This implicates EIF4A3 in the maintenance of proper cell cycle regulation particularly through the processes of spindle formation, chromosomal alignment and segregation.

17 Implications in cancer biology

The implications in tumour biology and metastasis[28] are significant given the importance of stress granules in the survival of tumour cells under various stresses. This may confer a selective advantage for the cancer or clones of cancer cells that can adapt to growing under conditions of oxidative stress, oxygen or nutrient depletion, genotoxic stress or even cytotoxic therapy. Key to this survival mechanism is selective translation and an emerging mechanism is the temporary storage of mRNAs in a silenced state in these cytoplasmic granules, which comprise RNA binding proteins and silenced mRNAs, that are temporarily stored in stress granules until the stress is abated[29]. Indeed, stress granules has been shown to result in translational silencing of stress granule-associated mRNAs, but with selective translation of a subset of mRNAs that are somehow excluded from stress granules[30]. Our findings that EIF4A3 (of the EJC) is important for stress granule biology and that the splicing of TIA1, a key factor involved in stress granule formation may add a new mechanistic link.

18 Future work

Through global studies using active eutomers and an inactive distomer, and focussing on transcripts with a monotonic relationship with the drug doses of the eutomers, we identified a subset of genes which have considerable overlap with previous studies involving the inhibition of EIF4A3 and other components of the EJC. The analysis of these splicing and NMD transcripts reveal sequence-specific features of EIF4A3 regulation. Furthermore, we have identified key genes which clearly demonstrate dose-dependence to the active eutomers that are key players in these respective pathways (eg. *SRSF2* (splicing), *CDC27* (cell cycle), *AURKB* (chromosome segregation), $BCL-X_S$ (apoptosis) and *TIA1* (stress granules)). While we acknowledge that there are likely many other genes involved, these particular identified EIF4A3-responsive genes provide examples of the mechanisms by which inhibition of EIF4A3 could lead to the biological effects observed and it would be interesting to elucidate the precise pathways and the involvement of EIF4A3 in these biological processes. The availability of these small molecular inhibitors and the control distomer will prove useful in this undertaking.

Supplementary References

[1] Funnell, T. *et al.* Clk-dependent exon recognition and conjoined gene formation revealed with a novel small molecule inhibitor. *Nat Commun* **8**, 7 (2017).

[2] Ito, M. *et al.* Discovery of novel 1,4-diacylpiperazines as selective and cell-active eif4a3 inhibitors. *J Med Chem* **60**, 3335–3351 (2017).

[3] Shibuya, T., Tange, T., Stroupe, M. E. & Moore, M. J. Mutational analysis of human eIF4AIII identifies regions necessary for exon junction complex formation and nonsense-mediated mRNA decay. *RNA* **12**, 360–374 (2006).

[4] Mao, H., McMahon, J. J., Tsai, Y. H., Wang, Z. & Silver, D. L. Haploinsufficiency for Core Exon Junction Complex Components Disrupts Embryonic Neurogenesis and Causes p53-Mediated Microcephaly. *PLoS Genetics* **12**, e1006282 (2016).

[5] Wang, Z., Murigneux, V. & Le Hir, H. Transcriptome-wide modulation of splicing by the exon junction complex. *Genome biology* **15**, 551 (2014).

[6] Iwatani-Yoshihara, M. *et al.* Discovery and characterization of a eukaryotic initiation factor 4a-3-selective inhibitor that suppresses nonsense-mediated mrna decay. *ACS Chem Biol* (2017).

[7] Ito, M. *et al.* Discovery of selective atp-competitive eif4a3 inhibitors. *Bioorg Med Chem* **25**, 2200–2209 (2017).

[8] Langfelder, P. & Horvath, S. WGCNA: an R package for weighted correlation network analysis. *BMC Bioinf.* **9**, 10.1186/1471–2105–9–559 (2008).

[9] Cunningham, F. *et al.* Ensembl 2015. *Nucleic acids research* **43**, D662–D669 (2015).

[10] Irimia, M. *et al.* A highly conserved program of neuronal microexons is misregulated in autistic brains. *Cell* **159**, 1511–1523 (2014).

[11] Roignant, J. Y. & Treisman, J. E. Exon Junction Complex Subunits Are Required to Splice Drosophila MAP Kinase, a Large Heterochromatic Gene. *Cell* **143**, 238–250 (2010).

[12] Ashton-Beaucage, D. *et al.* The Exon Junction Complex Controls the Splicing of mapk and Other Long Intron-Containing Transcripts in Drosophila. *Cell* **143**, 251–262 (2010).

[13] Fukumura, K. *et al.* The Exon Junction Complex Controls the Efficient and Faithful Splicing of a Subset of Transcripts Involved in Mitotic Cell-Cycle Progression. *International journal of molecular sciences* **17**, 1153 (2016).

[14] Michelle, L. *et al.* Proteins associated with the exon junction complex also control the alternative splicing of apoptotic regulators. *Molecular and cellular biology* **32**, 954–67 (2012).

[15] Bordeleau, M.-E. *et al.* Functional characterization of ireses by an inhibitor of the rna helicase eif4a. *Nat Chem Biol* **2**, 213–20 (2006).

[16] Gatfield, D., Unterholzner, L., Ciccarelli, F. D., Bork, P. & Izaurralde, E. Nonsense-mediated mRNA decay in Drosophila: At the intersection of the yeast and mammalian pathways. *EMBO Journal* **22**, 3960–3970 (2003).

[17] Hansen, K. D. *et al.* Genome-wide identification of alternative splice forms down-regulated by nonsense-mediated mRNA decay in Drosophila. *PLoS Genetics* **5**, e1000525 (2009).

[18] Ashton-Beaucage, D. & Therrien, M. The exon junction complex a splicing factor for long intron containing transcripts? *Fly* **5**, 1–10 (2011).

[19] Tien, J. F. *et al.* CDK12 regulates alternative last exon mRNA splicing and promotes breast cancer cell invasion. *Nucleic Acids Research* **45**, 1–19 (2017).

[20] Metze, S., Herzog, V. A., Ruepp, M.-D. & Mühlemann, O. Comparison of EJC-enhanced and EJC-independent NMD in human cells reveals two partially redundant degradation pathways. *RNA (New York, N.Y.)* **19**, 1432–48 (2013).

[21] Saulière, J. *et al.* CLIP-seq of eIF4AIII reveals transcriptome-wide mapping of the human exon junction complex. TL - 19. *Nature structural & molecular biology* **19 VN - r**, 1124–1131 (2012).

[22] Nielsen, K. H. *et al.* Mechanism of ATP turnover inhibition in the EJC. *RNA (New York, N.Y.)* **15**, 67–75 (2009).

[23] Ballut, L. *et al.* The exon junction core complex is locked onto RNA by inhibition of eIF4AIII ATPase activity. *Nature Structural & Molecular Biology* **12**, 861–869 (2005).

[24] Kim, V. N. *et al.* The Y14 protein communicates to the cytoplasm the position of exon-exon junctions. *The EMBO journal* **20**, 2062–8 (2001).

[25] Kataoka, N. *et al.* Specific Y14 domains mediate its nucleo-cytoplasmic shuttling and association with spliced mRNA. *Scientific reports* **1**, 92 (2011).

[26] Ray, D. *et al.* A compendium of rna-binding motifs for decoding gene regulation. *Nature* **499**, 172–177 (2013).

[27] Lau, C.-K., Diem, M. D., Dreyfuss, G. & Van Duyne, G. D. Structure of the Y14-Magoh core of the exon junction complex. *Current biology : CB* **13**, 933–41 (2003).

[28] Somasekharan, S. P. *et al.* YB-1 regulates stress granule formation and tumor progression by translationally activating G3BP1. *The Journal of cell biology* **208**, 913–29 (2015).

[29] Anderson, P. & Kedersha, N. Stress granules. *Current Biology* **19**, R397–R398 (2009).

[30] Anderson, P., Kedersha, N. & Ivanov, P. Stress granules, p-bodies and cancer. *Biochim Biophys Acta* **1849**, 861–70 (2015).

[31] Braunschweig, U. *et al.* Widespread intron retention in mammals functionally tunes transcriptomes. *Genome research* **24**, 1774–1786 (2014).

[32] Merico, D., Isserlin, R., Stueker, O., Emili, A. & Bader, G. D. Enrichment map: a network-based method for gene-set enrichment visualization and interpretation. *PLoS One* **5**, e13984 (2010).

[33] Hu, Y. *et al.* DiffSplice: the genome-wide detection of differential splicing events with RNA-seq. *Nucleic acids research* **41**, e39–e39 (2012).

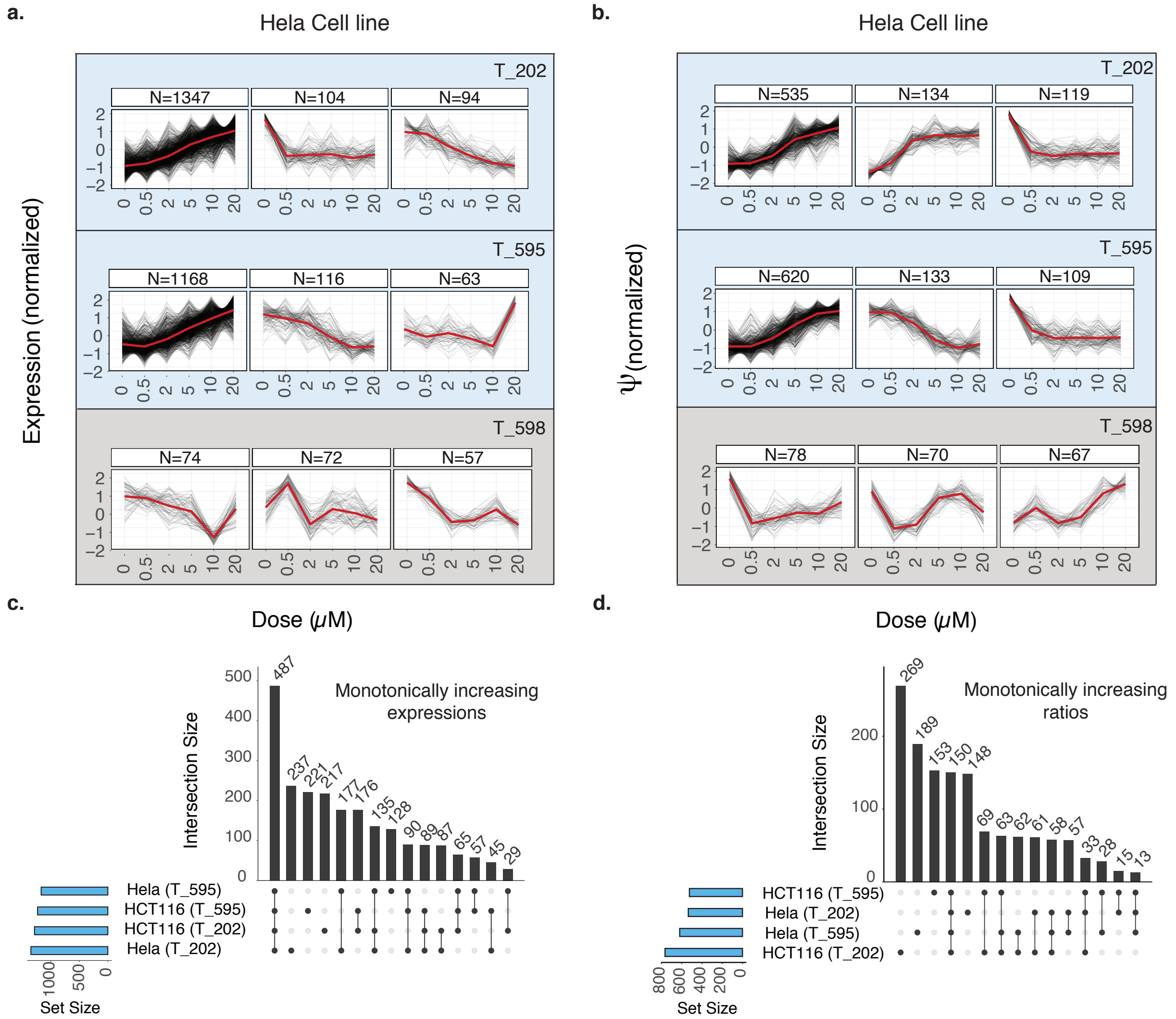

Supplementary Figure 1: Investigating NMD prone transcripts responses to graduated EIF4A3 inhibition. a. Results of clustering expression profiles for NMD prone transcripts as annotated by Ensembl. Blue and gray background colors represent the clustering of isoform expression profiles for the active compounds and the control compound, respectively (3 top clusters are shown per each compound, sorted based on cluster sizes). X and Y axes represent inhibitor concentrations, and isoform expression values normalized using upper quartile normalization method. Each black line illustrates expression response of one NMD prone isoform in a given condition, and the red lines represent the consensus response in each cluster computed by averaging expression values of all the genes. Most isoforms are over-expressed in higher inhibition levels of active compounds, in contrast to the observed responses when cells are treated by the control compound. **b.** Inclusion levels of NMD prone isoforms (Ψ values normalized by the the expression of corresponding genes) are clustered using WGCNA. Similar to part **a**, isoforms are predominantly over-expressed for active compounds shown in blue backgrounds. **c., d.** Overlap sizes among subsets of monotonically increasing profiles of **a** and **b** are shown for the two active compounds. Each bar corresponds to the set of events only detected in the cell line:inhibitor pairs determined by black circles.

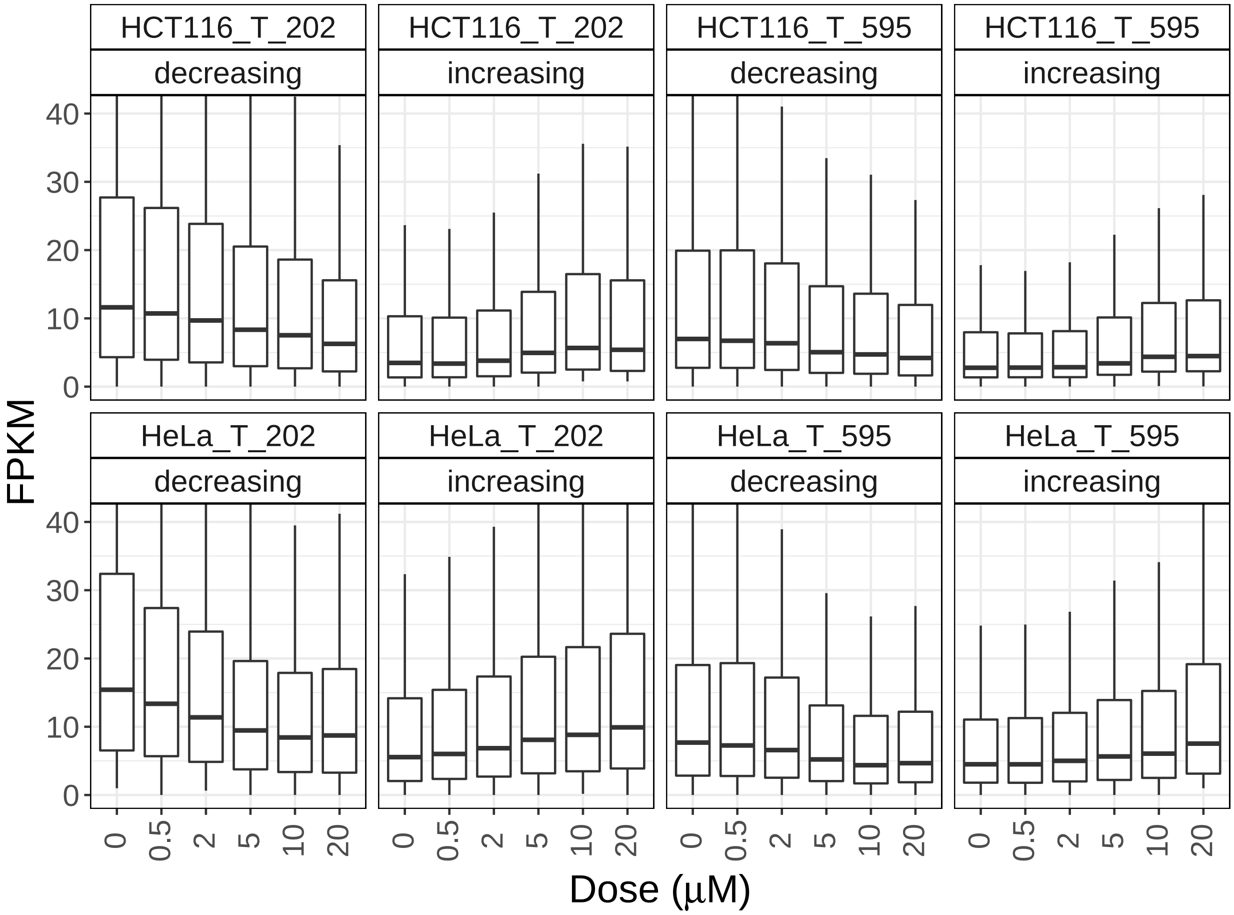

Supplementary Figure 2: Box plots showing gene expression FPKM values for the set of monotonically increasing and the set of monotonically decreasing clusters for each of the cell line:compound pairs.

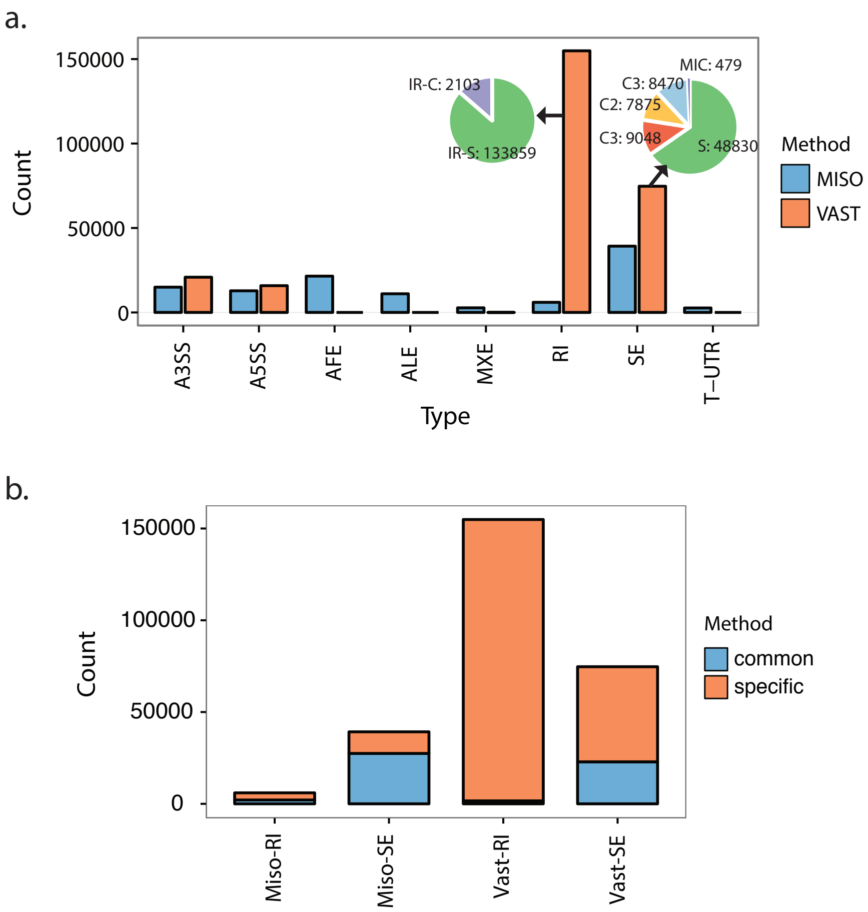

Supplementary Figure 3: A comparison of MISO and VAST-TOOLS databases. **a.** Comparing the number of events of each AS types in VAST-TOOLS and MISO. For VAST-TOOLS events intron retention (RI) events and skipped exon (SE) events are grouped into several types as explained in[10, 31]. There are many more retained introns annotated in VAST-TOOLS compared to MISO. **b.** The overlap between MISO and VAST-TOOLS databases for skipped exons and retained introns. For each type in one database (*e.g.* retained intron in MISO) we checked the number of events intersecting with at least one event annotation of the same type from the other database. Although the number of RI events in VAST-TOOLS is much larger, among annotated MISO events only 35% intersect with one or more VAST-TOOLS RI annotations.

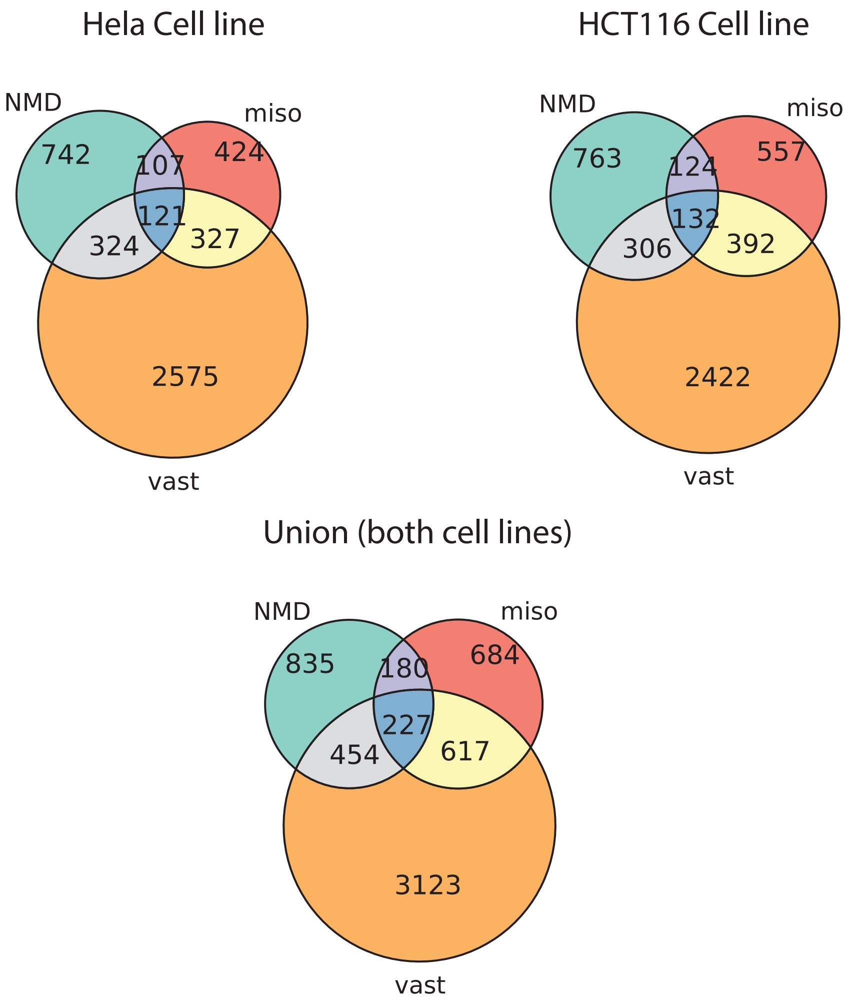

Supplementary Figure 4: A comparison of genes with NMD prone transcripts showing monotonically increasing expression, and genes with monotonically changing Ψ values for AS events identified by MISO and genes with monotonically changing Ψ values for AS events identified by VAST-TOOLS. The union of genes with identified monotonic events in at least one of the conditions in a set are shown for HeLa, HCT116, and the union of the two cell lines. Differences in MISO and VAST-TOOLS analytical approaches and their knowledge bases causes the observed variations between the corresponding sets. Additionally, not all the identified AS events induce NMD prone transcripts, and among AS events resulting in NMD prone transcripts some of the corresponding transcripts may not be annotated as NMD isoforms in Ensembl annotations, and similarly some of the NMD causing AS events may not be present in MISO or VAST-TOOLS knowledge bases.

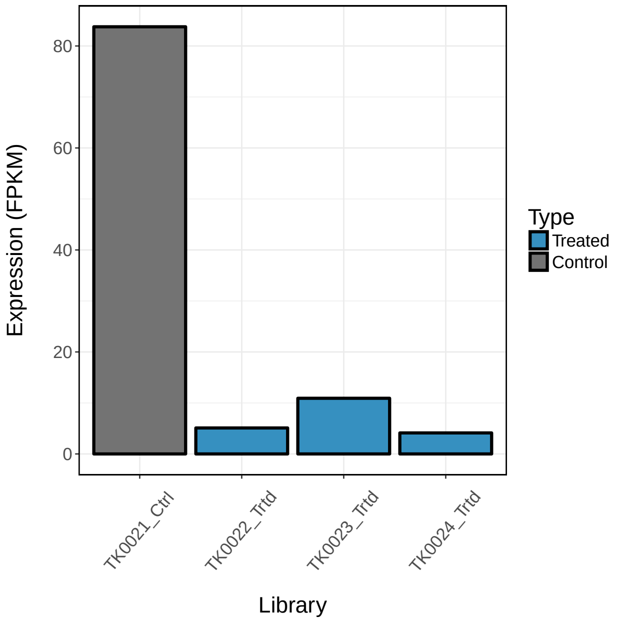

Supplementary Figure 5: Transcript abundance of *EIF4A3* upon knocking down the gene using siRNAs. As shown with blue colors, siRNAs directed to *EIF4A3* significantly reduces transcript abundance of *EIF4A3*.

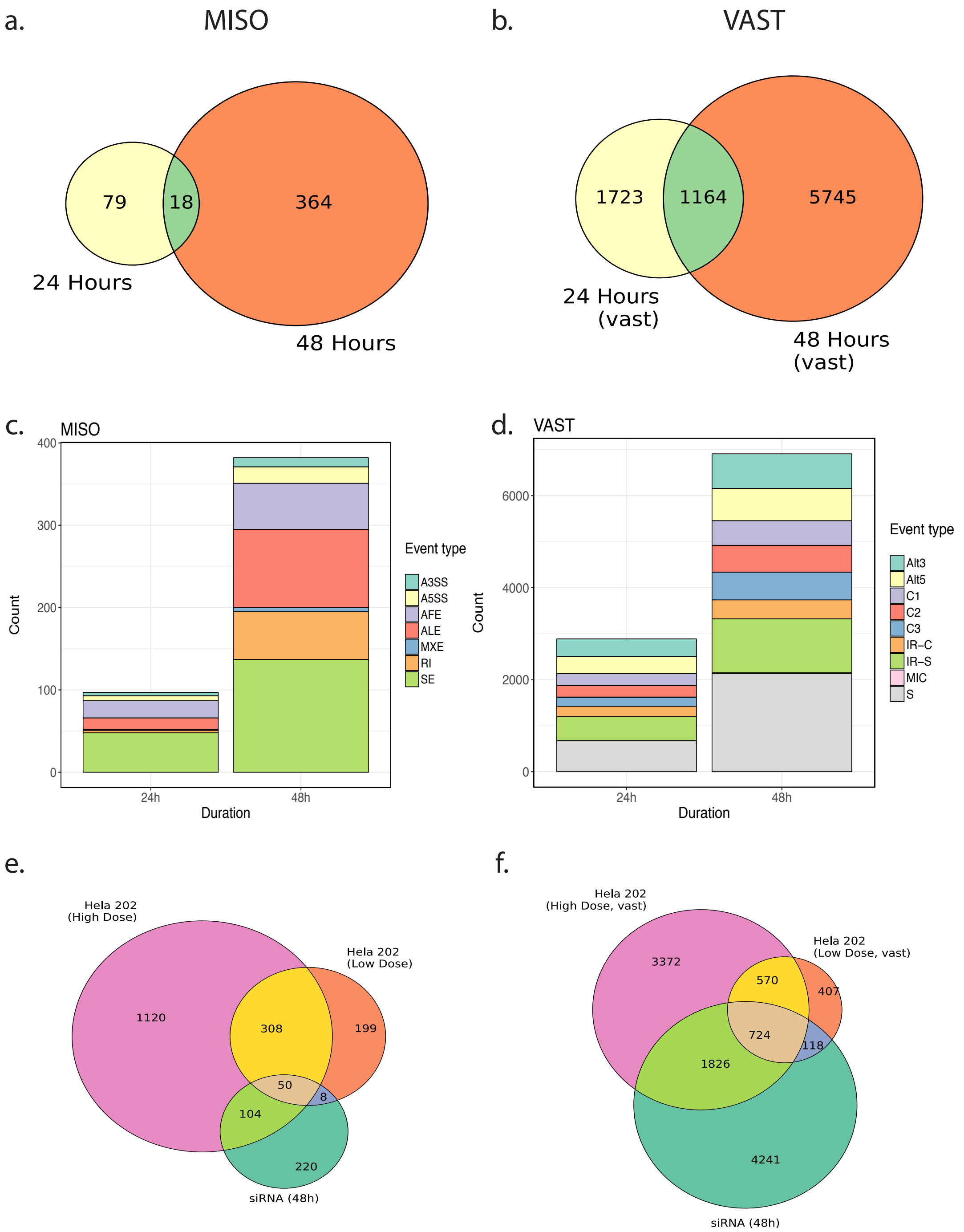

Supplementary Figure 6: Comparison of splicing events on treatment between drug-treated, T-202 (high and low dose, 6 hr treatment) and siRNA directed to *EIF4A3* (24 hr and 48 hr treatment) in HeLa cells. Venn diagram showing the overlap of of splicing events after 24 and 48 hr treatment as determined by **a.** MISO and **b.** VAST. Types of splicing events as identified at the various time points after siRNA knock down of EIF4A3 as determined by **c.** MISO and **d.** VAST. Overlap of events when HeLa were treated with high or low doses of T-202 and siRNA directed to EIF4A3 after 48 hr as determined by **e.** MISO and **f.** VAST.

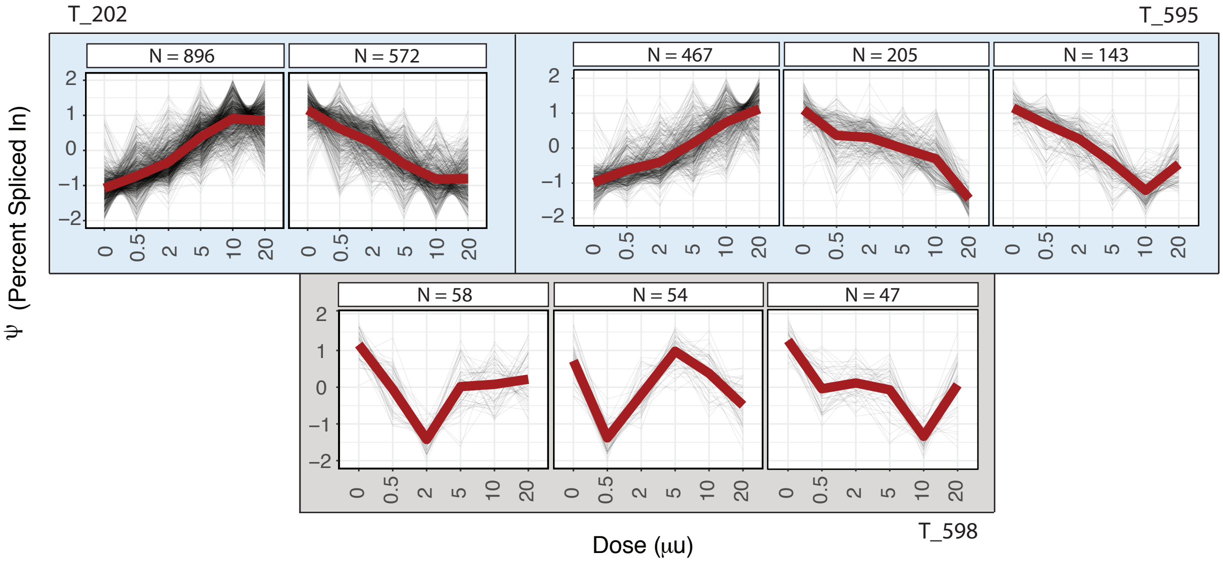

Supplementary Figure 7: Clustering Ψ response profiles of alternatively spliced isoforms for HCT116 cell line using WGCNA. The inclusion levels (Ψ values) of alternatively spliced isoforms calculated by MISO are clustered using WGCNA. Background colors represent the clustering of Ψ profiles for the active (blue) compounds and the control (grey) compound, respectively (at most 3 clusters are shown per each compound, sorted based on cluster sizes). X and Y axes represent inhibitor concentrations, and MISO Ψ values. Each black line illustrates Ψ response of one MISO isoform in a given condition, and the red lines represent the consensus response in each cluster computed by averaging Ψ values of all the isoforms.

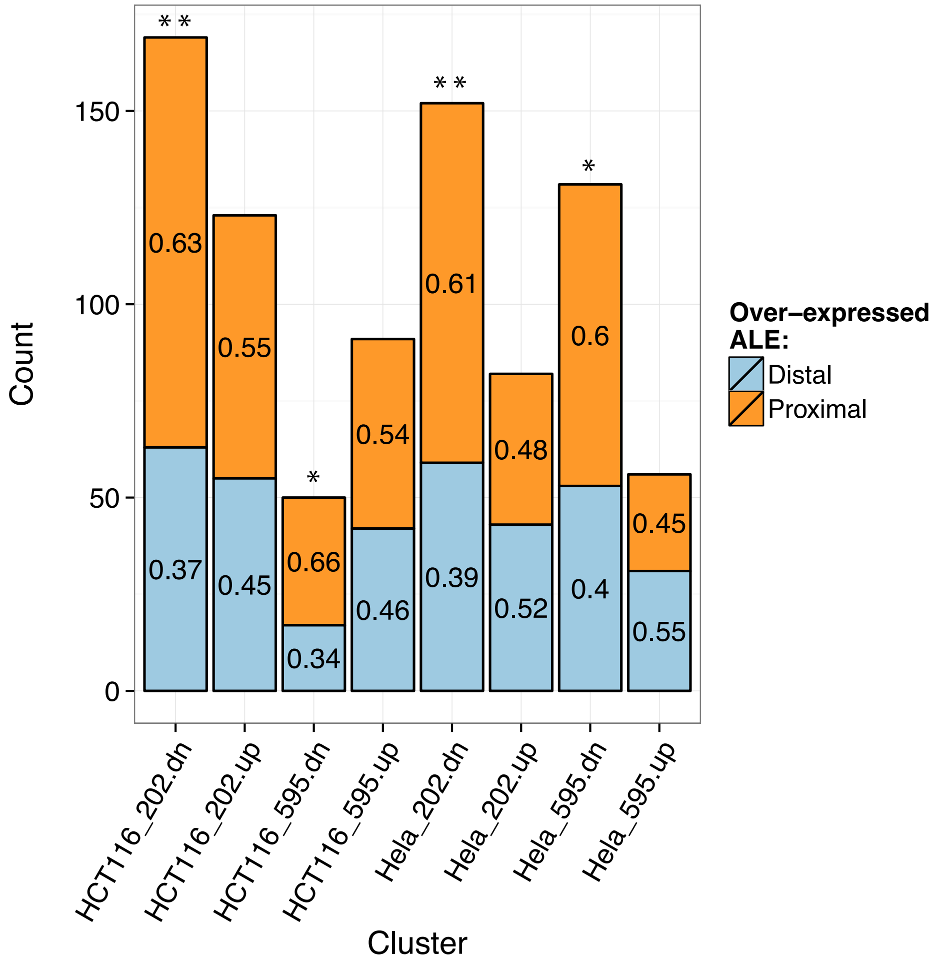

Supplementary Figure 8: The abundance of alternative last exon (ALE) events favouring the proximal (5') vs the distal (3') last exon. For the set of clustered events with monotonic responses in the active compound libraries, we extracted ALE events and counted the number of those that over-express the proximal exon upon EIF4A3 inhibition, and those that over-express the distal exon. As illustrated, for the set of monotonically decreasing clusters (.dn), events are slightly but significantly biased towards those that over-express the proximal exon (*: p-value <0.05, **: p-value <0.01, p-values calculated using Chi-square test: HCT116_202.dn: 0.0009, HCT116_202.up: 0.2411, HCT116_595.dn: 0.02365, HCT116_595.up: 0.4631, Hela_202.dn: 0.0058, Hela_202.up: 0.6587, Hela_595.dn: 0.02894, Hela_595.up: 0.4227.)

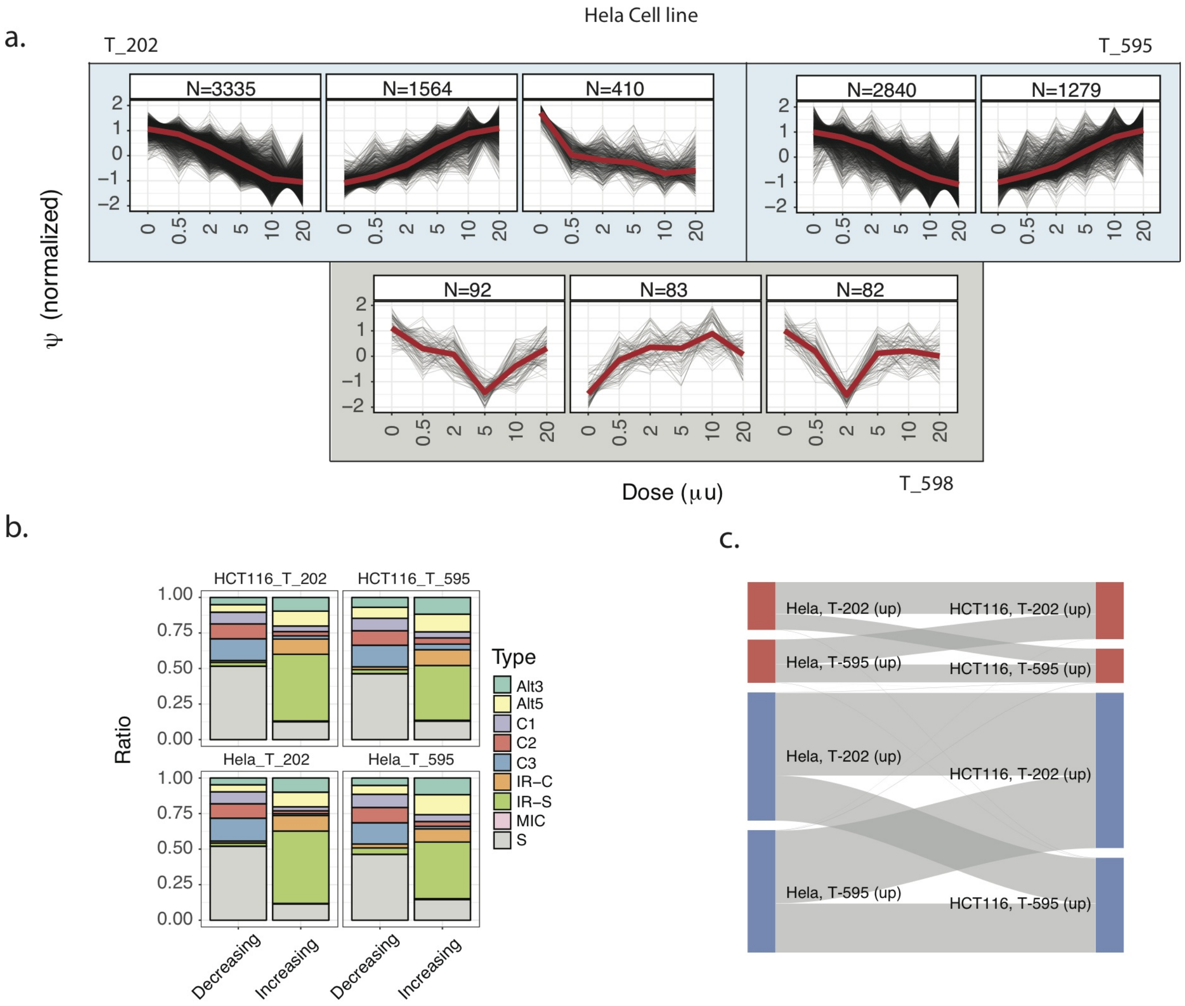

Supplementary Figure 9: Clustering Ψ response profiles of AS isoforms using WGCNA. **a.** The inclusion levels (Ψ values) of AS isoforms calculated by VASTtools are clustered using WGCNA. Blue and grey background colors represent the clustering of Ψ profiles for the active compounds and the control compound, respectively (at most 3 clusters are shown per each compound, sorted based on cluster sizes). X and Y axes represent inhibitor concentrations, and VAST-TOOLS Ψ values. Each black line illustrates Ψ response of one VAST-TOOLS isoform in a given condition, and the red lines represent the consensus response in each cluster computed by averaging Ψ values of all the isoforms. **b.** Stacked bars, showing the ratio of each AS type in the set of monotonically increasing and decreasing events. The composition of AS types is clearly different between the two sets similar to what we observed in MISO results shown in the main text. **c.** A sankey diagram illustrating the proportion of shared events between monotonic sets identified in part **a**. Monotonically increasing and decreasing sets are shown in red and blue rectangles, respectively. The direction of changes of events highly agree between cell lines and between drugs.

Hela cell line

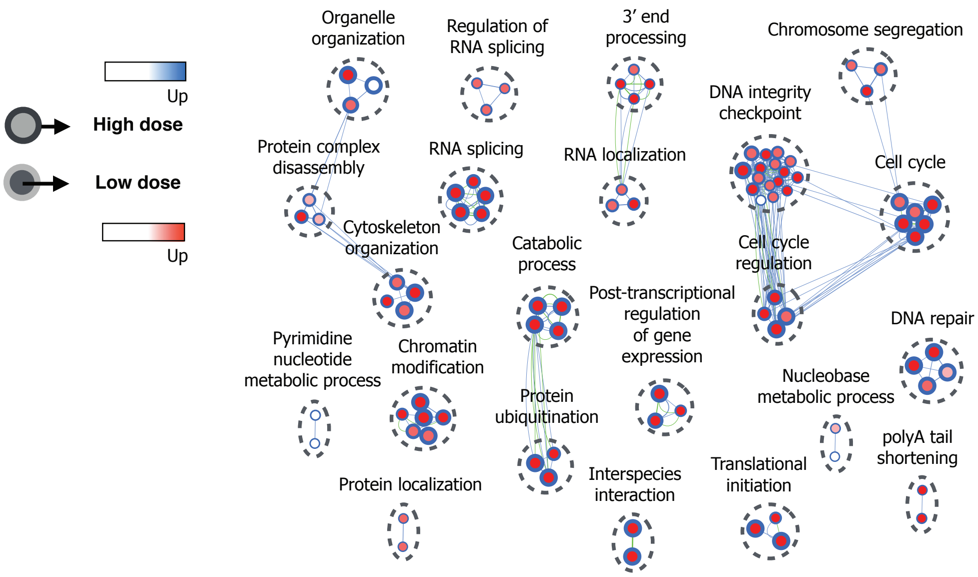

HCT116 cell line

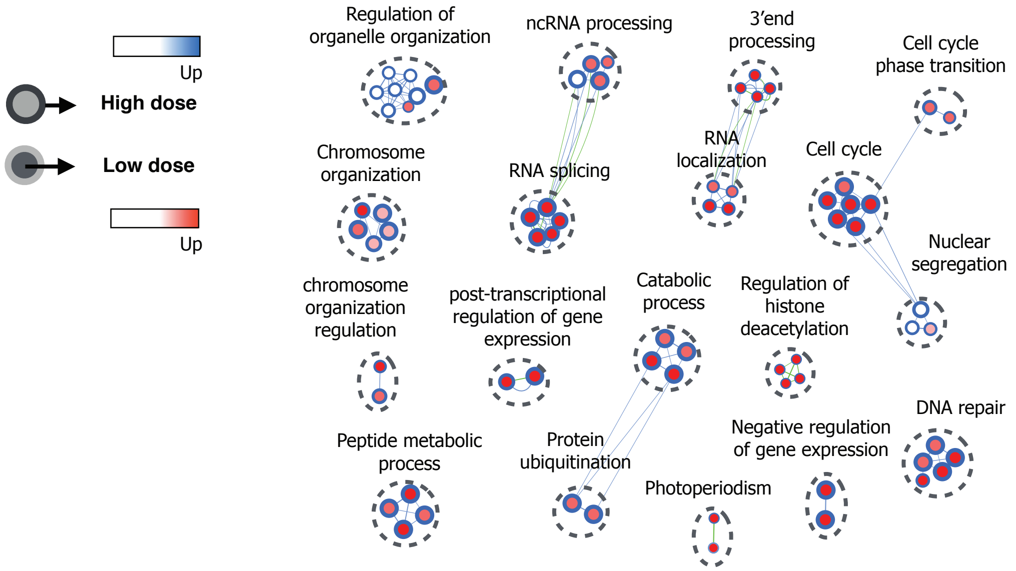

Supplementary Figure 10: Biological processes enrichment analysis for genes undergoing alternative splicing upon EIF4A3 inhibition based on MISO analysis. The identified MISO alternative splicing events were classified into “low dose” and “High dose” as explained. For the set of genes undergoing alternative splicing regulation for each group and each cell line (results were merged for the two compounds), we performed biological process enrichment analysis. Each node represents a biological process with genes over-represented in the set of events detected in high concentration treatments (blue, node rings) or low concentration treatments (red, node cores). Biological processes are clustered using EnrichmentMap[32] based on the overlap of their monotonic genes.

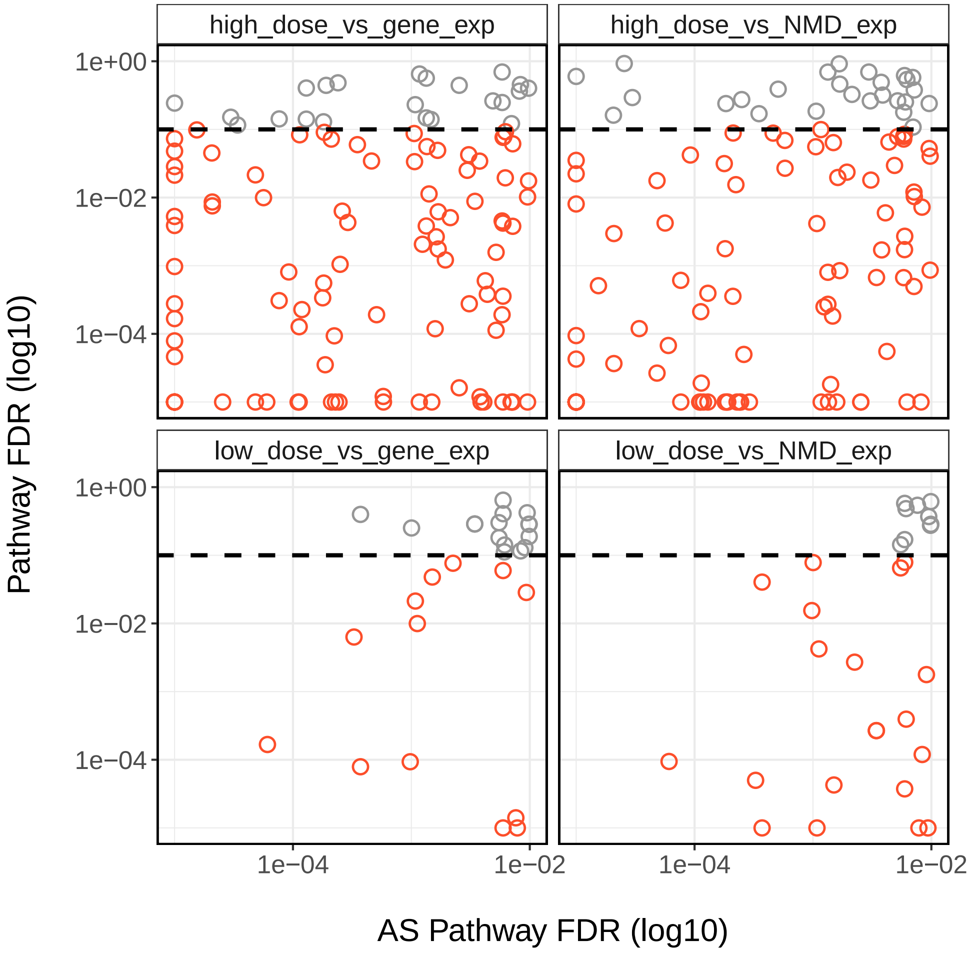

Supplementary Figure 11: Pathways enriched by AS regulated genes are also regulated through gene expression and NMD. For pathways detected to be enriched by AS regulated genes with FDR <0.01 (low drug concentrations and high drug concentrations, separately), the FDR values are plotted against the corresponding FDR values for gene expression pathway analysis (for monotonic genes) and NMD pathway analysis (monotonically increasing transcripts). For each pathway, the minimum FDR value among the 4 active compound:cell line libraries was selected and values smaller than 10^{-5} were replaced by 10^{-5}. The dashed line is plotted at the FDR value of 10^{-1} for the Y axis. Among pathways detected for high drug concentrations 81% (94/116) and 78% (90/116) of them are identified (with FDR <0.1, red circles) for the set of genes with monotonic responses and the set of NMD transcripts with monotonically increasing responses. Moreover, among pathways detected for low drug concentrations 45% (13/29) and 69% (20/29) of them are also identified for gene expression and NMD prone transcripts pathways.

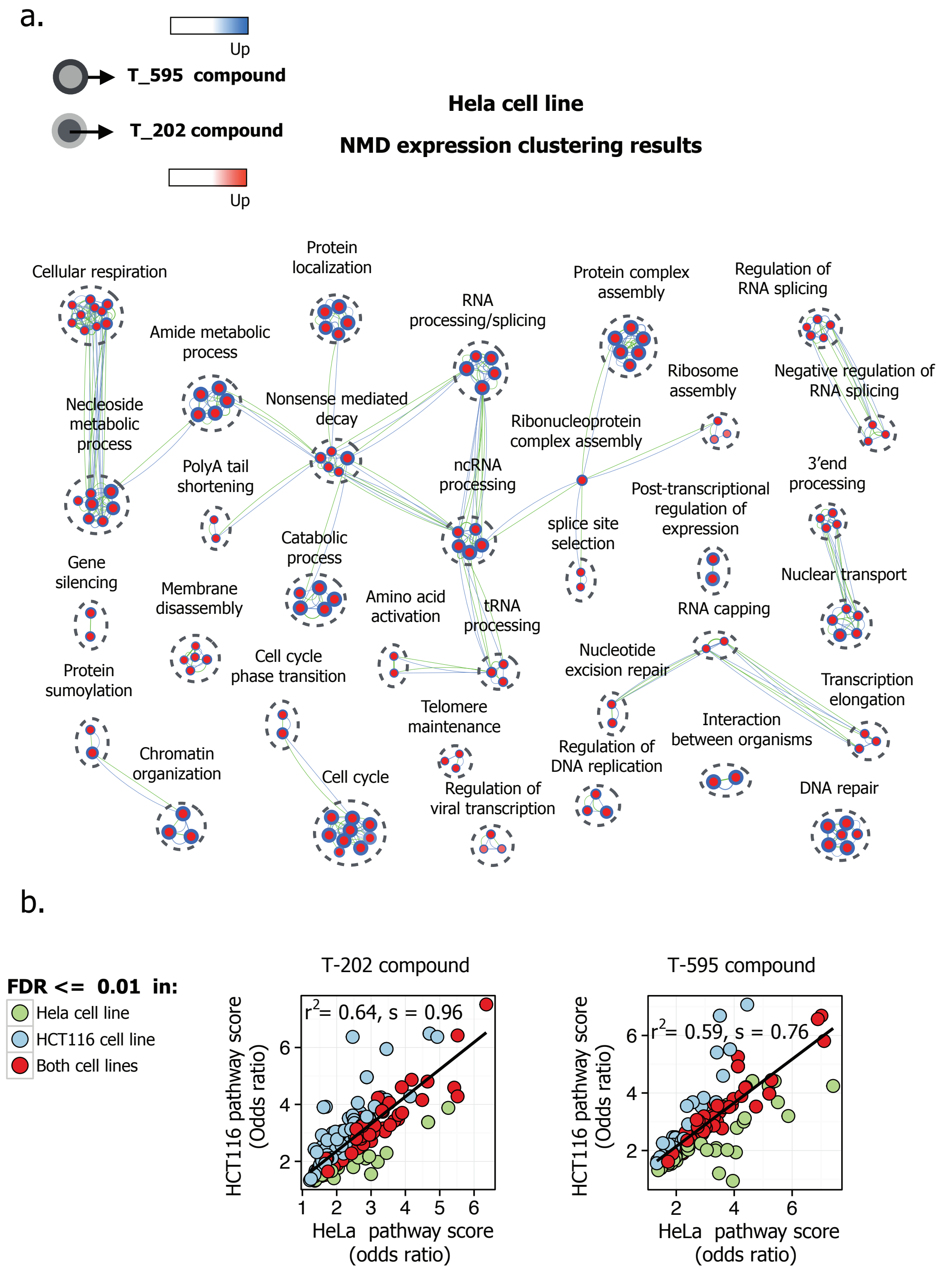

Supplementary Figure 12: Gene ontology enrichment analysis for the set of genes with NMD-prone transcripts showing monotonic increasing patterns of expression values (method section) based on WGCNA clustering. **a.** Each node represents a biological process with genes over-represented in the set of monotonically increasing clusters for HeLa cell line for each of the two active compounds (FDR: 0.01). Node cores and node rings illustrate the results for T-202 and T-595 compounds, and green and blue edges indicate the overlap between the identified genes of gene sets for T-202 and T-595 compounds, respectively. Biological processes are clustered using EnrichmentMap[32] based on the overlap of their monotonic genes. **b.** The plots present a comparison of the enrichment scores (x and y axes) for pathways identified in T-202 or T-595 compound libraries with FDR values <0.01. Odds scores are calculated by dividing the percentage of the genes in a given GO set that are identified by the percentage of all the genes that are identified.

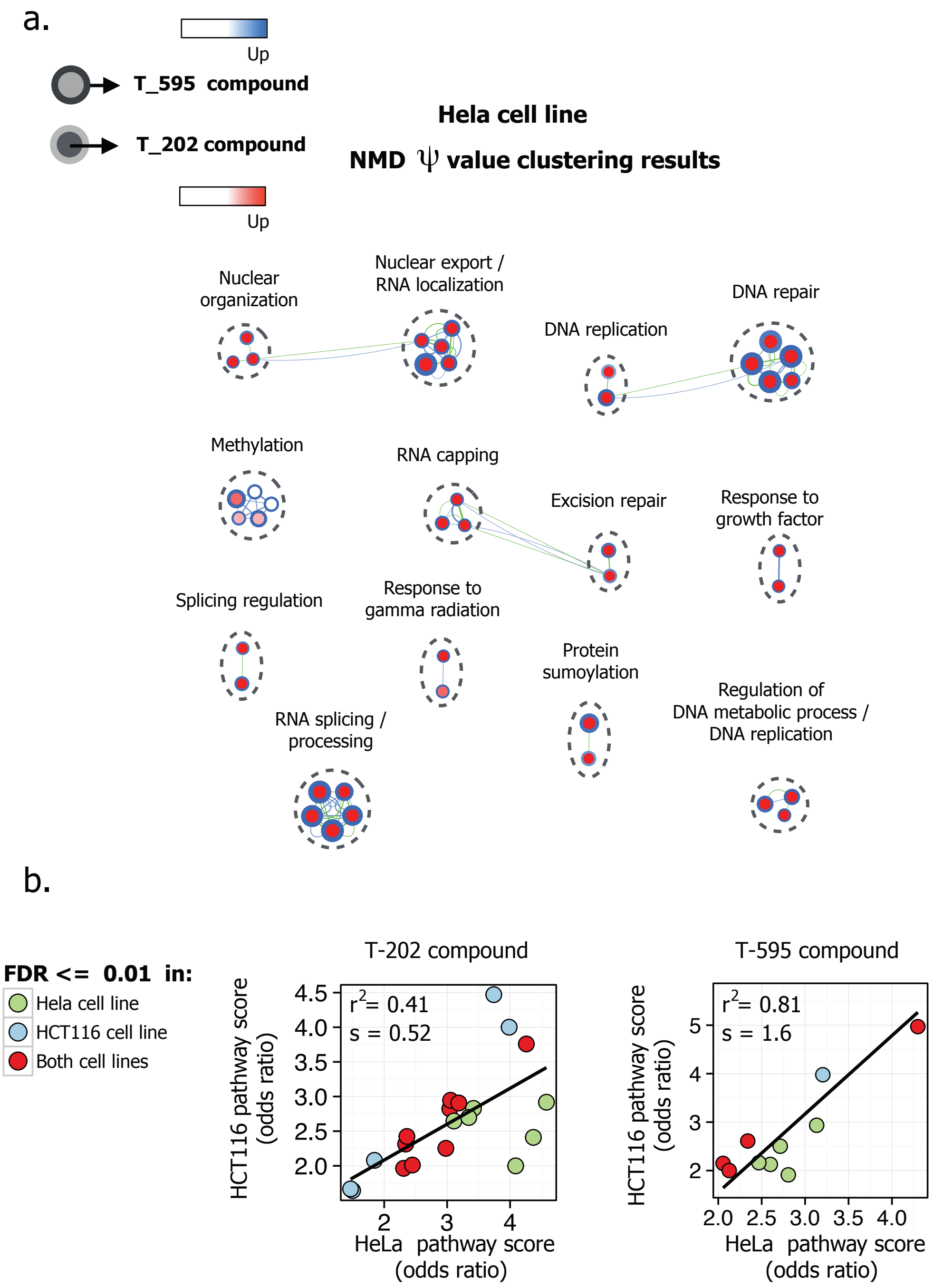

Supplementary Figure 13: GO enrichment analysis for the set of genes with NMD-prone transcripts showing monotonic increasing patterns of inclusion ratios (method section) based on WGCNA clustering. **a.** Each node represents a biological process with genes over-represented in the set of monotonically increasing clusters for HeLa cell line for each of the two active compounds (FDR: 0.1). Node cores and node rings illustrate the results for T-202 and T-595 compounds, and green and blue edges indicate the overlap between the identified genes of gene sets for T-202 and T-595 compounds, respectively. Biological processes are clustered using EnrichmentMap[32] based on the overlap of their monotonic genes. **b.** The plots present a comparison of the enrichment scores (x and y axes) for pathways identified in T-202 or T-595 compound libraries with FDR values <0.01. Odds scores are calculated by dividing the percentage of the genes in a given GO set that are identified by the percentage of all the genes that are identified.

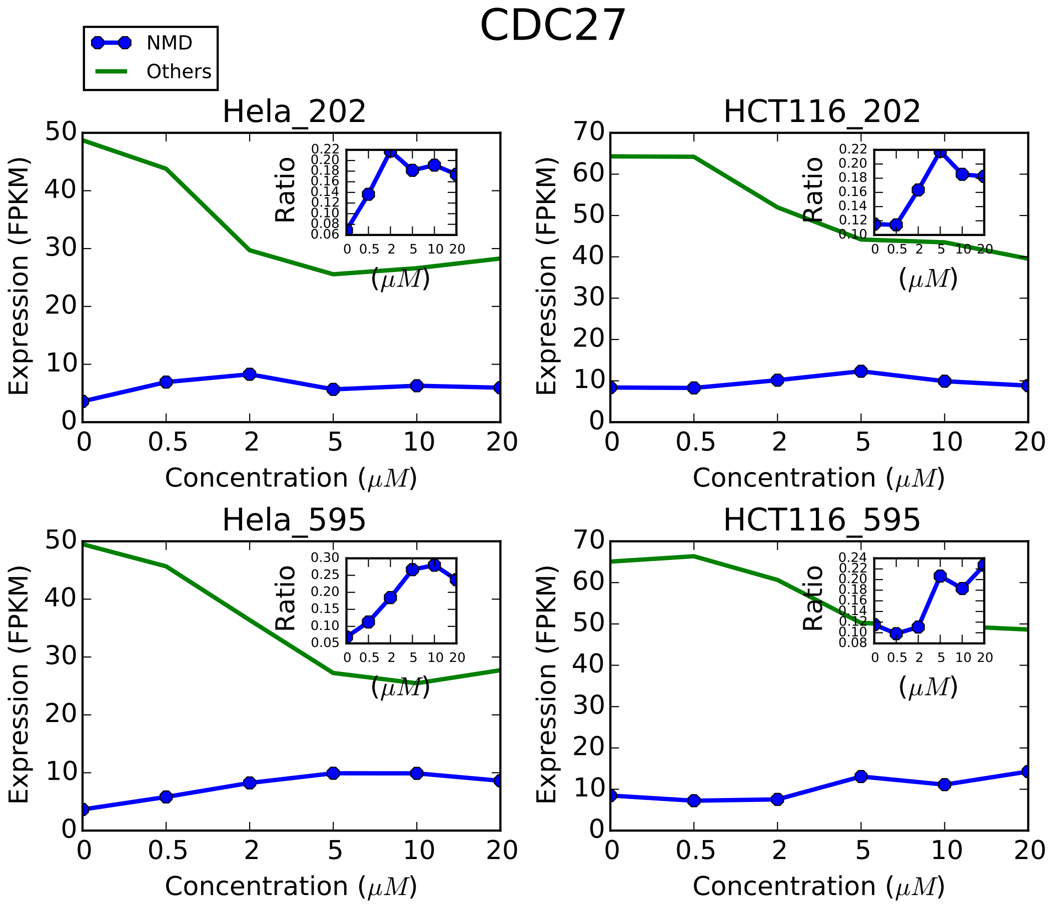

Supplementary Figure 14: Sum of expression values (FPKM) of NMD prone transcripts and the other transcripts for CDC27 gene. Results are shown for the two eutomers and the two cell lines. X axis shows drug concentrations and Y axis represents sum of FPKM values for each group. Small plots show the ratio of the sum of NMD prone transcripts to total gene expression at different drug concentrations. The ratio of the expression of NMD prone transcripts to gene expression increases by increasing drug level as a result of interfering with NMD mechanism.

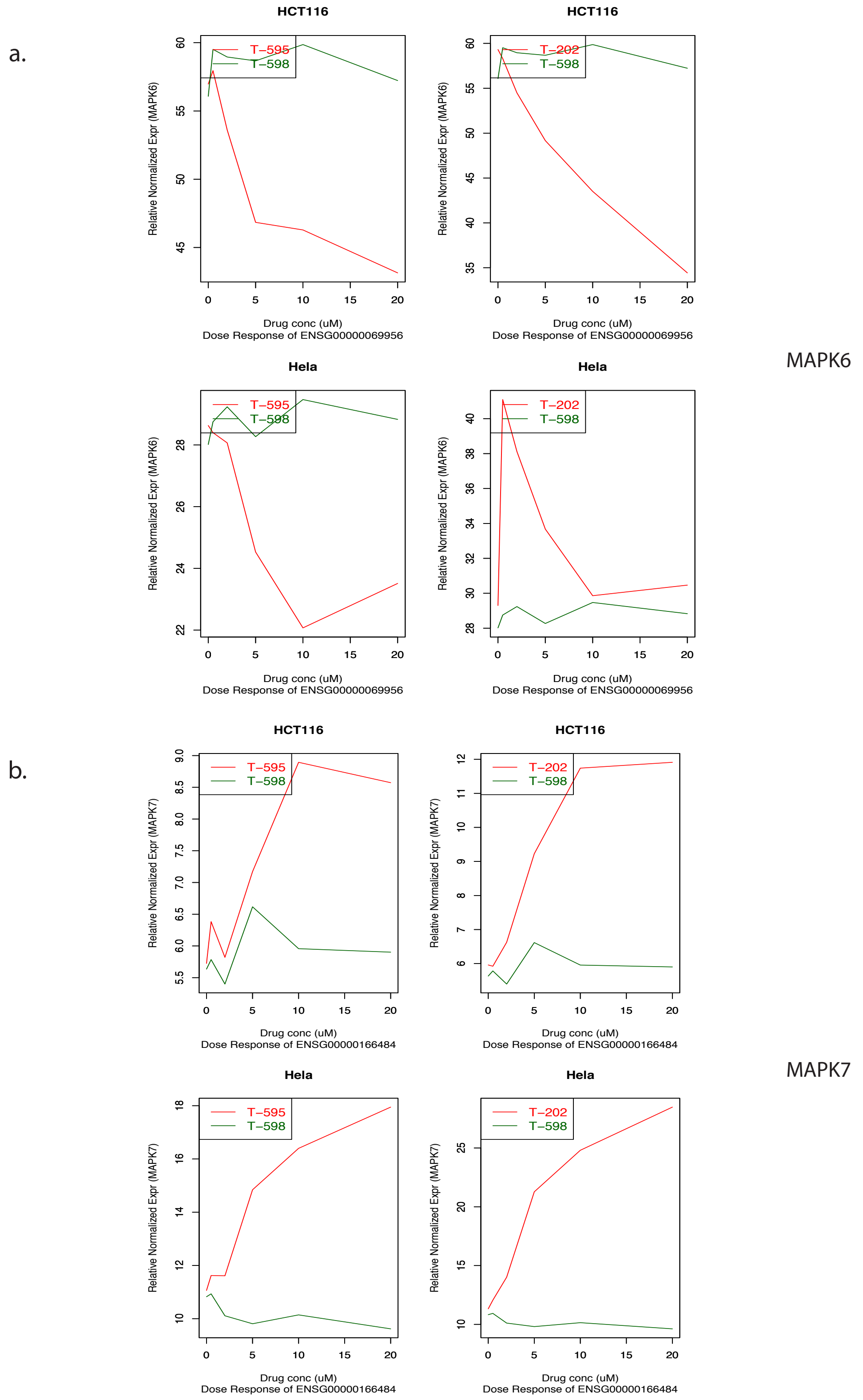

Supplementary Figure 15: Relative normalized expression (vertical axes) plots of the transcripts of **a.** MAPK6 and **b.** MAPK7 in HCT-116 and HeLa cells in response to drugs (horizontal axes), i.e. active eutomers (red trend line) and inactive distomers (green trend line).

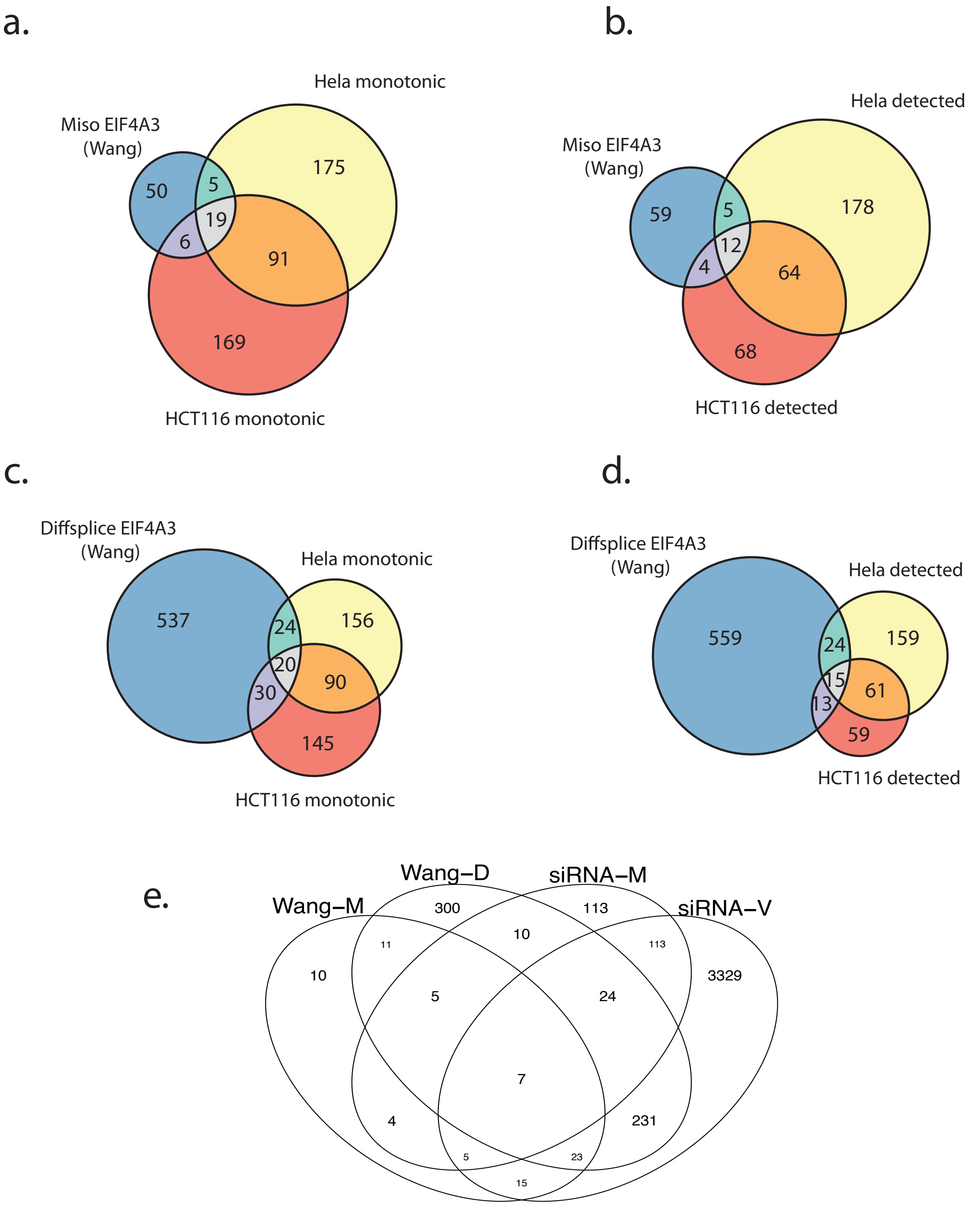

Supplementary Figure 16: Comparing genes undergoing AS regulation via graded inhibition in our study to a previously reported AS genes when treating HeLa cells with siRNAs directed to EIF4A3[5]. In parts **a-e**, MISO EIF4A3 (Wang): unique genes detected in Wang *et al.* using MISO (common in replicates), Diffsplice EIF4A3 (Wang): unique genes detected in Wang *et al.* using DiffSplice method[33], HeLa/HCT116 monotonic: unique genes with events showing monotonic Ψ patterns in HeLa/HCT116 cell lines (common between T-595 and T-202), HeLa/HCT116 detected: Set of genes with AS events detected at least twice among five compound doses (common between T-595 and T-202). **e** Venn diagram showing the number of genes common between the two studies (Wang et al (Wang) and ours (siRNA)) using the various methods MISO (M), VAST-tools (V) or Diffsplice (D). The gene list can be found in Supplemental Data 12.

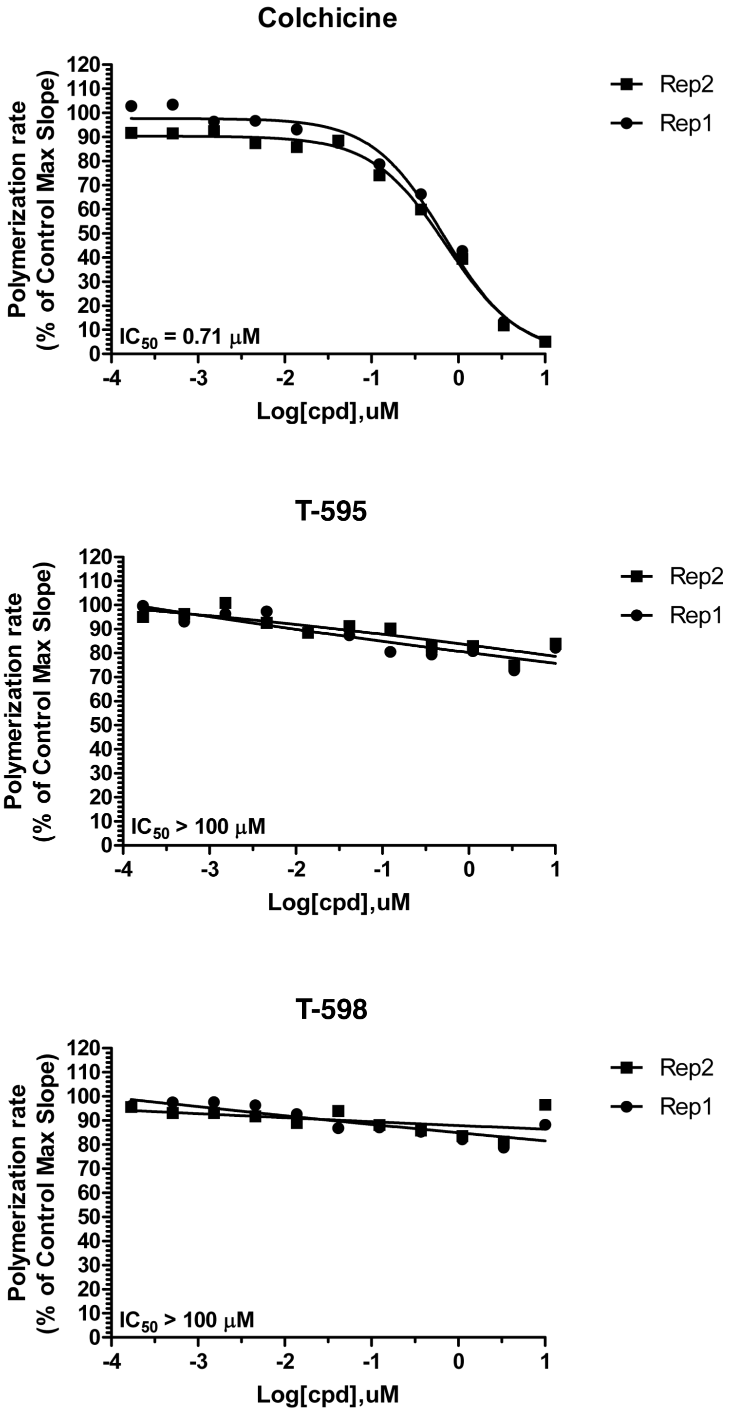

Supplementary Figure 17: IC_{50} values for the inhibition of tubulin polymerization for T-595 (**middle**) and T-598 (**bottom**) compounds using colchicine (**top**) as the inhibitor control and pure porcine neuronal tubulin using the Tubulin Assay Kit by the manufacturer (Cytoskeleton Inc, CO, USA). The maximal velocity (Maximum slope, called V_{max}) of tubulin polymerization was determined using Softmax Pro software version 3.5 software and the mean of duplicate maximum slope determinations at each concentration were then normalized to reactions that contained DMSO (2% final concentration) but no test compounds and were converted to % Activity using the following formula. % Polymerization rate = 100 x (V_{max}(compound)/V_{max}(DMSO)

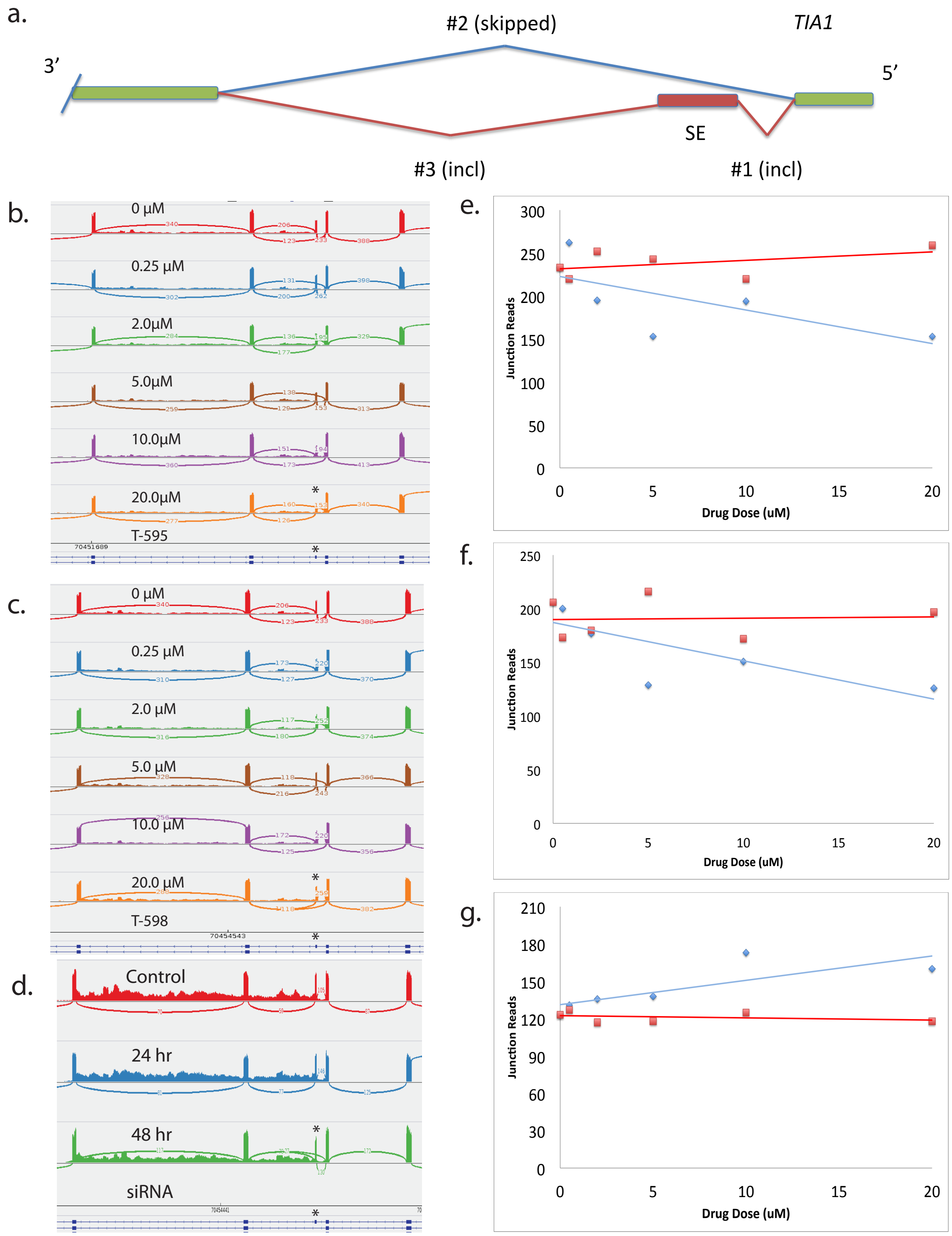

Supplementary Figure 18: Splicing at the *TIA1* (ENSG00000116001) locus. **a.** Schematic diagram showing how exon 5 (SE) is skipped as a result of T-595 dose dependent alternative splicing events. Sashimi plot showing the splicing events (min. 30 junction reads) when treated with various doses of either **b.** T-595 or **c.** T-598 in HCT-116 cells or **d.** siRNA to *EIF4A3* in HeLa cells . The skipped exon 5 is highlighted with a star (*). Junction reads when exon 5 is included (**e.** events #1 and **f.** #3 from **a.**) or skipped (**g.** event #2 from **a.**) when treated with respective doses of T-595 (blue dots and trend line) or T-598 (red dots and trend line) in HCT-116 cells.

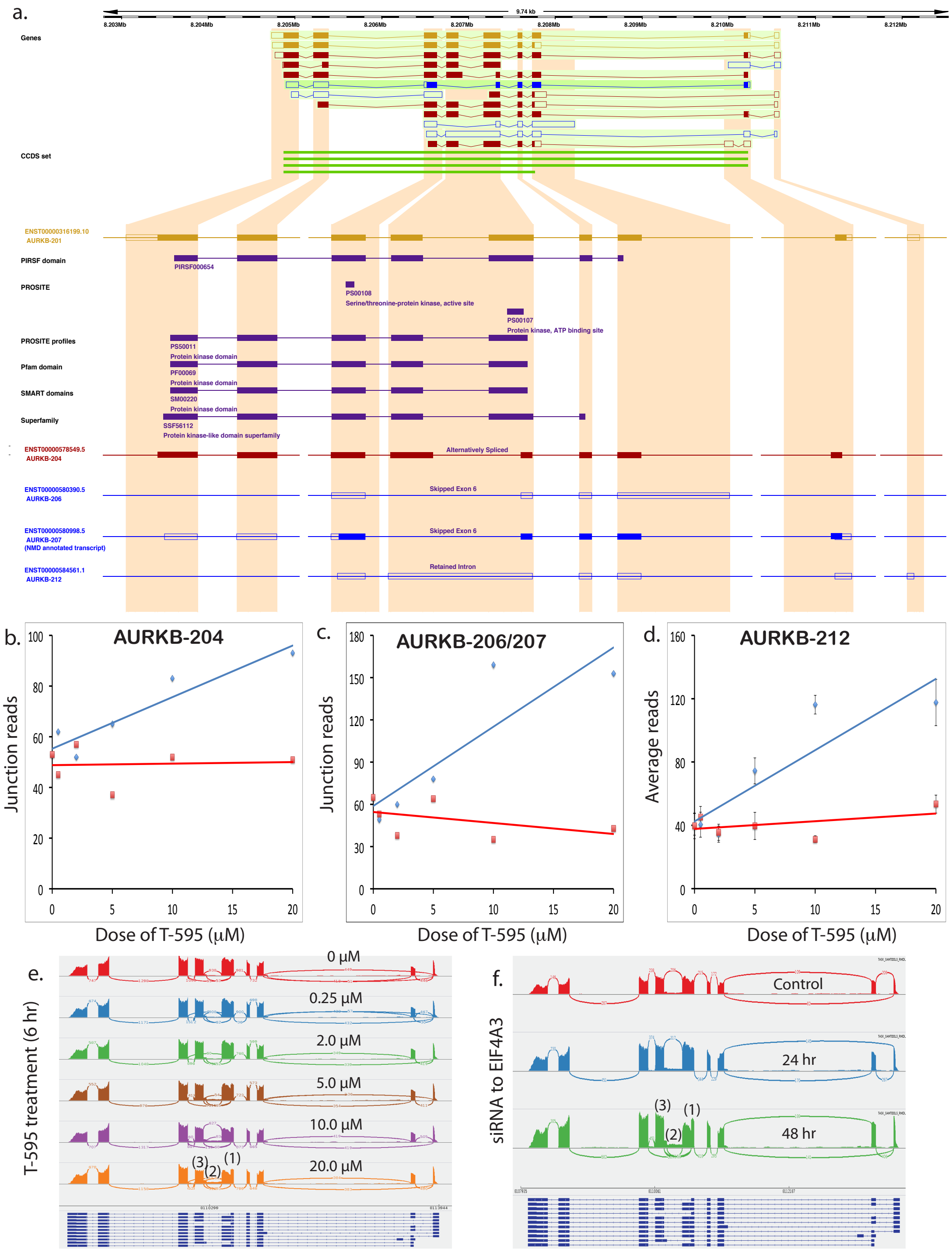

Supplementary Figure 19: Locus of *AURKB* showing alternatively spliced variants as observed when treated with EIF4A3 inhibitors in HCT-116. **a.** Edited screen shot of the *AURKB* locus from Ensembl showing relevant protein coding domain information and highlighting the various spliced transcripts: **b.** Alternatively Spliced (*AURKB-204*, plot of junction reads (vertical axis) from sashimi plot) **c.** Skipped Exon 6 (*AURKB-206/207*, plot of junction reads (vertical axis) from sashimi plot; note that *AURKB-207* is an annotated NMD-transcript) and **d.** Retained Intron (*AURKB-212*, average reads (vertical axis) from alignment files) when HCT-116 cells were treated with different doses (horizontal axes) of T-595 (blue dots & trend line) and T-598 (red dots & trend line). Sashimi plots of showing the **e.** dose dependent splicing events (min. 30 reads) when treated with T-595 at the given doses in HCT-116 cells or **f.** transfected with siRNA targeting EIF4A3 in HeLa cells. Annotation: (1) = Alternatively Spliced (*AURKB-204*), (2) = Retained Intron (*AURKB-212*), (3) = Skipped Exon 6 (*AURKB-206/207*).

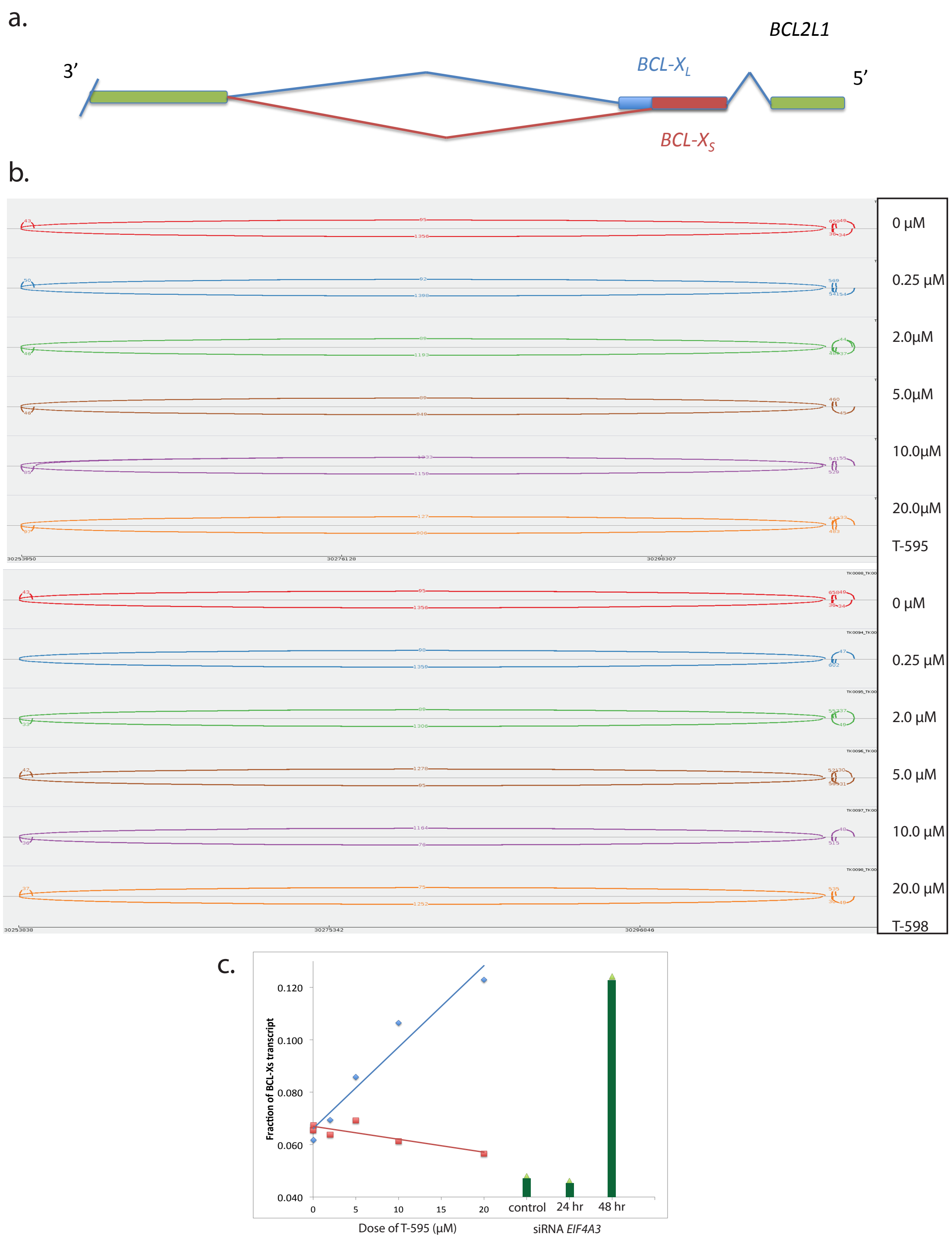

Supplementary Figure 20: Splicing of the *BCL2L1* locus. **a.** Schematic diagram showing how $BCL-X_L$ and $BCL-X_S$ are generated from transcripts resulting from alternative splicing events. **b.** Sashimi plot showing the splicing events (min. 30 reads) when treated with various doses of either T-595 or T-598 in HCT-116 cells. **c.** Fraction of the $BCL-X_S$ over total events ($BCL-X_L$ + $BCL-X_S$ reads) when treated with respective doses of T-595 (blue dots and trend line) or T-598 (red dots and trend line) in HCT-116 cells or siRNA targeting *EIF4A3* (green triangles and bars) at the respective times post-transfection in HeLa cells.

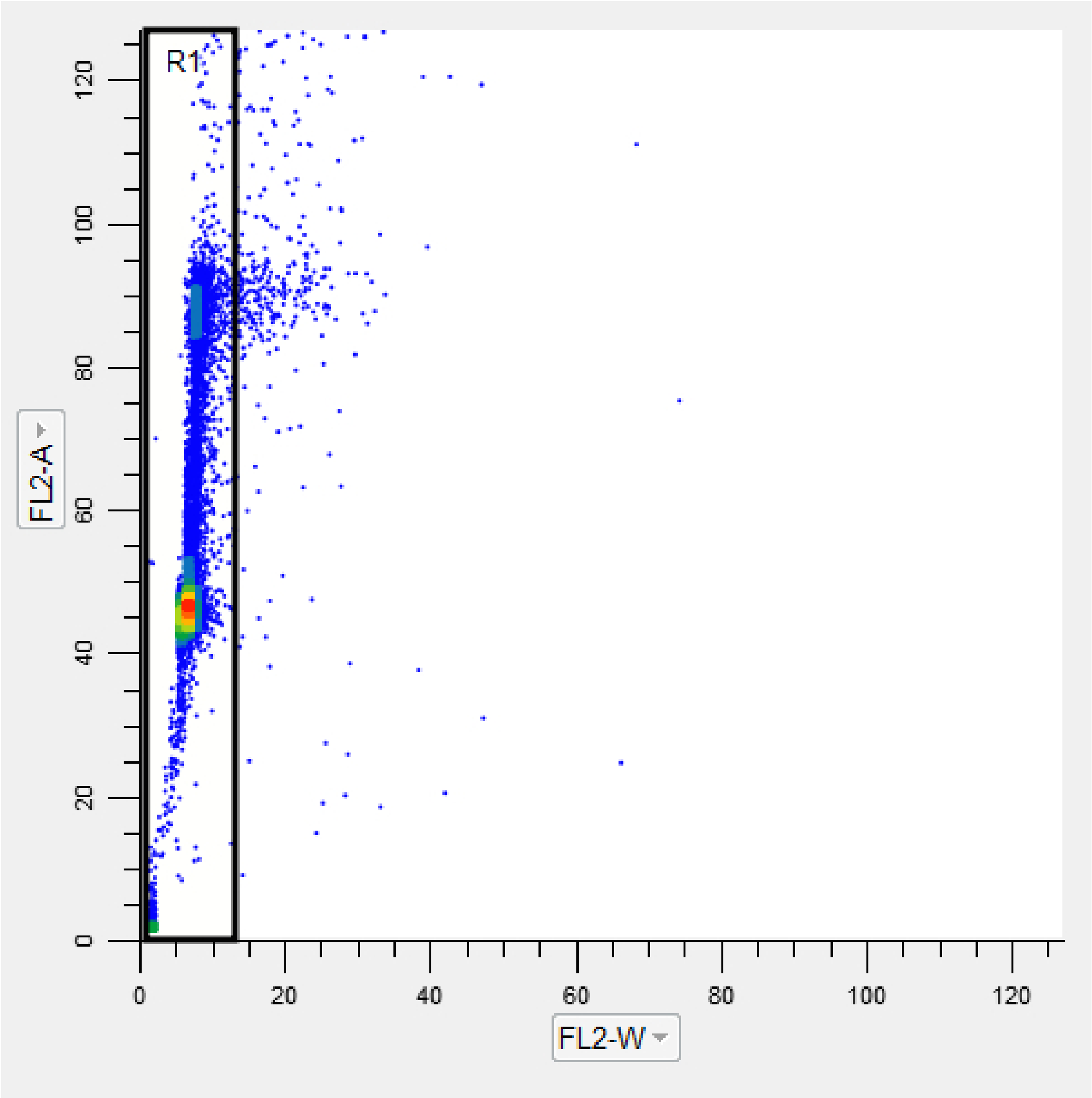

Supplementary Figure 21: Cells treated with Cycle Test Plus DNA Reagents for the determination of the cellular DNA contents (by propidium iodide (PI) incorporation) by flow cytometry on a BD FACSVerse ™. The singlet cell region of the FL2-A (PI) vs. FL2-W dot plot was gated (R1) as shown. FL2-W was used to discriminate singlet and aggregated cells. The abundance of cells in the post-sort fraction was typically over 90 % of the population by downstream analysis of ModFit software.

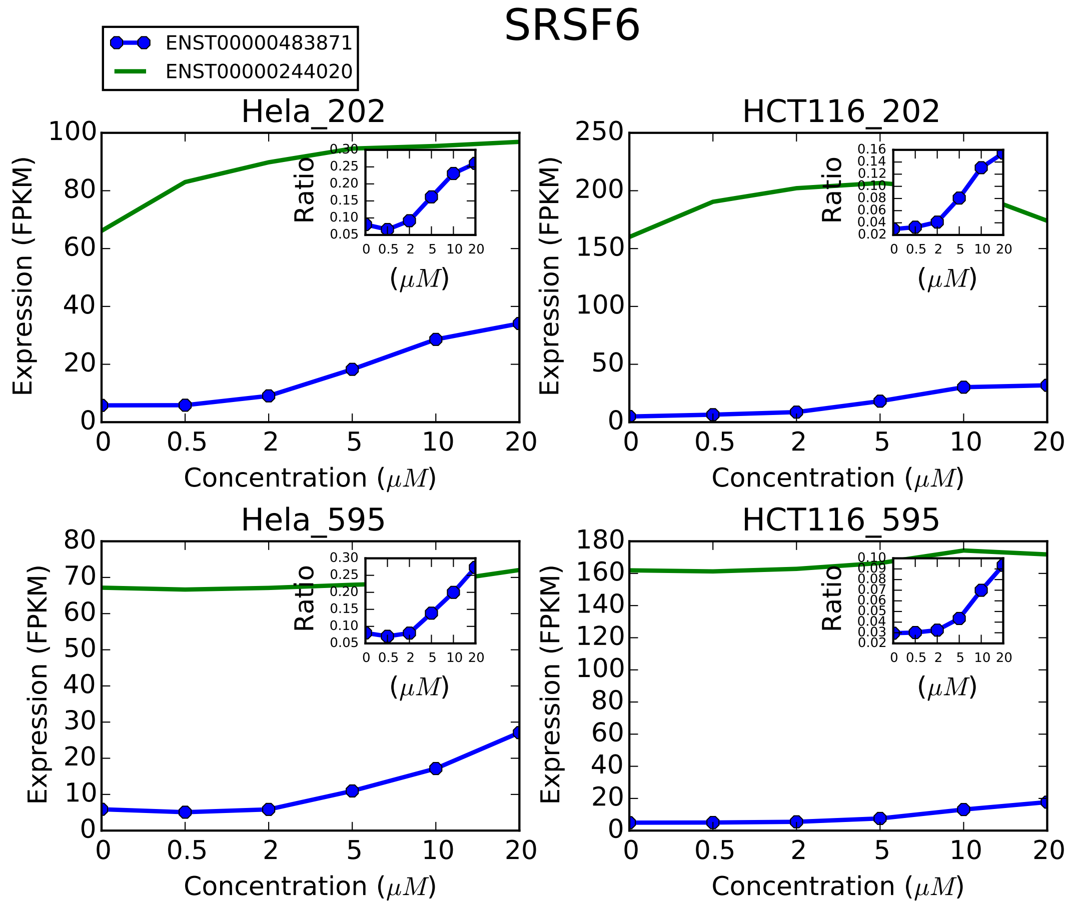

Supplementary Figure 22: Expression values (FPKM) of the two transcripts of SRSF6 genes annotated by Ensembl. One of these two transcripts is annotated to be NMD prone (ENST00000483871). Results are shown for the two eutomers and the two cell lines. X axis shows drug concentrations and Y axis represents sum of FPKM values for each group. Small plots show the ratio of ENST00000483871 expression to total gene expression at different drug concentrations. The ratio of the expression of the NMD prone transcript to gene expression increases by increasing drug level as a result of interfering with NMD mechanism.

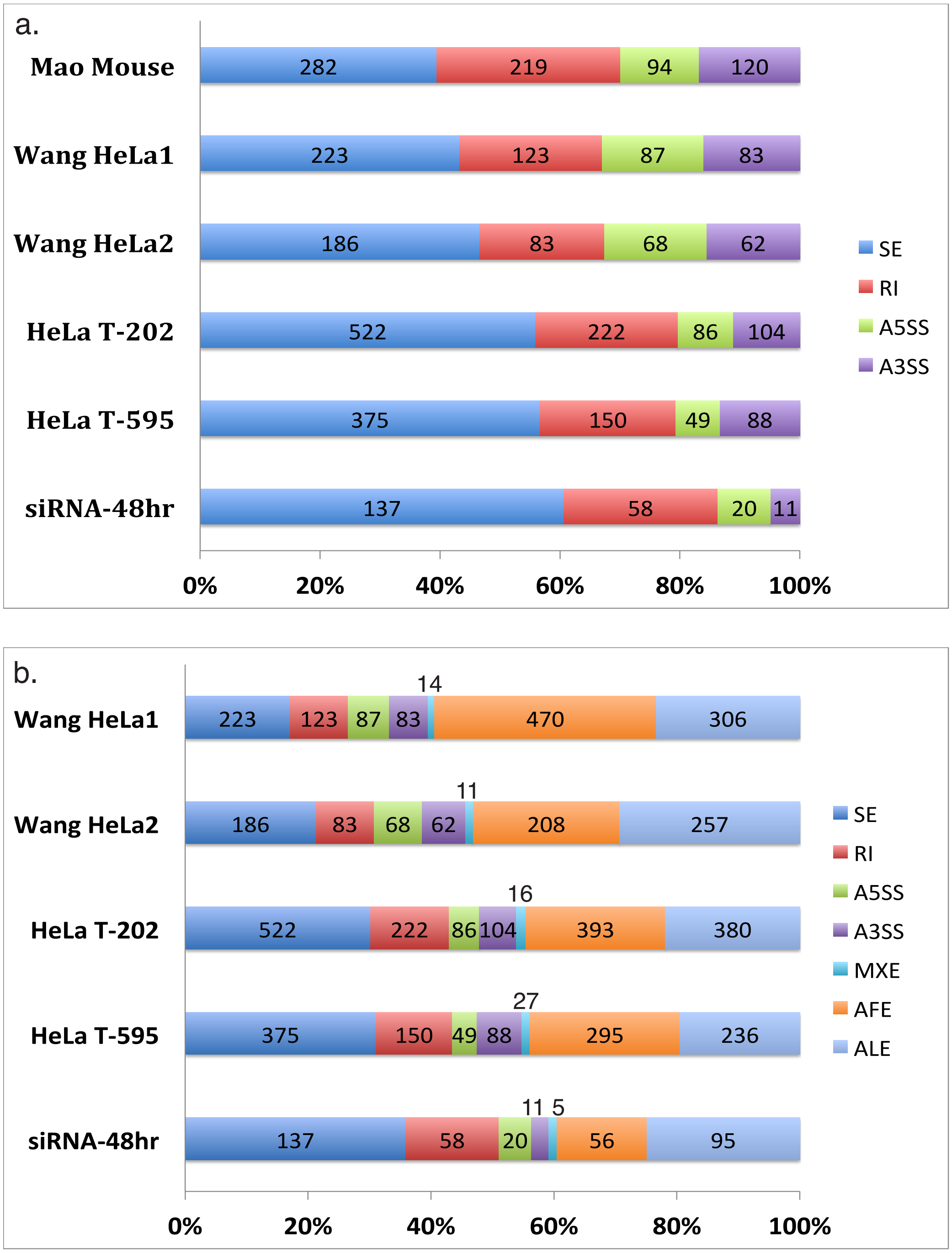

Supplementary Figure 23: Comparison of the overall splicing proportions on treatment between drug-treated, T-202 or T-595 (high dose, 6 hr treatment) and siRNA directed to *EIF4A3* (48 hr treatment) in HeLa cells from our experiments and with the respective published data[4, 5]. **a.** Comparison of the proportions of the 4 slice events (SE, RI, A5SS and A3SS) between the mouse data (as published[4]) and proportions determined by MISO from siRNA *EIF4A3* from published data[5] and drug and siRNA to *EIF4A3* from our datasets. **b.** Comparisons the proportions of the splice events as determined by MISO in the published dataset[5] and our datasets.

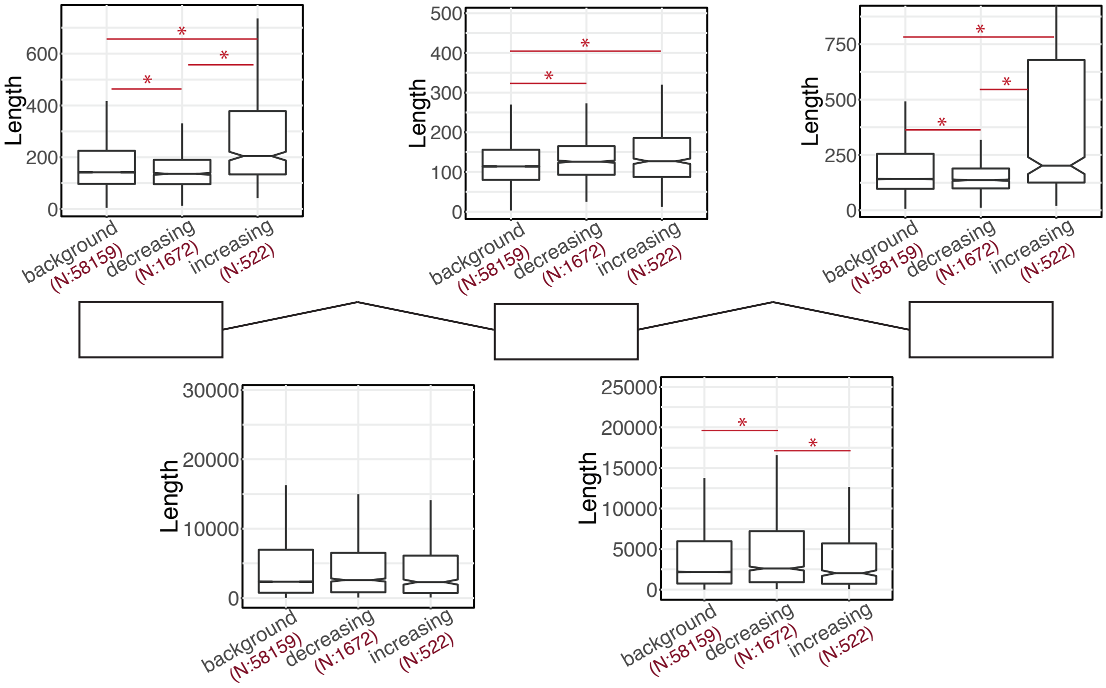

Supplementary Figure 24: Length distribution of introns and exons in background SE events, SE events with monotonically increasing Ψ responses, and SE events with monotonically decreasing Ψ responses. The exons are generally longer for the set of monotonically increasing responsive events compared to both background and the monotonically decreasing sets. Red stars show comparisons where p-values of Mann-whitney U tests are <0.01. p-values of comparisons of [background:increasing, background:decreasing, increasing:decreasing] for the exons and introns accordingly from 5' to 3' end (left to right) are: [8.9*10$^-$33, 3.7*10$^-$5, 1.1*10$^-$38], [0.48, 0.73, 0.42], [3.9*10$^-$8, 5.3*10$^-$18, 0.27], [0.49, 6.2*10$^-$5, 0.007], [1.5*10$^-$25, 0.0003, 2.36*10$^-$30].

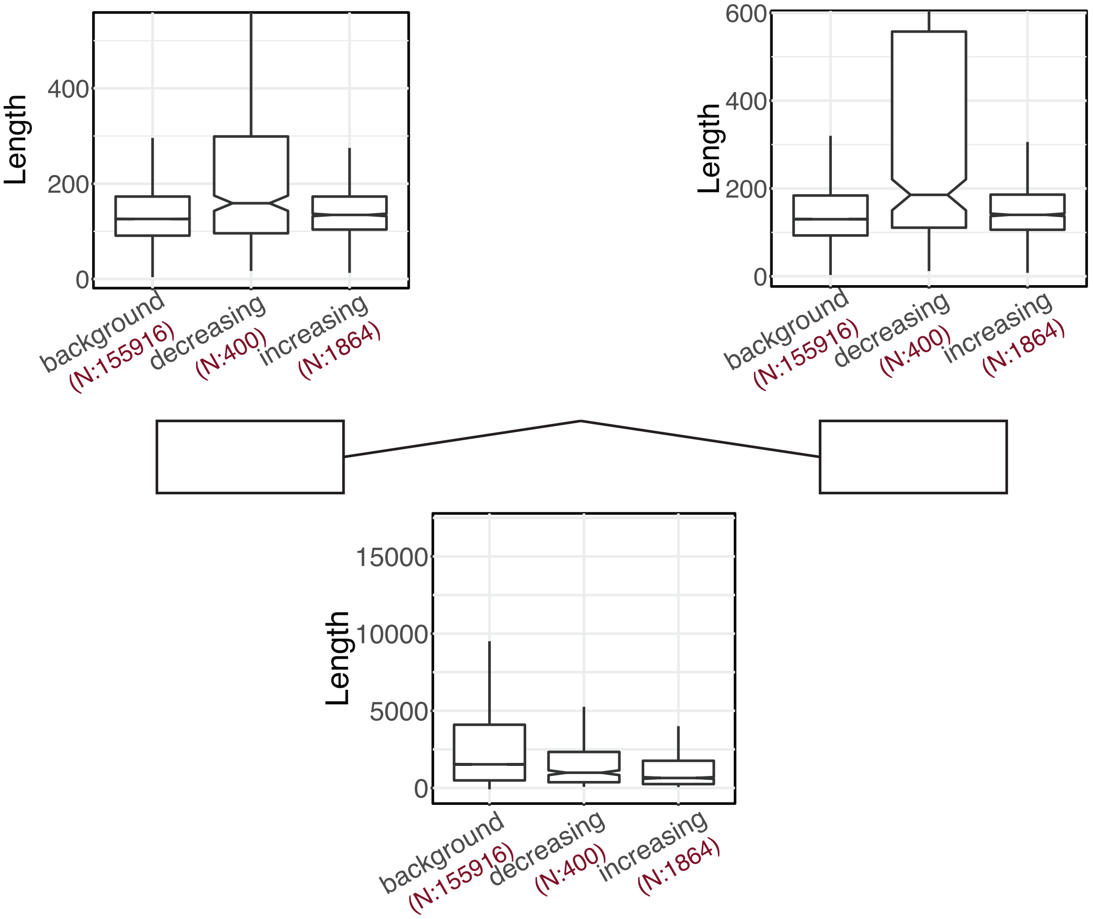

Supplementary Figure 25: Length distribution of introns and exons in background RI events, RI events with monotonically increasing Ψ responses, and RI events with monotonically decreasingΨ responses. The exons are generally longer for the set of monotonically decreasing responsive events compared to both background and the monotonically increasing sets, and introns are shorter for both classes of identified RI events compared to background (p-values <0.01 for all comparisons; Mann-whitney U test). p-values of comparisons of [background:increasing, background:decreasing, increasing:decreasing] for the exons and introns accordingly from 5' to 3' end (left to right) are: [$3.9*10^{-9}$, $2.0*10^{-12}$, $6.9*10^{-7}$], [$7.8*10^{-99}$, $8.7*10^{-8}$, $4.6*10^{-6}$], [$6.3*10^{-11}$, $3.6*10^{-19}$, $1.2*10^{-11}$].

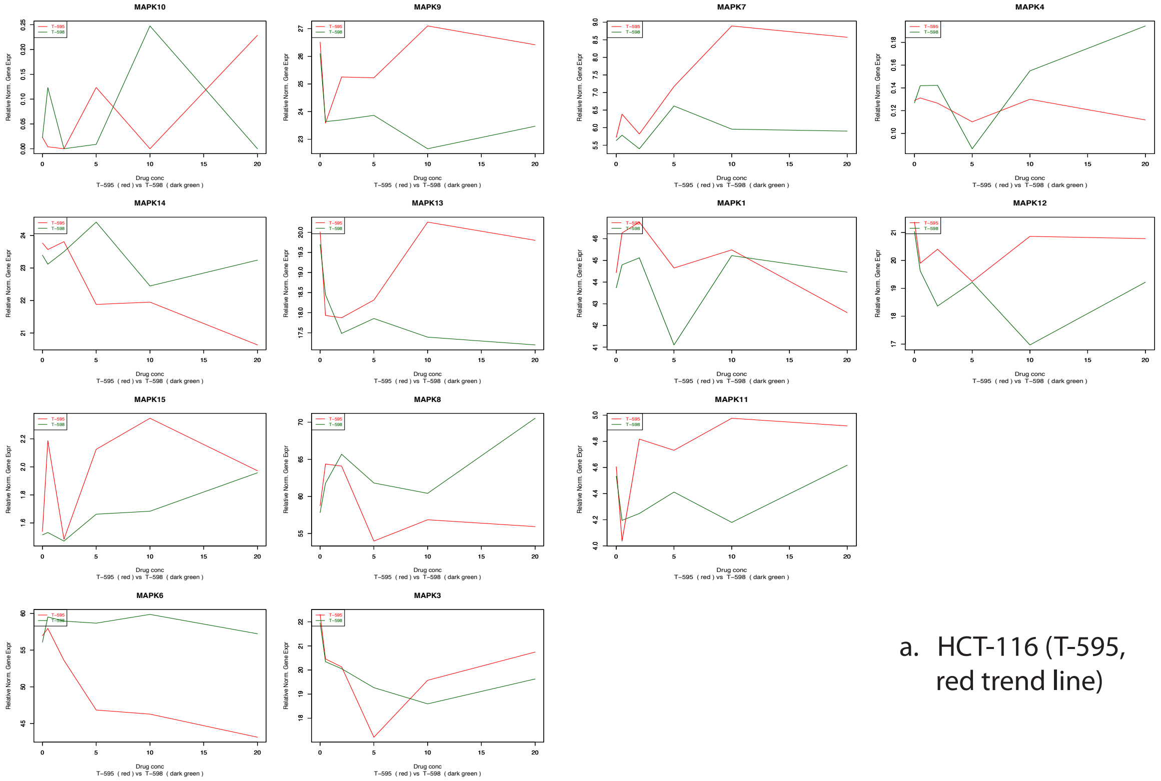

a. HCT-116 (T-595, red trend line)

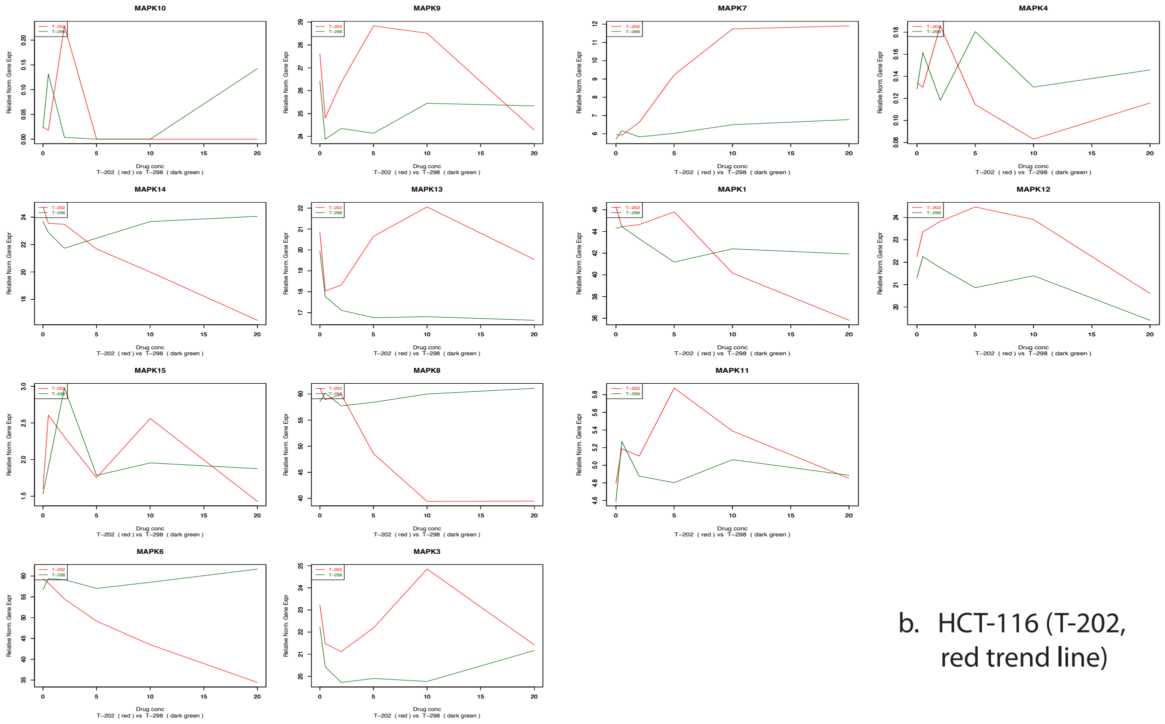

b. HCT-116 (T-202, red trend line)

Supplementary Figure 26: Relative normalized expression (vertical axes) plots of the expressed MAPK transcripts in HCT-116 in response to various concentrations active eutomers (horizontal axes) **a.** T-595 or **b.** T-202 (red trend lines) and inactive distomers (green trend lines).

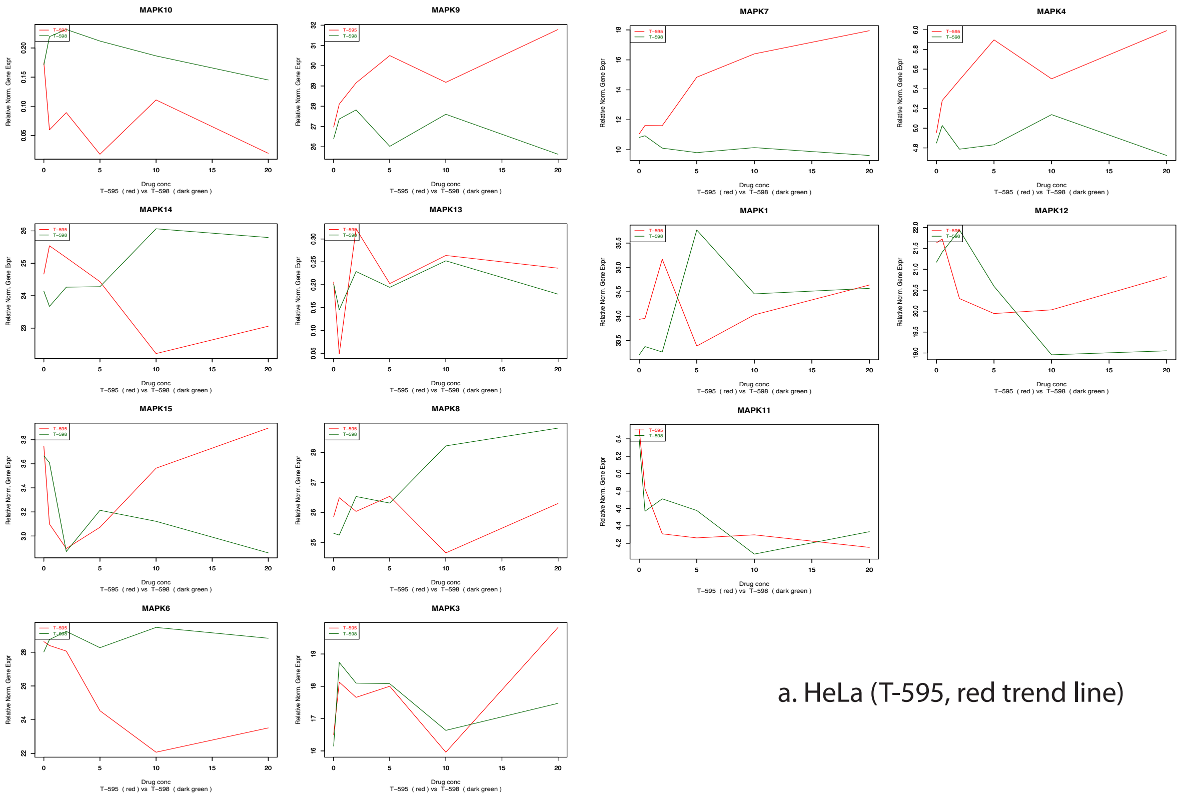

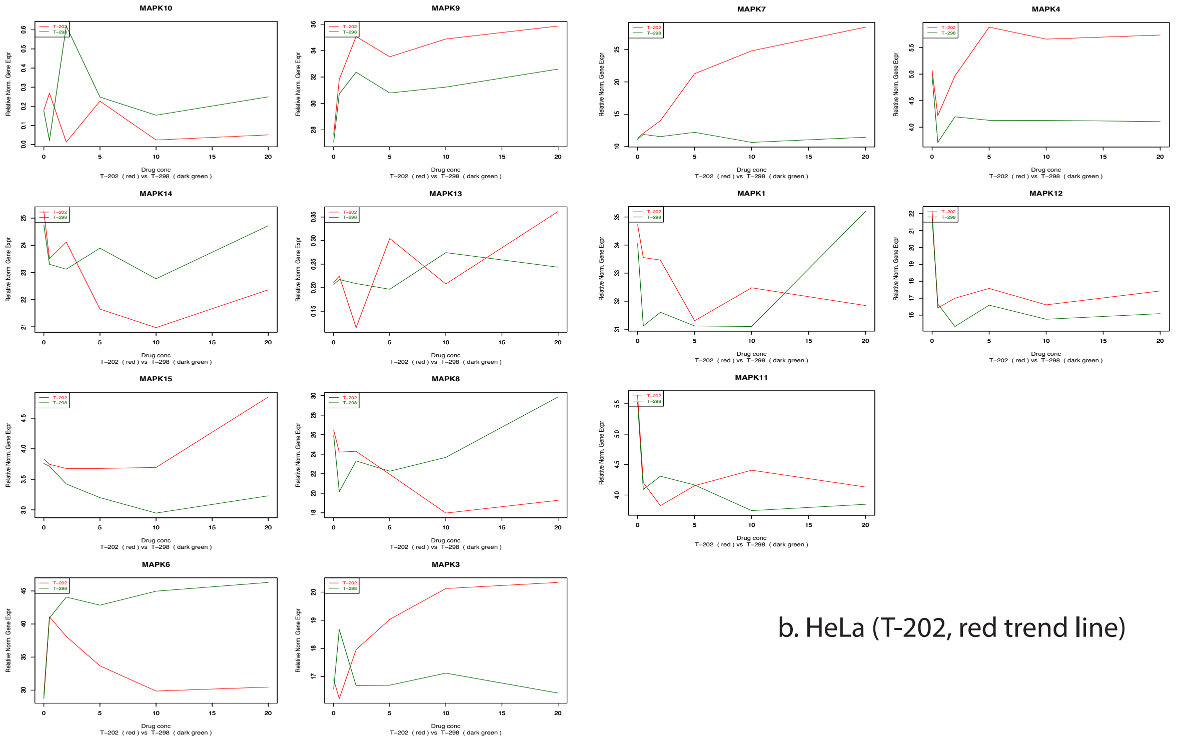

Supplementary Figure 27: Relative normalized expression (vertical axes) plots of the expressed MAPK transcripts in HeLa in response to various concentrations active eutomers (horizontal axes) **a.** T-595 or **b.** T-202 (red trend lines) and inactive distomers (green trend lines).

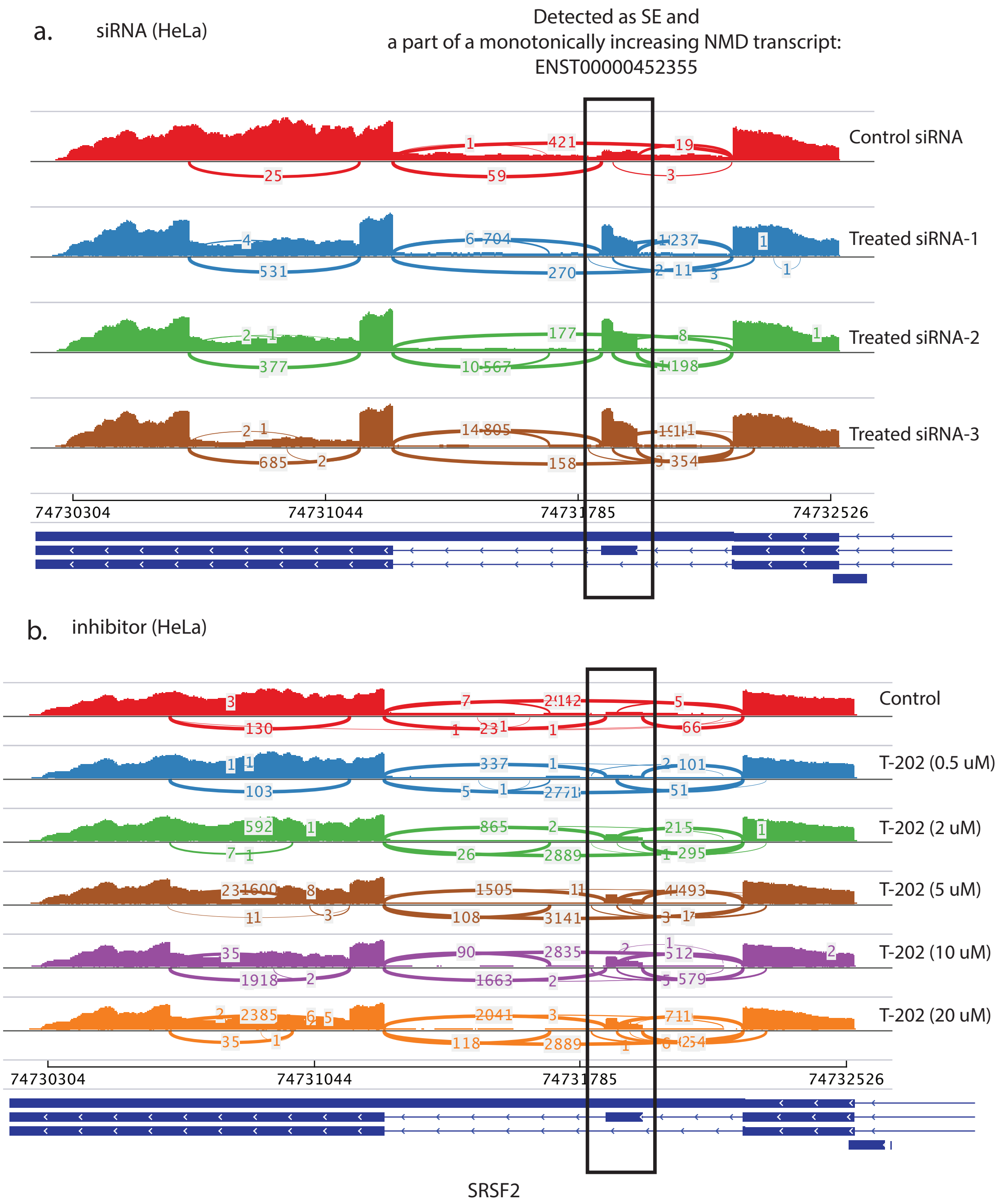

Supplementary Figure 28: Sashimi plot showing that SRSF2 undergoes AS regulation upon inhibiting EIF4A3 using active compounds or when it is knocked down by siRNAs. Results are shown for HeLa cell line. **a.** Change in SRSF2 splicing in EIF4A3 knock down experiment. The exon in black box is a part of an NMD transcript with monotonically increasing expression pattern, and the plot shows increase in its inclusion level. **b.** A similar pattern with gradual increase in the NMD exon is observed upon increasing inhibition level using T-202 compound.

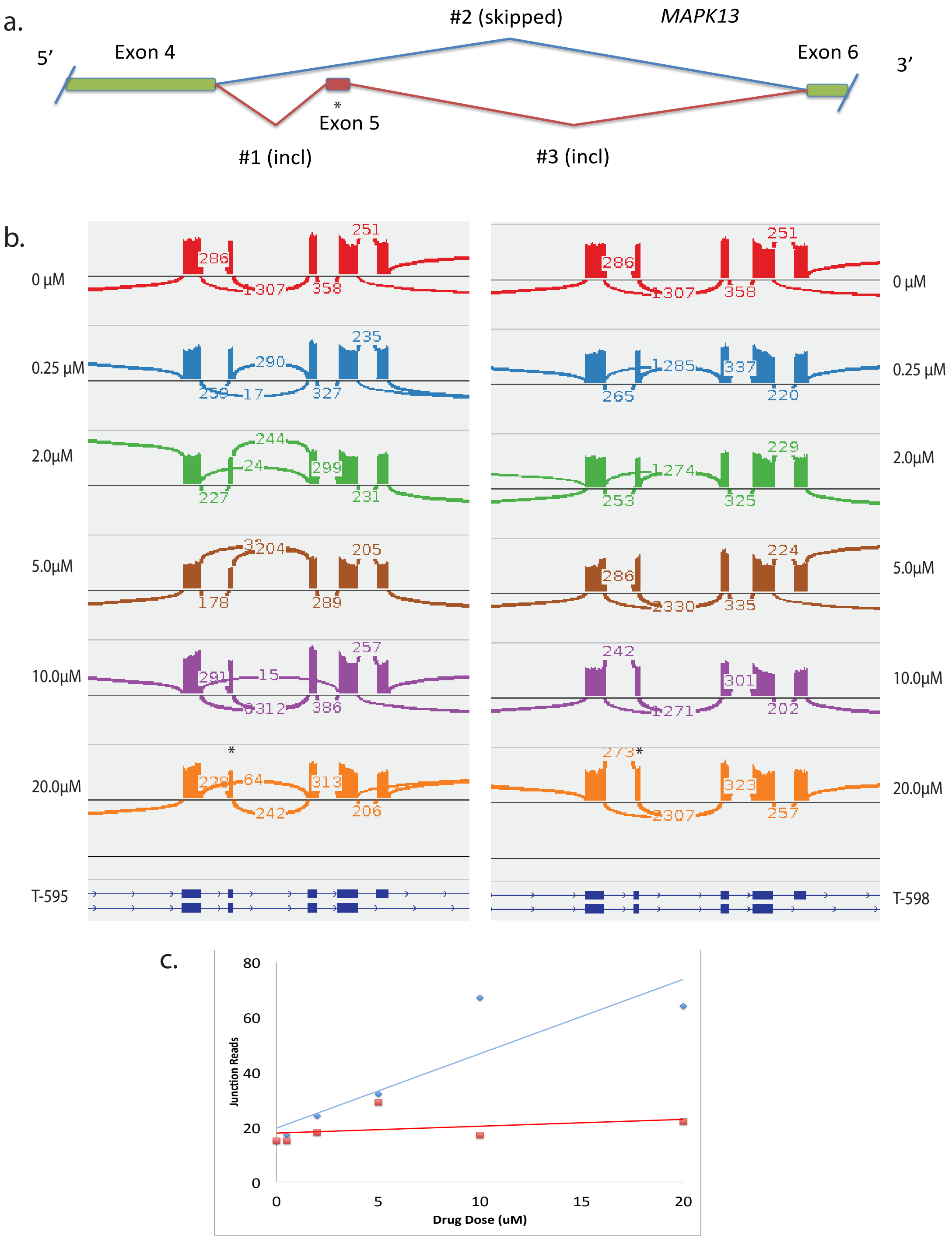

Supplementary Figure 29: Splicing at the *MAPK13* (ENSG00000156711) locus. **a.** Schematic diagram showing how exon 5 (SE) is skipped as a result of T-595 dose dependent alternative splicing events. **b.** Sashimi plot showing the splicing events (min. 15 junction reads) when treated with various doses of either **left.** T-595 or **right.** T-598 in HCT-116 cells. The skipped exon 5 is highlighted with a star (*). **c.** Junction reads when exon 5 is skipped (event #2 from **a.**) when treated with respective doses of T-595 (blue dots and trend line) or T-598 (red dots and trend line) in HCT-116 cells.

Library	Cell line	Inhibitor	Treatment (µM)	siRNA	Total Reads
TK0017, TK0021	HeLa	Control		RNAi	62,950,833
TK0018, TK0019, TK0020	HeLa	eIF4A3 (24 hr)		RNAi	99,185,262
TK0022, TK0023, TK0024	HeLa	eIF4A3 (48 hr)		RNAi	103,609,971
TK0077	HeLa	-	0.0		139,550,167
TK0078	HeLa	T-595	0.5		133,154,090
TK0079	HeLa	T-595	2.0		132,006,108
TK0080	HeLa	T-595	5.0		137,777,582
TK0081	HeLa	T-595	10.0		114,397,074
TK0082	HeLa	T-595	20.0		144,837,055
TK0083	HeLa	T-598	0.5		133,428,479
TK0084	HeLa	T-598	2.0		132,674,628
TK0085	HeLa	T-598	5.0		128,515,111
TK0086	HeLa	T-598	10.0		144,020,091
TK0087	HeLa	T-598	20.0		119,271,159
TK0088	HCT116	-	0.0		145,124,178
TK0089	HCT116	T-595	0.5		142,408,505
TK0090	HCT116	T-595	2.0		129,983,858
TK0091	HCT116	T-595	5.0		116,353,204
TK0092	HCT116	T-595	10.0		145,673,544
TK0093	HCT116	T-595	20.0		127,084,580
TK0094	HCT116	T-598	0.5		137,945,642
TK0095	HCT116	T-598	2.0		130,994,635
TK0096	HCT116	T-598	5.0		138,107,155
TK0097	HCT116	T-598	10.0		127,765,564
TK0098	HCT116	T-598	20.0		143,530,045
TK0099	HeLa	T-202	0.5		128,130,203
TK0100	HeLa	T-202	2.0		133,398,356
TK0101	HeLa	T-202	5.0		142,622,020
TK0102	HeLa	T-202	10.0		115,971,472
TK0103	HeLa	T-202	20.0		133,066,045
TK0109	HCT116	T-202	0.5		142,998,863
TK0110	HCT116	T-202	2.0		138,803,402
TK0111	HCT116	T-202	5.0		137,842,657
TK0112	HCT116	T-202	10.0		148,418,672
TK0113	HCT116	T-202	20.0		149,721,568

Supplementary Table 1: Summary the conditions and total reads of the RNA-Seq libraries

Type	Unique count	Detected ratio (%)
A3SS	250	1.67
A5SS	185	1.44
AFE	807	3.76
ALE	649	5.87
MXE	71	2.60
RI	443	7.40
SE	1279	3.26
TandemUTR	125	4.71

Supplementary Table 2: Abundance of the given alternatively spliced events as identified by MISO analysis. Legend: Alternative 3' and 5' splice sites (A3SS, A5SS), alternative first and last exons (ALE, AFE), Mutually exclusive exons (MXE), Retained introns (RI), Skipped exons (SE), Tandem 3' UTR (TandemUTR)

Type	Class	Unique count	Detected ratio (%)
Alt3	Alt3	939	4.50144
Alt5	Alt5	930	5.87307
SE	C1	937	10.3559
SE	C2	973	12.3556
SE	C3	1061	12.5266
SE	MIC	22	4.5929
SE	S	4047	8.28794
RI	IR-C	653	3.10494
RI	IR-S	1955	1.46049

Supplementary Table 3: Abundance of the given alternatively spliced events as identified by VAST-TOOLS analysis. Legend: Alternative 3' and 5' splice sites (Alt3,Alt5), Retained introns (RI) sub-classified by complexity into intron retention events (IR-C,IR-S), Skipped exons (SE) stratified according to complexity of events (C1, C2, C3, S) and micro exons (MIC).

Set	Type	p-value	TT-count	TF-count	FT-count	FF-count	TT/TF	FT/FF
Decreasing	RI	0.99	281	175	106805	53615	1.61	1.99
Decreasing	SE	7.91e-13	3301	1802	65028	43802	1.83	1.48
Increasing	RI	0.04	1398	646	105688	53144	2.16	1.99
Increasing	SE	0.08	807	496	67522	45108	1.63	1.50

Supplementary Table 4: Testing using Fisher's exact test whether the identified skipped exon (SE) and retained intron (RI) events are biased towards causing frameshift compared to random SE and RI events.
***TT**: Predicted and may cause frameshift;
***TF**: Predicted and may not cause frameshift;
***FT**: Not predicted and may cause frameshift;
***FF**: Not predicted and may not cause frameshift

IDs of stress granule associated factors			
ATXN2	ELAVL1	LIN28A	RBPMS
ATXN2L	FMR1	LSM14A	RC3H1
BICD1	FXR1	MBNL1	RPS6KA3
CAPRIN1	FXR2	MCRIP1	RPTOR
CIRBP	G3BP1	MCRIP2	SSB
DCP1A	G3BP2	NANOS3	STAU1
DDX1	GIGYF2	NUFIP2	TIA1
DDX3X	GRB7	NXF1	TIAL1
DDX6	IGF2BP1	OGFOD1	TRAF2
DHX9	KHSRP	PABPC1	UBAP1
DYNC1H1	KIF5B	PABPC4	UBAP2L
EEF2	KLC1	PQBP1	VCP
EIF2S1	L1RE1	PUM1	YBX1
EIF4A1	LARP1	PUM2	ZBP1
EIF4E	LARP4	RACK1	ZFP36
EIF4G1	LARP4B	RBM4	

Supplementary Table 5: List of Stress granule associated factors from literature and from experimental validation in the laboratory

		Gene/Transcript upregulated by eutomers				Gene/Transcript downregulated by eutomers				NMD Transcript upregulated by eutomers				NMD Transcript ratio upregulated by eutomers				Splicing affected by low dose eutomers				Splicing affected by high dose eutomers			
		HCT116		HeLa		HCT116		HeLa		HCT116		HeLa		HCT116		HeLa		HCT116		HeLa		HCT116		HeLa	
NMD Transcript ID	Gene ID	T-202	T-595	T-202	T-595	T-202	T-595	T-202	T-595	T-202	T-595	T-202	T-595	T-202	T-595	T-202	T-595	T-202	T-595	T-202	T-595	T-202	T-595	T-202	T-595
	ATXN2					Yes		Yes												Yes			Yes		
ENST00000551755	ATXN2					Yes		Yes								Yes				Yes			Yes		
	ATXN2L																			Yes	Yes	Yes	Yes	Yes	Yes
ENST00000562583	ATXN2L									Yes	Yes	Yes	Yes	Yes	Yes					Yes	Yes	Yes	Yes	Yes	Yes
	BICD1			Yes	Yes																				
ENST00000395758	BICD1			Yes	Yes							Yes	Yes	Yes											
	CIRBP																				Yes		Yes		
ENST00000591055	CIRBP									Yes	Yes	Yes	Yes								Yes		Yes		
ENST00000586636	CIRBP									Yes	Yes		Yes								Yes		Yes		
ENST00000592234	CIRBP									Yes	Yes										Yes		Yes		
	DCP1A				Yes																				
	DYNC1H1																			Yes		Yes		Yes	
ENST00000462221	EIF2A									Yes	Yes	Yes	Yes												
	EIF2S1																					Yes			
	EIF4A1																					Yes		Yes	
ENST00000581544	EIF4A1									Yes	Yes	Yes	Yes									Yes		Yes	
	EIF4E					Yes		Yes																	
	EIF4G1																							Yes	
ENST00000442406	EIF4G1									Yes		Yes												Yes	
	FAM195A		Yes															Yes						Yes	
ENST00000491999	FAM195A		Yes							Yes	Yes	Yes	Yes	Yes		Yes	Yes	Yes						Yes	
	FMR1					Yes																			
ENST00000517947	G3BP1										Yes		Yes												
ENST00000522367	G3BP1											Yes													
	GIGYF2					Yes		Yes	Yes															Yes	
	GNB2L1																	Yes				Yes	Yes	Yes	Yes
ENST00000508682	GNB2L1									Yes	Yes	Yes	Yes					Yes				Yes	Yes	Yes	Yes
ENST00000504325	GNB2L1									Yes		Yes						Yes				Yes	Yes	Yes	Yes
	GRB7					Yes	Yes																		
	KIF5B					Yes																			
	KLC1								Yes									Yes	Yes			Yes	Yes	Yes	
	LARP1																				Yes	Yes		Yes	Yes
	LARP4					Yes		Yes																Yes	
	LARP4B					Yes		Yes																	
ENST00000476529	LARP4B					Yes		Yes			Yes				Yes										
	LSM14A					Yes														Yes		Yes			
	MBNL1					Yes																			
ENST00000525576	NXF1										Yes	Yes	Yes				Yes								
ENST00000565682	OGFOD1										Yes				Yes										
	PABPC1																	Yes			Yes				Yes
ENST00000523636	PABPC1									Yes	Yes	Yes	Yes					Yes			Yes				Yes
	PABPC4							Yes										Yes		Yes		Yes	Yes	Yes	Yes
ENST00000470443	PABPC4							Yes		Yes	Yes	Yes		Yes	Yes		Yes	Yes		Yes		Yes	Yes	Yes	Yes
	PQBP1																							Yes	
	PUM1																							Yes	
	PUM2					Yes																			
ENST00000432105	PUM2					Yes				Yes	Yes		Yes	Yes	Yes										
	RBPMS					Yes																Yes		Yes	Yes
ENST00000522694	RBPMS					Yes						Yes										Yes		Yes	Yes
	RPS6KA3					Yes		Yes	Yes																
	STAU1																	Yes							
	TIA1																							Yes	
ENST00000477044	TIA1									Yes		Yes												Yes	
ENST00000474809	TIA1											Yes	Yes											Yes	
	UBAP1			Yes																					
	ZFP36					Yes	Yes																		

Supplementary Table 6: Stress Granule associated genes affected by EIF4A3 inhibition
Genes and NMD transcripts (shaded with Ensembl IDs) that satisfy (Yes, shaded) the respective conditions on treatment with the eutomers (T-595 and T-202) in the respective cell lines (HCT116 or HeLa) - 1. Gene Upregulation, 2. Gene Downregulation, 3. Upregulated NMD transcripts, 4. Increasing NMD ratio, 5. Splicing affect by low doses or 6. Splicing affect by high doses.

a. NMD transcripts showing change in expression

Longest transcript shows upregulation

CBLL1	CCNB1IP1	TBRG1	PSPC1	RBM4B	NOD1	FAM76B	TAF1D	MTRR	EIF4A2	METTL22	
SRSF6	SHARPIN	H2AFX	HSCB	RBMX	HJURP	SRSF3	SRSF2	DBF4	E2F6	RNF8	
RABGEF1	TMEM189	DBF4B	SRPRB	WDR61	HUS1	USP3	UIMC1	CCNT2	CDK10	CLK1	
ALKBH1	H2AFJ	TAF4B	TUBE1	MAP1S	THUMPD2	HAUS2					

Longest transcript shows downregulation

CDK7	UBE2K	SEC24C	SMC1A	CHORDC1	POLG2	CDC27	CCNE2	RNF121	NUP35	RAB5A	UBA5
PUS7	LSM6	RINT1	KIF20A	ATG12	PDCD11	CCNL2	GTPBP8	SREK1	PTCD2	GADD45B	TMEM33
TMEM70	POLR1A	LMBRD1	TLK1	UBE2W	SNRNP27						

b. RBPs which also show changes in gene expression and NMD transcript changes

RBP genes which show overall monotonic increase in gene expression

SRSF2	SRSF6	RBM8A	SRSF3	PPRC1	RBMX	PTBP2	PABPC1L	CPEB3	PSPC1	RBM4B	MEX3D
ZFP36L2	MEX3C										

RBP genes which show overall monotonic DECcrease in gene expression

IGF2BP3	TARDBP	ZFP36	CPEB4	FMR1	SYNCRIP	SAMD4A	HNRNPR	PABPC4	RBMS1	ZFP36L1	MBNL1
RBM8A	SRSF4	ANKRD17	MBNL3	RBM47	ESRP2	RBFOX2	RBM24	RBMS3	HNRNPH2	RBMS2	

RBP genes whose NMD transcript increase (both expr and inc ratio)

SRSF6	RBMX	SART3	SRSF7	SRSF3	SRSF2	HNRNPA1	RBM38	SFPQ	QKI	HNRNPL	MBNL2
PABPC4	RBM4B	PSPC1	RBM41	ESRP2							

RBP genes whose NMD transcript increase (both expr and incl ratio) and this correlates with their overall monotonic increase in gene expression

SRSF2	SRSF6	SRSF3	RBMX	PSPC1	RBM4B

RBP genes whose NMD transcript increase (both expr and incl ratio) and this correlates with their overall monotonic deccrease in gene expression

PABPC4	ESRP2

Supplementary Table 7: Gene list showing a. EIF4A3 dependent NMD transcripts which also exhibit monotonic change in expression, and b. RBPs which also show changes in gene expression and NMD transcript changes

siRNA	Sense Sequence	Antisense Sequence
s18878	GCAUCUUGGUGAAACGUGAtt	UCACGUUUCACCAAGAUGCgg
s18876	GGAUAUUCAGGUUCGUGAAtt	UUCACGAACCUGAAUAUCCaa
s18877	CGAGCAAUCAAGCAGAUCAtt	UGAUCUGCUUGAUUGCUCGtt

Supplementary Table 8: Silencer Select siRNAs sequences (Life Technologies) targeting EIF4A3
