# Supplementary material for: Pharmacological systems analysis defines EIF4A3 functions in cell-cycle and RNA stress granule formation
Source: Commun Biol. 2019 May 3;2:165. doi: 10.1038/s42003-019-0391-9 (PMC6499833; doi:10.1038/s42003-019-0391-9)
Supplement: Supplementary file 2 — Description of Additional Supplementary Items [file 42003_2019_391_MOESM2_ESM.pdf]

Description of Additional Supplementary Items

Mazloomian et al

Supplementary Data 1: Properties of eIF4A3 inhibitor compounds.

Supplementary Data 2:

Raw Data for Fig 1b

HCT116_T_202: Normalized gene expression in HCT-116 cells treated with the various doses of T-202.

HCT116_T_595: Normalized gene expression in HCT-116 cells treated with the various doses of T-595.

HCT116_T_598: Normalized gene expression in HCT-116 cells treated with the various doses of T-598.

Hela_T_202: Normalized gene expression in HeLa cells treated with the various doses of T-202.

Hela_T_595: Normalized gene expression in HeLa cells treated with the various doses of T-595.

Hela_T_598: Normalized gene expression in HeLa cells treated with the various doses of T-598.

Supplementary Data 3:

Raw Data for Fig 1c

gene expression up sheet: Genes with monotonically increasing response profiles in at least one of the conditions are presented. The value of 1 means the gene was found in that specific condition, and 0 means the gene was not found.

gene expression down sheet: Genes with monotonically decreasing response profiles in at least one of the conditions are presented. The value of 1 means the gene was found in that specific condition, and 0 means the gene was not found.

Supplementary Data 4:

NMD expression up sheet: NMD prone transcripts with monotonically increasing response profiles (expression) in at least one of the conditions are presented. The value of "1" means the transcript was found in that specific condition, and "0" means the transcript was not found.

NMD ratio up sheet: NMD prone transcripts with monotonically increasing response profiles (gene normalized PSI values) in at least one of the conditions are presented. The value of "1" means the transcript was found in that specific condition, and "0" means the transcript was not found.

Supplementary Data 5:

Raw Data for Fig 2

MISO_all_events: All the alternatively spliced events as annotated by MISO when the respective cells lines were treated with the respective drugs.

VAST_all_events: All the alternatively spliced events as annotated by VAST-TOOLS when the respective cells lines were treated with the respective drugs.

MISO-siRNA-48: Alternative splicing events by type, Diff and Bayesfactor as annotated by MISO when EIF4A3 is knocked down by siRNA after 48 hr.

VAST-siRNA-48: Alternative splicing events by type as annotated by VAST-TOOLS when EIF4A3 is knocked down by siRNA after 48 hr.

overlap-sirna: The table of the gene names in HeLa cells corresponding to the splicing events which are identified by MISO on knock down of EIF4A3 by siRNA at 48 hr and when EIF4A3 is inhibited at high and low doses of T-202. A gene is considered alternatively splicing if it is affected by any of the splicing events.

Supplementary Data 6:

Raw Data for Fig 3a

HCT116_T_202.psi: Alternative splicing inclusion levels (see methods) on treatment with the various doses of T-202 in HCT-116 cells as determined by MISO

HCT116_T_202_filtered.psi: Filtered (see methods) alternative splicing inclusion levels on treatment with the various doses of T-202 in HCT-116 cells as determined by MISO

HCT116_T_595.psi Alternative splicing inclusion levels on treatment with the various doses of T-595 in HCT-116 cells as determined by MISO

HCT116_T_595_filtered.psi: Filtered alternative splicing inclusion levels on treatment with the various doses of T-595 in HCT-116 cells as determined by MISO

HCT116_T_598.psi Alternative splicing inclusion levels on treatment with the various doses of control distomer T-598 in HCT-116 cells as determined by MISO

HCT116_T_598_filtered.psi: Filtered alternative splicing inclusion levels on treatment with the various doses of control distomer T-598 in HCT-116 cells as determined by MISO

HeLa_T_202.psi: Alternative splicing inclusion levels (see methods) on treatment with the various doses of T-202 in HeLa cells as determined by MISO

HeLa_T_202_filtered.psi: Filtered (see methods) alternative splicing inclusion levels on treatment with the various doses of T-202 in HeLa cells as determined by MISO

HeLa_T_595.psi Alternative splicing inclusion levels on treatment with the various doses of T-595 in HeLa cells as determined by MISO

HeLa_T_595_filtered.psi: Filtered alternative splicing inclusion levels on treatment with the various doses of T-595 in HeLa cells as determined by MISO

HeLa_T_598.psi Alternative splicing inclusion levels on treatment with the various doses of control distomer T-598 in HeLa cells as determined by MISO

HeLa_T_598_filtered.psi: Filtered alternative splicing inclusion levels on treatment with the various doses of control distomer T-598 in HeLa cells as determined by MISO

Supplementary Data 7:

Raw Data for Fig 3b

HCT116_T_202.up: Ratio of alternative splicing events in monotonically increasing clusters when HCT-116 is treated with T-202

HCT116_T_202.down: Ratio of alternative splicing events in monotonically decreasing clusters when HCT-116 is treated with T-202

HCT116_T_595.up: Ratio of alternative splicing events in monotonically increasing clusters when HCT-116 is treated with T-595

HCT116_T_595.down: Ratio of alternative splicing events in monotonically decreasing clusters when HCT-116 is treated with T-595

HeLa_T_202.up: Ratio of alternative splicing events in monotonically increasing clusters when HeLa is treated with T-202

HeLa_T_202.down: Ratio of alternative splicing events in monotonically decreasing clusters when HeLa is treated with T-202

HeLa_T_595.up: Ratio of alternative splicing events in monotonically increasing clusters when HeLa is treated with T-595

HeLa_T_595.down: Ratio of alternative splicing events in monotonically decreasing clusters when HeLa is treated with T-595

Supplementary Data 8:

Raw Data for Fig 4

SE motif enrichment pval sheet: BH corrected p-values of motif enrichment analysis for SE events. Columns are explained in the header. Enrichment of motifs are shown by positive p-values and depletion of motifs by negative p-values.

RI motif enrichment pval sheet: BH corrected p-values of motif enrichment analysis for RI events. Columns are explained in the header. Enrichment of motifs are shown by positive p-values and depletion of motifs by negative p-values.

SE motif frequency sheet: The table represents the frequency of motif hits in SE regions of proteins in events with monotonic responses divided by the frequency of hits in background events. Only motifs with values <0.75 or >1.25 were considered as being significant candidates.

RI motif frequency sheet: The table represents the frequency of motif hits in RI regions of proteins in events with monotonic responses divided by the frequency of hits in background events. Only motifs with values <0.75 or >1.25 were considered as being significant candidates.

Y14 densities sheet: Normalized (against background) Y14 motif hit densities of monotonically increasing and decreasing response profiles and the respective distances to the splice junction.

Supplementary Data 9:

Raw data for Fig 5a

gene upreg pathways sheet: Genes found in pathway enrichment analysis are presented. FDR values for pathways in different data sets and whether a gene was found in an specific set are listed for pathway enrichment analysis of monotonically increasing genes.

gene downreg pathways sheet: Genes found in pathway enrichment analysis are presented. FDR values for pathways in different data sets and whether a gene was found in an specific set are listed for pathway enrichment analysis of monotonically decreasing genes.

NMD upreg transcript pathways sheet: FDR values for pathways in different data sets, and whether a gene was found in an specific set are listed for pathway enrichment analysis of genes corresponding to NMD prone transcripts with monotonically increasing expression.

NMD upreg ratio pathways sheet: FDR values for pathways in different data sets, and whether a gene was found in an specific set are listed for pathway enrichment analysis of genes corresponding to NMD prone transcripts with monotonically increasing gene normalized PSI values.

MISO low dose pathways sheet: Genes found in pathway enrichment analysis are presented. FDR values for pathways in different data sets, and whether a gene was found in an specific set are listed for pathway enrichment analysis of AS regulated genes at low drug concentrations.

MISO high dose pathways sheet: Genes found in pathway enrichment analysis are presented. FDR values for pathways in different data sets, and whether a gene was found in an specific set are listed for pathway enrichment analysis of AS regulated genes at high drug concentrations.

Supplementary Data 10:

Raw Data for Fig 5b

HCT116_T_202.up.stats sheet: Go ontology term enrichment and statistics of monotonically upregulated genes when HCT116 cells are treated with T-202.

HCT116_T_202.down.stats sheet: Go ontology term enrichment and statistics of monotonically downregulated genes when HCT116 cells are treated with T-202.

HCT116_T_595.up.stats sheet: Go ontology term enrichment and statistics of monotonically upregulated genes when HCT116 cells are treated with T-595.

HCT116_T_595.down.stats sheet: Go ontology term enrichment and statistics of monotonically downregulated genes when HCT116 cells are treated with T-595.

HeLa_T_202.up.stats sheet: Go ontology term enrichment and statistics of monotonically upregulated genes when HeLa cells are treated with T-202.

HeLa_T_202.down.stats sheet: Go ontology term enrichment and statistics of monotonically downregulated genes when HeLa cells are treated with T-202.

HeLa_T_595.up.stats sheet: Go ontology term enrichment and statistics of monotonically upregulated genes when HeLa cells are treated with T-595.

HeLa_T_595.down.stats sheet: Go ontology term enrichment and statistics of monotonically downregulated genes when HeLa cells are treated with T-595.

Supplementary Data 11:

Supporting data for Fig 5

hct116 GSEA sheet: Results of gene set enrichment analysis and clustering of BP terms for HCT116 cell line. Pathway names, FDR values, and the cluster names are listed.

HeLa GSEA sheet: Results of gene set enrichment analysis and clustering of BP terms for HeLa cell line. Pathway names, FDR values, and the cluster names are listed.

T202 GSEA sheet: Results of gene set enrichment analysis and clustering of BP terms for T-202 compound. Pathway names, FDR values, and the cluster names are listed.

T595 GSEA sheet: Results of gene set enrichment analysis and clustering of BP terms for T-595 compound. Pathway names, FDR values, and the cluster names are listed.

hct116 NMD GSEA sheet: Results of gene set enrichment analysis and clustering of BP terms for HCT116 cell line (for genes corresponding to NMD prone transcripts with monotonically increasing expressions). Pathway names, FDR values, and the cluster names are listed.

HeLa NMD GSEA sheet: Results of gene set enrichment analysis and clustering of BP terms for HeLa cell line (for genes corresponding to NMD prone transcripts with monotonically increasing expressions). Pathway names, FDR values, and the cluster names are listed.

T202 NMD GSEA sheet: Results of gene set enrichment analysis and clustering of BP terms for T-202 compound (for genes corresponding to NMD prone transcripts with monotonically increasing expressions). Pathway names, FDR values, and the cluster names are listed.

T595 NMD GSEA sheet: Results of gene set enrichment analysis and clustering of BP terms for T-595 compound (for genes corresponding to NMD prone transcripts with monotonically increasing expressions). Pathway names, FDR values, and the cluster names are listed.

hct116 NMD ratio GSEA sheet: Results of gene set enrichment analysis and clustering of BP terms for HCT116 cell line (for genes corresponding to NMD prone transcripts with monotonically increasing gene normalized PSI values). Pathway names, FDR values, and the cluster names are listed.

HeLa NMD ratio GSEA sheet: Results of gene set enrichment analysis and clustering of BP terms for HeLa cell line (for genes corresponding to NMD prone transcripts with monotonically increasing gene normalized PSI values). Pathway names, FDR values, and the cluster names are listed.

T202 NMD ratio GSEA sheet: Results of gene set enrichment analysis and clustering of BP terms for T-202 compound (for genes corresponding to NMD prone transcripts with monotonically increasing gene normalized PSI values). Pathway names, FDR values, and the cluster names are listed.

T595 NMD ratio GSEA sheet: Results of gene set enrichment analysis and clustering of BP terms for T-595 compound (for genes corresponding to NMD prone transcripts with monotonically increasing gene normalized PSI values). Pathway names, FDR values, and the cluster names are listed.

hct116 MISO GSEA sheet: Results of gene set enrichment analysis and clustering of BP terms for AS regulated genes found in HCT116 cell line. Pathway names, FDR values, and the cluster names are listed.

HeLa MISO GSEA sheet: Results of gene set enrichment analysis and clustering of BP terms for AS regulated genes found in HeLa cell line. Pathway names, FDR values, and the cluster names are listed.

all cell lines MISO GSEA sheet: Results of gene set enrichment analysis and clustering of BP terms for AS regulated genes found in the at least one of the cell lines. Pathway names, FDR values, and the cluster names are listed.

Supplementary Data 12: The overlap of the gene names in which differential splicing events were detected on treatment with siRNA in Wang *et al* (Wang) or our study (siRNA) and analyzed by the respective methods (D=DiffSplice, M=MISO, V=VAST-TOOLS).

Supplementary Data 13:

Raw data for Fig 6a

Treatment with T-595 Tables showing the percentage of cells in each phase of the cell cycle as determined by flow cytometry on a BD FACSVerse^{TM} as analyzed by ModFit software when HeLa cells were treated with various doses of T-595 and harvested at the indicated times. Where appropriate, the relevant statistics are shown (two-tail t-tests between control and experimental groups).

Treatment with siRNA Tables showing the percentage of cells in each phase of the cell cycle as determined by flow cytometry on a BD FACSVerse^{TM} as analyzed by ModFit software when HeLa cells were treated with various control (Control si#1,si#2) or targeting (EIF4A3 #1,#2,#3) siRNA and harvested at the indicated times. Where appropriate, the relevant statistics are shown (two-tail t-tests between control and experimental groups).

Supplementary Data 14:

Raw data for Fig 6b

Caspase Activity Data Tables showing the readings of caspase activity with HeLa cells were treated with increasing doses of T-595 or T-598 at 24 hr and 48 hr respectively.

R-script R-script used to generated the box plot from the raw data.

Supplementary Data 15:

Raw data for Fig 7a-c

Scanned blots Figures showing the whole blots and the cropped regions to support the western blotting data in Fig 6a-c

Supplementary Data 16:

Raw data for Fig 7d

Ave SG per cell Number of stress granules (SGs) per cell when HeLa cells were untreated or treated with T-595 or T-598 for 4 hour, with sodium arsenite added 1 hr prior to harvest.

Fraction of cells with >3 SG The number of cells (expressed as percentage of one untreated) displaying 3 or more SGs when untreated or treated with T-595 or T-598 for 24 hours, with sodium arsenite added 1 hr prior to harvest.
