# Supplementary material for: Pharmacological systems analysis defines EIF4A3 functions in cell-cycle and RNA stress granule formation
Source: Commun Biol. 2019 May 3;2:165. doi: 10.1038/s42003-019-0391-9 (PMC6499833; doi:10.1038/s42003-019-0391-9)
Supplement: Supplementary file 3 — Reporting Summary [file 42003_2019_391_MOESM3_ESM.pdf]

nature research

Corresponding author(s): Sam Aparicio

Last updated by author(s): 13/2/19

# Reporting Summary

Nature Research wishes to improve the reproducibility of the work that we publish. This form provides structure for consistency and transparency in reporting. For further information on Nature Research policies, see Authors & Referees and the Editorial Policy Checklist.

## Statistics

For all statistical analyses, confirm that the following items are present in the figure legend, table legend, main text, or Methods section.

| n/a | Confirmed | |
|---|---|---|
| ☐ | ☒ | The exact sample size (*n*) for each experimental group/condition, given as a discrete number and unit of measurement |
| ☐ | ☒ | A statement on whether measurements were taken from distinct samples or whether the same sample was measured repeatedly |
| ☐ | ☒ | The statistical test(s) used AND whether they are one- or two-sided<br>*Only common tests should be described solely by name; describe more complex techniques in the Methods section.* |
| ☐ | ☒ | A description of all covariates tested |
| ☐ | ☒ | A description of any assumptions or corrections, such as tests of normality and adjustment for multiple comparisons |
| ☐ | ☒ | A full description of the statistical parameters including central tendency (e.g. means) or other basic estimates (e.g. regression coefficient) AND variation (e.g. standard deviation) or associated estimates of uncertainty (e.g. confidence intervals) |
| ☐ | ☒ | For null hypothesis testing, the test statistic (e.g. *F*, *t*, *r*) with confidence intervals, effect sizes, degrees of freedom and *P* value noted<br>*Give P values as exact values whenever suitable.* |
| ☐ | ☒ | For Bayesian analysis, information on the choice of priors and Markov chain Monte Carlo settings |
| ☐ | ☒ | For hierarchical and complex designs, identification of the appropriate level for tests and full reporting of outcomes |
| ☐ | ☒ | Estimates of effect sizes (e.g. Cohen's *d*, Pearson's *r*), indicating how they were calculated |

*Our web collection on statistics for biologists contains articles on many of the points above.*

## Software and code

Policy information about availability of computer code

| | |
|---|---|
| Data collection | The RNA-seq libraries were sequenced on a HiSeq 2000 (Illumina, CA, USA) using the manufacturer's software and protocols. |
| Data analysis | RNA-seq reads were aligned with STAR aligner (STAR_2.5.1b_modified). SAMtools (version 0.3.3) was used to remove duplicate reads. Transcript abundances were estimated by Cufflinks (v2.2.1). The expression output values of Cufflinks were normalized by edgeR package (3.8.6). MISO (0.4.9) and VAST-TOOLS (1.0.0-beta.2) were used to perform the splicing analysis. WGCNA (v.1.51) framework was applied to cluster genes exhibiting correlated response patterns. Gene set enrichment analysis (GSEA) was carried out on a set of Biological Processes (BP) from MSigDB (v.5.2). For analyzing motif enrichment Position Weight Matrices (PWMs) were downloaded from CIS-BP RNA data base. R and Python were used to do the analysis and perform statistical tests also generating figures using ggplot2 and matplotlib accordingly. All the complementary codes written in python and R are uploaded to a github repository and are available. |

For manuscripts utilizing custom algorithms or software that are central to the research but not yet described in published literature, software must be made available to editors/reviewers. We strongly encourage code deposition in a community repository (e.g. GitHub). See the Nature Research guidelines for submitting code & software for further information.

## Data

Policy information about availability of data

All manuscripts must include a data availability statement. This statement should provide the following information, where applicable:

- Accession codes, unique identifiers, or web links for publicly available datasets
- A list of figures that have associated raw data
- A description of any restrictions on data availability

Raw sequence reads used in this study are available at the Short Read Archive (SRA) under the identifier SRP117312 and the BioProject identifier PRJNA401938. Most of data generated or analysed during this study are included in this published article (and its supplementary information files). All other data data that support the findings of this study are available from the corresponding author upon reasonable request.

# Field-specific reporting

Please select the one below that is the best fit for your research. If you are not sure, read the appropriate sections before making your selection.

☒ Life sciences ☐ Behavioural & social sciences ☐ Ecological, evolutionary & environmental sciences

For a reference copy of the document with all sections, see nature.com/documents/nr-reporting-summary-flat.pdf

# Life sciences study design

All studies must disclose on these points even when the disclosure is negative.

| | |
|---|---|
| Sample size | The number of samples and biological replicates was selected in to provide enough statistical power based on our previous experience. Four treatment conditions and two control conditions (2 different inhibitor compound and 1 inactive compound + 2 different cell lines for each compound) were considered for RNA-seq experiments. In each of these conditions 6 different treatment concentration of the inhibitor were applied. Graded treatments further enable solid detection of transcriptome responses. RNA-seq coverage was selected based on previous experience for allowing detection of alternative splicing events and expression changes. |
| Data exclusions | No data were excluded |
| Replication | Two different cell lines and 2 active inhibitor compounds (and 1 inactive compound) were used independently (4 pairs of conditions) to confirm the reproducibility of our results in the presence of biological replicates. Additionally, 5 inhibitor concentration (and one control) were experimented in each case to better trace and interpret the results. The findings were compared both qualitatively and quantitatively, and also were compared with siRNA knockdowns and selected examples validated by published literature. |
| Randomization | This does not apply to our study. Here data allocation was done based on the presence or absence of EIF4A3 inhibitor or siRNA targeted to EIF4A3 transcript versus controls. |
| Blinding | Complete Blinding was hard to achieve to the current study. However, the following measures were taken to ensure data integrity: the data collection was done by a one set of investigators. The results were then passed through standardized bioinformatic pipelines and analysed by a dedicated bioinformatician (AM). The biological validation was performed by a different investigator. For the assaying of SGs, the researcher (AEN) was provided the drugs but not told which one was the active eutomer and which the inactive distomer. The drugs were unmasked for the repeat experiments. |

# Reporting for specific materials, systems and methods

We require information from authors about some types of materials, experimental systems and methods used in many studies. Here, indicate whether each material, system or method listed is relevant to your study. If you are not sure if a list item applies to your research, read the appropriate section before selecting a response.

## Materials & experimental systems

| n/a | Involved in the study |
|---|---|
| ☐ | ☒ Antibodies |
| ☐ | ☒ Eukaryotic cell lines |
| ☒ | ☐ Palaeontology |
| ☒ | ☐ Animals and other organisms |
| ☒ | ☐ Human research participants |
| ☒ | ☐ Clinical data |

## Methods

| n/a | Involved in the study |
|---|---|
| ☒ | ☐ ChIP-seq |
| ☐ | ☒ Flow cytometry |
| ☒ | ☐ MRI-based neuroimaging |

## Antibodies

| | |
|---|---|
| Antibodies used | The following antibodies were used in the current study: G3BP (611126, BD Transductions, 1:1000), GRB2 (610111, BD Transductions, 1:1000), TIA-1 (ab40693, rabbit polyclonal, Abcam, 1:1000), YB-1 (59-Q)(sc-101198, Santa Cruz Biotech, 1:500), CENP-B (sc-22788, Santa Cruz, Dallas, TX) and alpha tubulin (T9026, Sigma-Aldrich, St. Louis, MO). |
| Validation | CENP-B : validated for use in immunofluorescence (IF) assays : https://www.scbt.com/scbt/product/cenp-b-antibody-h-65<br>alpha-tubulin : validated for use in IF assays : https://www.sigmaaldrich.com/catalog/product/sigma/t9026?lang=en&region=CA<br>G3BP : validated for Western Blotting (WB) and IF assays : http://www.bdbiosciences.com/ca/reagents/research/antibodies-buffers/cell-biology-reagents/cell-biology-antibodies/purified-mouse-anti-human-g3bp-23g3bp/p/611126<br>GRB2 : validated for WB : http://www.bdbiosciences.com/ca/applications/research/b-cell-research/intracellular-antigens/human/purified-mouse-anti--grb2-81grb2/p/610111<br>TIA-1 : validated for WB and IF : https://www.abcam.com/tia1-antibody-c-terminal-ab40693.html |

# Eukaryotic cell lines

Policy information about cell lines

| | |
|---|---|
| Cell line source(s) | Both cell lines were sourced commercially from ATCC; HeLa (ATCC CCL-2) and HCT1-116 (ATCC CCL-247). |
| Authentication | The HeLa cell line used found by STR testing (Genetica, NC, USA) to be 100 % matching to ATCC CCL-2 HeLa. The HCT-116 cell line was a 76 % match to ATCC CCL-247 HCT-116 due to genomic instability as this line is known to be mismatch repair deficient and hence microsatellite unstable (https://www.ncbi.nlm.nih.gov/pmc/articles/PMC5249119/). This does not affect the conclusions of the study since we used two unrelated (cervical and colorectal) mammalian cell lines to control for line specific effects. Furthermore, the methodology employed showed results that have been validated against existing literature. |
| Mycoplasma contamination | All cell lines used in this study were routinely tested for mycoplasma and in the latest test (May 2018), all were found to be negative. |
| Commonly misidentified lines (See ICLAC register) | None were used in this study. |

# Flow Cytometry

## Plots

Confirm that:

☒ The axis labels state the marker and fluorochrome used (e.g. CD4-FITC).

☒ The axis scales are clearly visible. Include numbers along axes only for bottom left plot of group (a 'group' is an analysis of identical markers).

☒ All plots are contour plots with outliers or pseudocolor plots.

☒ A numerical value for number of cells or percentage (with statistics) is provided.

## Methodology

| | |
|---|---|
| Sample preparation | HeLa cells were treated with 1 or 5 μM of T-595 or transfected with each single siRNA (#1, #2, or #3) against EIF4A3 or control siRNA (#1 or #2) for the indicated time. Cells were washed twice with ice-cold PBS, and treated with Cycle Test Plus DNA Reagents (BD Biosciences, Franklin Lakes, NJ) in accordance with the manufacturer's instructions. |
| Instrument | BD FACSVerse™ |
| Software | The cellular DNA contents were determined by flow cytometry and the percentage of cells in each phase of the cell cycle was analyzed by ModFit software (Verity, Topsham, ME). |
| Cell population abundance | The abundance of cells in the post-sort fraction was typically over 90% of the population by analysis of ModFit software. |
| Gating strategy | The singlet cell region of the FL2-A vs. FL2-W dot plot was gated (R1) as shown in the Supplementary Figure. FL2-W was used to discriminate singlet and aggregated cells. |

☒ Tick this box to confirm that a figure exemplifying the gating strategy is provided in the Supplementary Information.
