# Supplementary material for: Pharmacological systems analysis defines EIF4A3 functions in cell-cycle and RNA stress granule formation
Source: Commun Biol. 2019 May 3;2:165. doi: 10.1038/s42003-019-0391-9 (PMC6499833; doi:10.1038/s42003-019-0391-9)
Supplement: Supplementary file 18 — Supplementary Data 15 [file 42003_2019_391_MOESM18_ESM.pdf]

Hela Cells

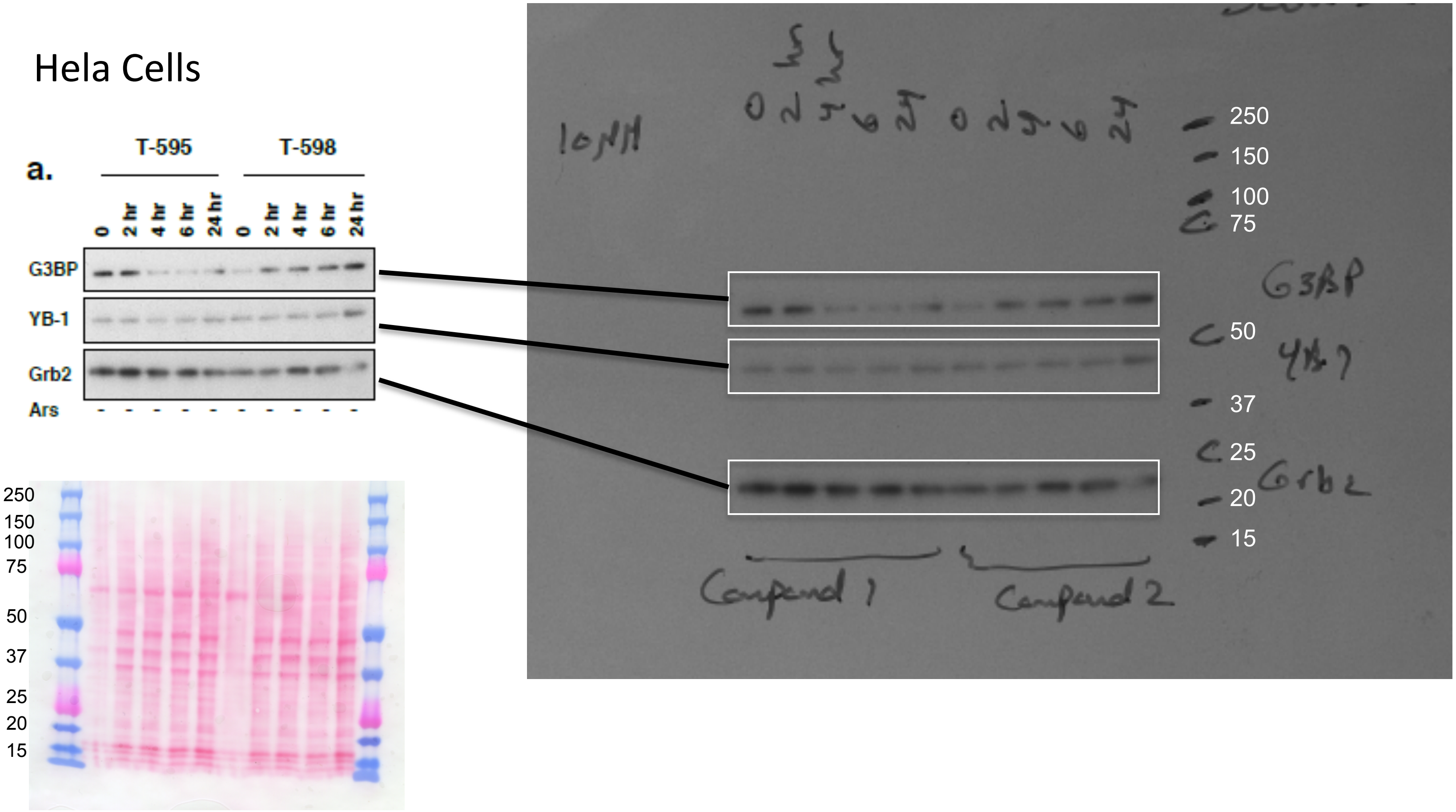

a.
T-595
T-598
0
2 hr
4 hr
6 hr
24 hr
G3BP
YB-1
Grb2
Ars
250
150
100
75
50
37
25
20
15
10nM
Compound 1
Compound 2

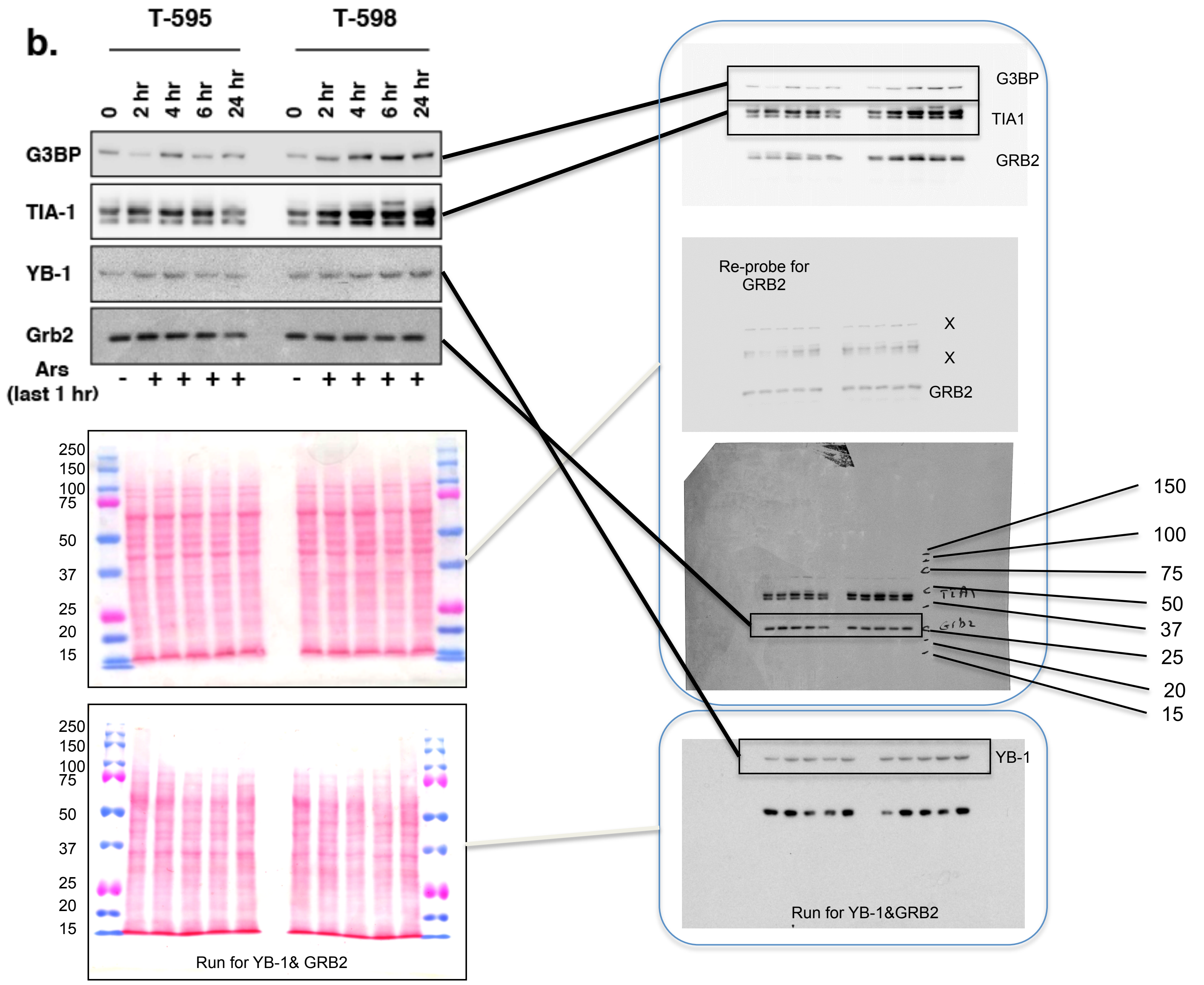

b.
T-595
T-598
0
2 hr
4 hr
6 hr
24 hr
G3BP
TIA-1
YB-1
Grb2
Ars
(last 1 hr)
- + + + + - + + + +
250
150
100
75
50
37
25
20
15
Run for YB-1& GRB2
G3BP
TIA1
GRB2
Re-probe for GRB2
X
X
GRB2
150
100
75
50
37
25
20
15
YB-1
Run for YB-1&GRB2

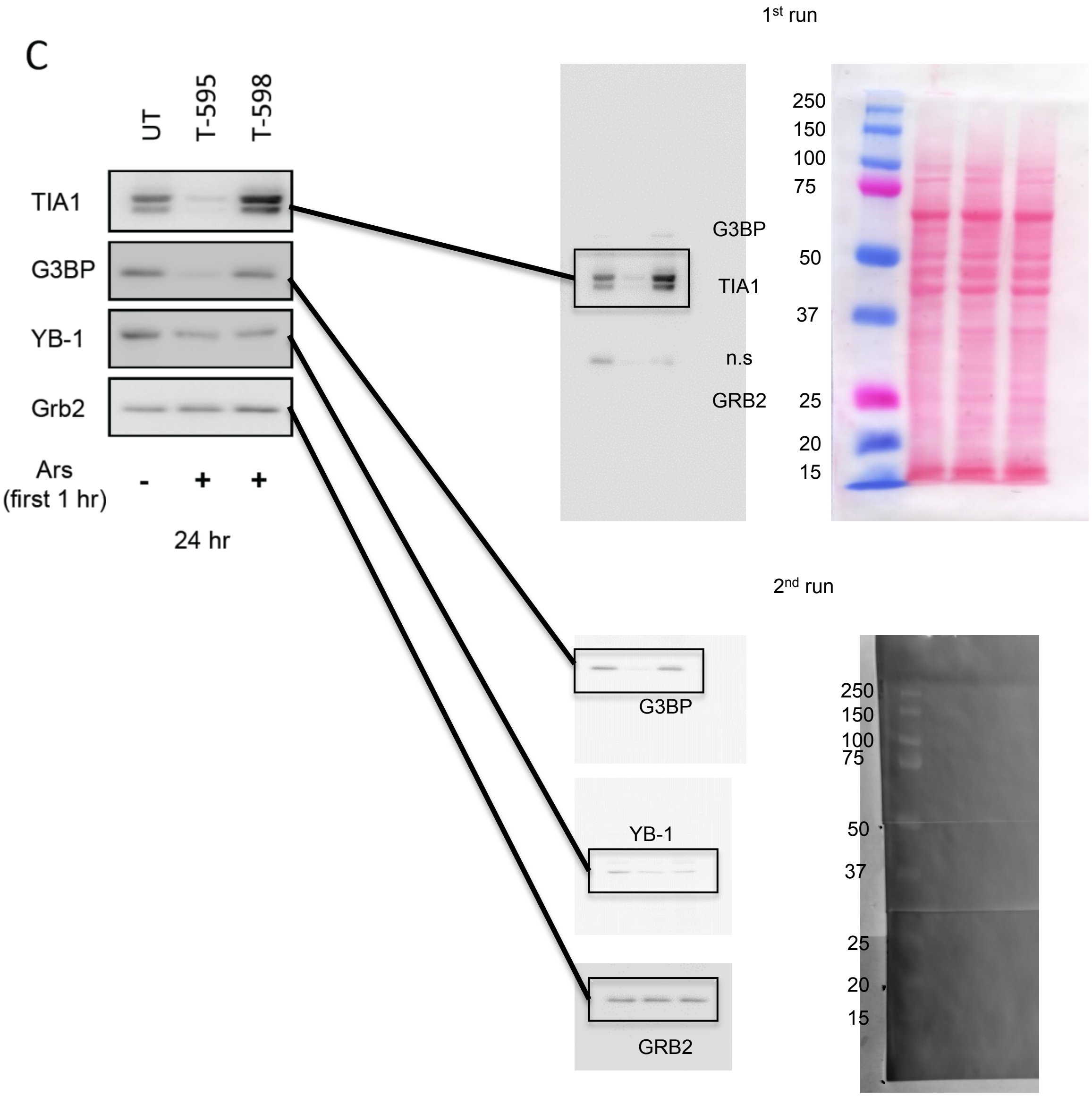
C
UT
T-595
T-598
TIA1
G3BP
YB-1
Grb2
Ars
(first 1 hr)
-
+
+
24 hr
1st run
250
150
100
75
G3BP
50
TIA1
37
n.s
GRB2
25
20
15
2nd run
G3BP
YB-1
GRB2
250
150
100
75
50
37
25
20
15
